# Anticancer Activity of Miswak Root Extract in Breast Cancer Cell Line: HRLC-MS/MS Profiling, In Vitro Evaluation, and In Silico Analysis

**DOI:** 10.3390/ijms27135751

**Published:** 2026-06-25

**Authors:** Abrar Turki, Md. Abul Barkat, Yasmin Basheer Ahmed, Harshita Barkat, Raghad Rashed Alotaibi, Khursheed Ahmad, Rumana Ahmad, Sahabjada Siddiqui

**Affiliations:** 1 Clinical Nutrition Department, College of Applied Medical Sciences, University of Hafr Al Batin, Hafr Al Batin 39524, Saudi Arabia; 2 Department of Pharmaceutics, College of Pharmacy, University of Hafr Al Batin, Hafr Al Batin 39524, Saudi Arabia; 3 Department of Biotechnology, Era’s Lucknow Medical College and Hospital, Era University, Lucknow 226003, India; 4 Department of Biochemistry, Era’s Lucknow Medical College and Hospital, Era University, Lucknow 226003, India

**Keywords:** Miswak root, HRLC-MS/MS characterization, anti-cancer potential, MCF-7 breast cancer cell line, computational analysis

## Abstract

Breast cancer is among the most commonly diagnosed malignancies in women and remains difficult to treat due to therapy resistance and the adverse effects associated with conventional chemotherapeutic regimens. In this study, the anticancer activity of the ethanolic root extract of *Salvadora persica* (*S. persica*), commonly known as Miswak, was evaluated in human breast cancer cells using a combination of in vitro assays, phytochemical profiling, and computational analyses. HRLC-MS/MS characterization revealed a wide range of bioactive constituents, including alkaloids, flavonoid derivatives, glucosinolates, and fatty acid–based molecules detected under both ionization modes. The extract exhibited a concentration-dependent cytotoxic effect on breast cancer MCF-7 and MDA-MB-231 cells, with IC_50_ values of 144.1 and 176.3 µg/mL, respectively, as determined by the MTT assay, while exerting negligible toxicity toward normal Vero cells. Miswak extract enhanced intracellular ROS production, disruption of MMP, nuclear condensation, and increased apoptotic cell populations, along with S-phase cell cycle arrest, pointing toward activation of mitochondrial-mediated apoptosis. In silico docking results indicated that key phytoconstituents exhibit strong binding interactions with multiple breast cancer–relevant targets such as ERα, PR, EGFR, HER3, IGF-1R, and GPER. Additionally, pharmacokinetic and toxicity predictions suggested favorable drug-like properties with minimal safety concerns. Thus, these findings support its potential as a promising plant-derived therapeutic candidate for breast cancer.

## 1. Introduction

Breast cancer continues to be one of the most commonly diagnosed malignancies among women worldwide and poses a major burden on global health systems. In India, it represents the leading cancer among women, accounting for approximately 27–32% of all female cancers, with a mortality rate of about 12.7 per 100,000 women (https://cytecare.com/blog/breast-cancer/statistics-of-breast-cancer/; accessed on 28 May 2026). Similarly, in the United States, nearly 13% of women are expected to develop invasive breast cancer during their lifetime, and a substantial proportion of new cancer diagnoses each year occur in females (https://www.cancer.org/cancer/types/breast-cancer/about.html; accessed on 30 May 2026). A significant clinical concern is that nearly one-third of patients eventually develop metastatic disease, wherein the cancer spreads to distant organs such as bone, liver, and lungs, leading to poor prognosis and increased mortality. The rising incidence of breast cancer globally can be attributed to factors such as changing lifestyles, aging populations, genetic susceptibility, and environmental exposures. Among its various subtypes, hormone receptor–positive breast cancers, characterized by the expression of estrogen receptor alpha (ERα) and progesterone receptor (PR), constitute a major proportion of cases, comprising approximately 70% to 80% of all breast cancer diagnoses and serve as critical targets for endocrine-based therapies, as these receptors regulate tumor growth, survival, and progression [1].

Despite considerable progress in early detection and therapeutic strategies, including chemotherapy, surgery, radiotherapy, targeted therapy, and hormonal interventions, effective management of breast cancer remains challenging [2]. Conventional chemotherapeutic agents often lack specificity, leading to toxicity in normal tissues and a wide range of adverse effects. Furthermore, issues such as multidrug resistance, tumor relapse, and metastatic progression significantly limit long-term treatment success [3]. These challenges highlight the need to develop safer, more selective, and more efficacious therapeutic options that minimize damage to healthy tissues while precisely targeting cancer cells. In this context, natural products derived from medicinal plants have emerged as a valuable resource for anticancer drug discovery. Historically, several plant-derived compounds such as paclitaxel, vincristine, and camptothecin analogs have been successfully developed into clinically effective anticancer agents [4]. The biological effects of phytochemicals are diverse and include anti-inflammatory, antioxidant, antiproliferative, and pro-apoptotic effects. Their anticancer potential is often mediated through the modulation of critical cellular processes, such as disruption of mitochondrial membrane potential (MMP), reactive oxygen species (ROS) generation, activation of apoptotic pathways, and regulation of cell cycle checkpoints [5,6]. These multifaceted mechanisms make plant-derived compounds promising candidates for novel cancer therapeutics.

*Salvadora persica* L., (*S. persica*) commonly known as miswak or the toothbrush tree, is a well-known medicinal plant distributed across regions of the Middle East, Africa, and South Asia. Traditionally, its roots and twigs have been widely used for oral hygiene due to their natural antimicrobial properties. Beyond dental care, *S. persica* has been employed in traditional medicine for managing a variety of conditions, including infections, inflammatory disorders, gastrointestinal disturbances, and metabolic ailments [7]. Phytochemical studies have identified a diverse array of bioactive constituents in this plant, such as alkaloids, flavonoids, phenolic acids, terpenoids, fatty acids, and sulfur-containing compounds [8]. These constituents contribute to its reported pharmacological activities, including antioxidant, anti-inflammatory, antimicrobial, and cytoprotective effects [7,8]. Previous studies on *S. persica* roots have predominantly focused on its antimicrobial, antioxidant, anti-inflammatory, and oral healthcare properties [7]. Although a recent investigation reported the anticancer potential of *S. persica* root extract against hepatocellular carcinoma (HepG2) cells [9], its effects on breast cancer and underlying molecular mechanisms have not yet been explored. To the best of our knowledge, no study has comprehensively evaluated the anticancer efficacy of *S. persica* root extract against breast cancer cells through integrated phytochemical profiling, mechanistic in vitro investigations, molecular docking, and pharmacokinetic analyses. Therefore, the present study was undertaken to address this knowledge gap and to provide a comprehensive assessment of the anticancer potential of *S. persica* root extract in breast cancer.

Human breast cancer MCF-7 cell line is a well-established model for investigating oxidative stress-induced, mitochondria-dependent apoptosis and is characterized by the presence of hormone receptors such as ERα, PR, and IGF-1R [10,11]. Therefore, the present study was undertaken to evaluate the anticancer efficacy of the ethanolic root extract of *S. persica* against human breast cancer MCF-7 cells. The investigation further aimed to elucidate the mechanistic aspects of its cytotoxic effects by assessing intracellular ROS generation, MMP disruption, induction of apoptosis, and alterations in cell cycle progression. Additionally, comprehensive phytochemical profiling of the extract was performed using high-resolution liquid chromatography-mass spectrometry (HRLC-MS/MS), followed by molecular docking studies to analyze the interaction of identified bioactive compounds with key molecular targets implicated in breast cancer. This combined experimental and computational approach is expected to provide deeper insights into the therapeutic potential of miswak and support its development as a source of novel anticancer agents.

## 2. Results

### 2.1. HRLC-MS/MS Analysis of S. persica Root Extract in Positive Ion Mode

**Figure 1:**
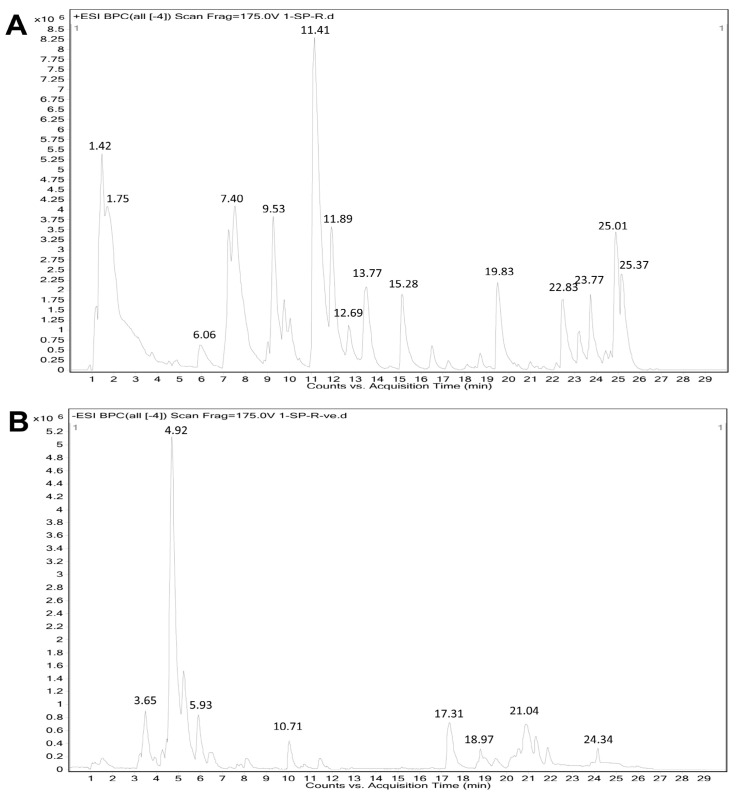
HR-LCMS chromatogram of *S. persica* root extract with retention time and base intensity value of observed chemical component at (**A**) at positive ion mode, (**B**) at negative ion mode.

This HPLC-ESI-MS/MS analysis comprehensively identifies various biologically important compounds such as antioxidants, alkaloids, terpenoids, fatty acids, and peptide derivatives. The retention times, scores, accurate masses, and *m*/*z* values help to confirm the identity and ensure the reliability of detection. The untargeted analysis of *S. persica* root extract was performed for positive and negative ion modes. The HRLC-MS/MS analysis of *S. persica* root extract in positive ion mode led to the identification of diverse phytochemicals belonging to multiple chemical classes, including alkaloids, peptides, and fatty amides. A total of fifteen compounds were characterized based on their retention time (RT), exact mass, molecular formula, and *m*/*z* values, indicating the chemical complexity of the extract (Table 1, Figure 1A). The wide range of chemical classes identified, including alkaloids, peptides, terpenes, fatty amides, and glycosides, demonstrates the rich phytochemical diversity of *S. persica* root extract. This diversity may contribute to its broad spectrum of biological activities and support its traditional medicinal use.

### 2.2. HRLC-MS/MS Analysis of S. persica Root Extract in Negative Ion Mode

The HRLC-MS/MS analysis of *S. persica* root extract in negative ion mode led to the identification of diverse phytochemicals belonging to multiple chemical classes, wherein the majority of compounds are acidic metabolites (phenolics, carboxylic acids). A total of seven compounds were characterized based on their retention time (RT), exact mass, molecular formula, and *m*/*z* values, indicating the chemical complexity of the extract (Table 2, Figure 1B). The wide range of chemical classes identified, including flavonoid glycoside, isothiocyanate, and fatty acid derivatives, demonstrates the rich phytochemical diversity of *S. persica* root extract. This diversity may contribute to its broad spectrum of biological activities and supports its traditional medicinal use (Table 2).

### 2.3. Effect of S. persica Root Extract on Cellular Morphology and Cell Viability

**Figure 2:**
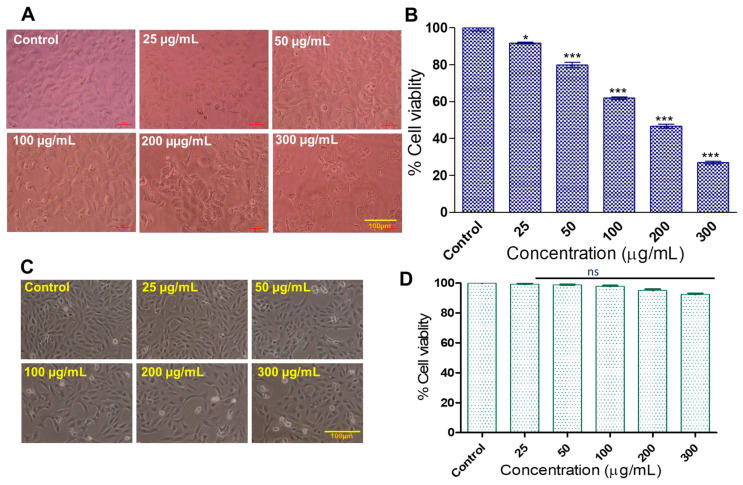
Cytotoxic test of *S. persica* root extract on human breast cancer MCF-7 cells. (**A**) Analysis of morphological changes in MCF-7 cells subjected to *S. persica* root extract, the concentrations ranging from 25 to 300 µg/mL, under an inverted microscope (**B**). Cytotoxicity of *S. persica* root extract was quantified as the percentage cell viability of MCF-7 cells at 24 h. (**C**) Analysis of morphological changes in Normal Vero cells at different doses of *S. persica* root extract under an inverted microscope (**D**) Percentage cell viability of Vero cells at different doses of *S. persica* root extract. Values from a minimum of three independent experiments are shown as Mean ± SD, with * *p* < 0.05 and *** *p* < 0.001 in comparison to the control, while ns = non-significant.

MCF-7 human breast cancer cells were exposed for 24 h to increasing concentrations (25–300 µg/mL) of *S. persica* root extract. Figure 2A,B show the percent cell viability of MCF-7 cells at various concentrations of *S. persica* root extract after 24 h. *S. persica* root extract treatment gave noteworthy structural differences when compared to the untreated MCF-7 cells. Untreated cells exhibited the usual characteristics of attached, homogenous, and even cell surface, whereas treated cells became non-attached and spherical in morphology (Figure 2A). As indicated in Figure 2B, in a dose-dependent manner, *S. persica* root dramatically decreased the viability of breast cancer cells. *S. persica* root extract has an inhibitory concentration (IC_50_) of 144.1 µg/mL for the decrease in MCF-7 cell count, while it was calculated to be 176.3 µg/mL for MDA-MB-231 cells (Figure 2 and Figure S1). Thus, *S. persica* root extract produced a more pronounced inhibitory effect in MCF-7 cells than in MDA-MB-231 cells. Furthermore, the Trypan Blue exclusion assay independently validated the cytotoxicity findings obtained from the MTT assay (Figure S2). Consistent with the MTT results, *S. persica* root extract exerted a greater inhibitory effect on MCF-7 cells compared with MDA-MB-231 cells. The IC_50_ values determined by the Trypan Blue assay were 143 μg/mL for MCF-7 cells and 177 μg/mL for MDA-MB-231 cells, comparable to those obtained from the MTT assay. These findings confirm that the observed reduction in cell viability reflects cytotoxic activity rather than solely alterations in cellular metabolic activity. Since the MCF-7 cell line is widely used as a reliable model for studying oxidative stress–driven, mitochondria-mediated apoptosis and is distinguished by the expression of hormone receptors, including ERα, PR, and IGF-1R, a further study was conducted using this cell line. The aberrant morphological change and decrease in cell viability were not seen in the exposed Vero normal cells (Figure 2C,D). A small percentage of normal cells showed a non-adherent tendency to high *S. persica* root extract doses after being exposed for 24 h. Based on the IC_50_ value of *S. persica* root extract against MCF-7 cells, for additional in vitro cell death investigation, three effective dosages were determined: low dose (LD = 50 µg/mL), medium dose (MD = 100 µg/mL), and high dose (HD = 200 µg/mL).

### 2.4. Chromatin Condensation Assay of S. persica Root Extract Against MCF-7 Cells

**Figure 3:**
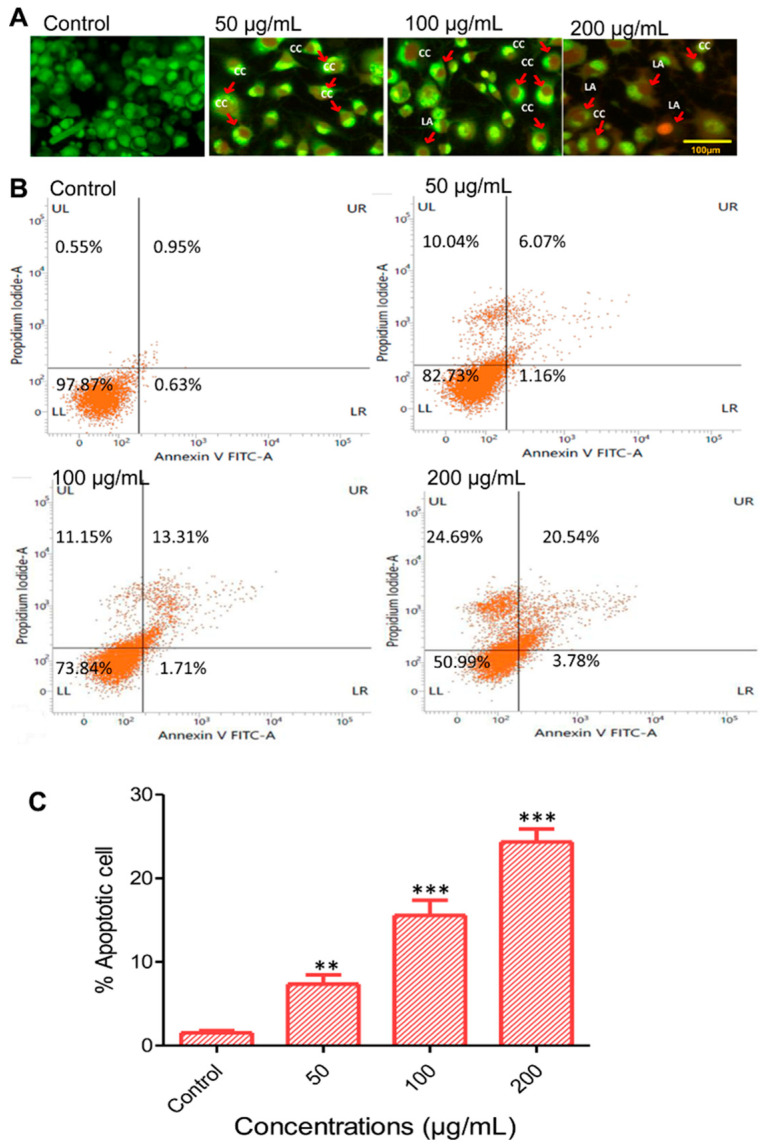
It shows how *S. persica* root extract induces apoptosis in human breast cancer MCF-7 cells. (**A**) Fluorescent photomicrographs of MCF-7 cells double-stained with AO/EtBr at 50, 100, and 200 μg/mL of *S. persica* root extract after 24 h. VC stands for viable cells, CC for chromatin condensation, and LA for late apoptosis. 100 μm is the scale bar. (**B**) Flow cytometry analysis employing annexin V/FITC and PI double stain following a 24 h treatment with three effective dosages of *S. persica* root extract. The population of viable (annexin V-PI-), early apoptotic (annexin V+ PI-), late apoptotic (annexin V+ PI+), and necrotic (annexin V-PI+) cells is depicted in representative figures. (**C**) Graph showing the total percentage of total apoptotic cells analyzed by the flow cytometer. ** *p* < 0.01 and *** *p* < 0.001 when compared to the control group (*n* = 3).

The AO/EtBr double stain showed that control cells had evenly nucleated green coloring, indicating living and healthy cells, shown in the photomicrograph (Figure 3A). Early apoptosis was indicated by green-colored cells with condensed nuclei, while late apoptosis was indicated by orange-red-colored cells with condensed nuclei. Moreover, annexin-V/FITC-PI double stain revealed that the percentageof viable cells (annexin V- and PI-) was decreased depending upon the dose. The untreated cells showed a 97.87% survival rate and were deemed alive and healthy. Cell death from *S. persica* root extract increased to 1.16% early apoptotic and 6.07% late apoptotic at 50 μg/mL, 1.71% early apoptotic and 13.31% late apoptotic at 100 μg/mL, and 3.78% early apoptotic and 20.54% late apoptotic at 200 μg/mL (Figure 3B,C). Early apoptotic characteristics were seen in MCF-7 cells at low doses of the *S. persica* root extract, while late apoptotic characteristics were seen at high doses.

### 2.5. S. persica Root Induces Intracellular ROS Generation Against MCF-7 Cells

**Figure 4:**
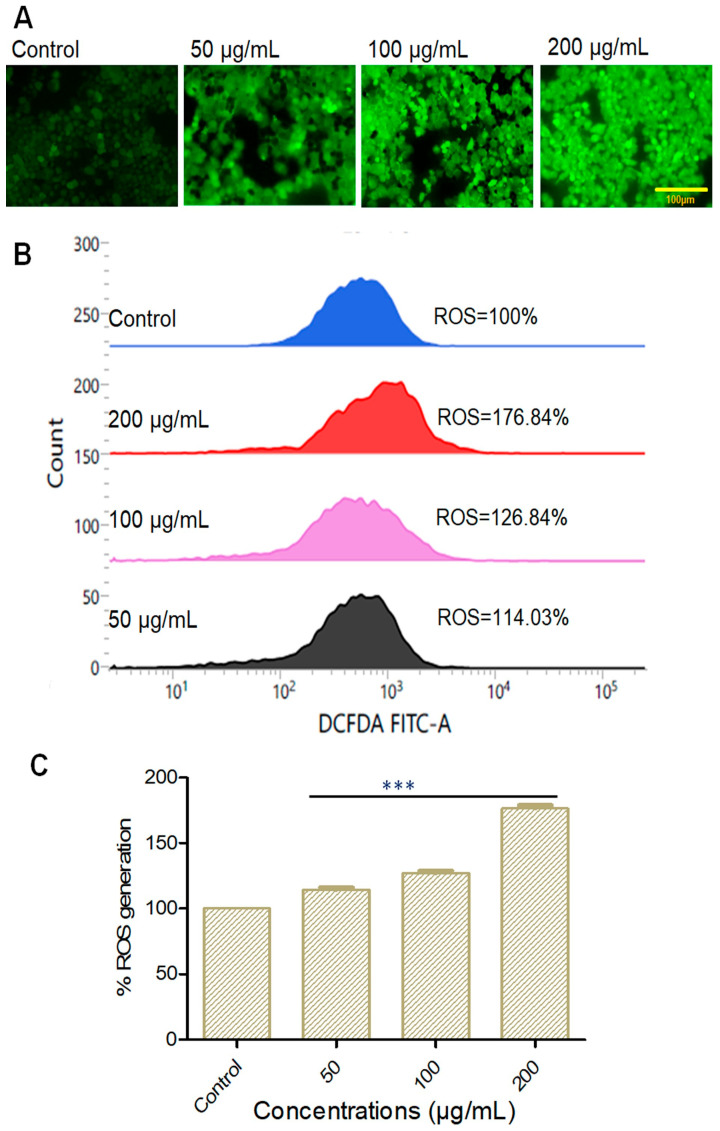
Intracellular ROS production in MCF-7 cells. (**A**) A fluorescence microscope image of intracellular ROS production caused by three effective *S. persica* root doses following a 12 h incubation period. (**B**) A quantitative assessment of the cells’ fluorescence expressed as the percentage of ROS production determined by a flow cytometer. (**C**) Graph showing percentage ROS generation as compared to control. *** *p* < 0.001 when compared to the control group (*n* = 3).

The photomicrographs of Figure 4A revealed that, in contrast to untreated breast cancer cells MCF-7, ROS intensity was increased notably when MCF-7 cells were treated with *S. persica* root extract. The quantitative estimation using a flow cytometer revealed that control cells showed a very low percentage of ROS, consistent with the low ROS intensity typically seen in healthy cells. However, *S. persica* root induced ROS generation levels notably to 14.03%, 26.84%, and 76.84% at 50, 100, and 200 μg/mL concentrations, respectively (Figure 4B,C).

### 2.6. Analysis of MMP Loss by S. persica Root Extract Against MCF-7 Cells

**Figure 5:**
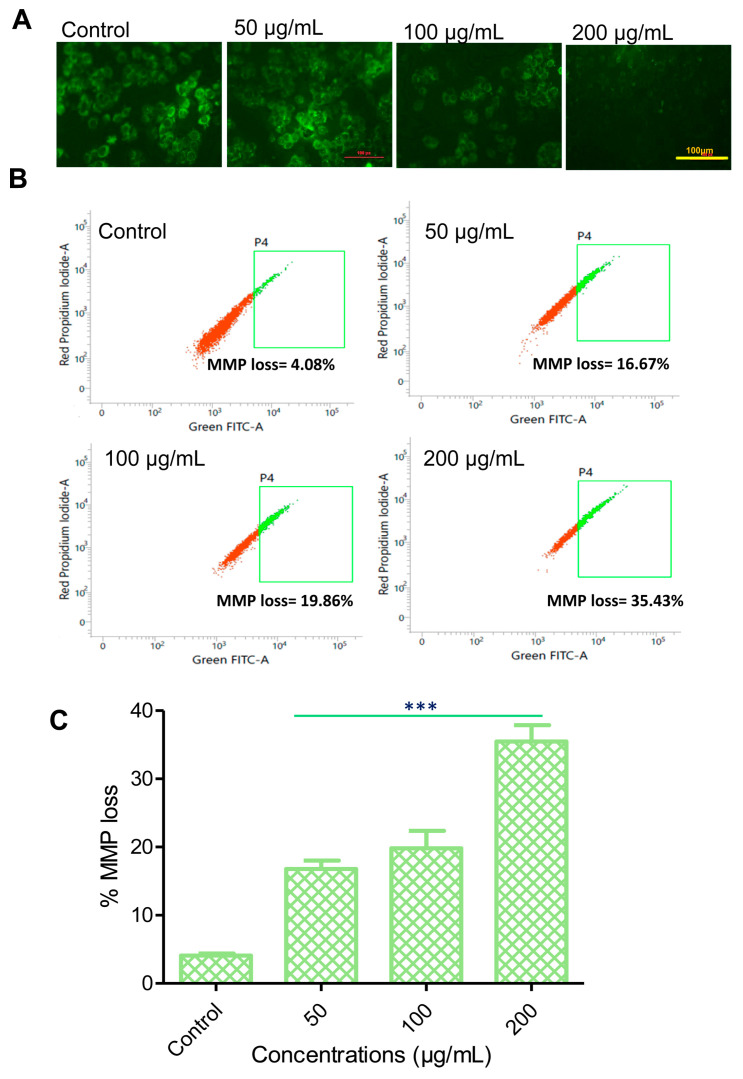
MMP loss of breast cancer cells MCF-7 stained with Rh 123 dye. (**A**) Representative photomicrographs show the decrease in MMP fluorescent intensity depending upon dose. (**B**) The flow-cytometer evaluation exhibits the percentage of MMP loss at different doses of *S. persica* root extract. (**C**) Graph showing the percentage of MMP loss as compared to the control. *** *p* < 0.001 when compared to the control group (*n* = 3).

The fluorescence microscopic analysis revealed high MMP in untreated cells, while the fluorescence level was decreased at high concentration (Figure 5A). The flow cytometry data showed a high MMP loss in treated MCF-7 cells of breast cancer in contrast to untreated cells. The untreated breast cancer cells exhibited 4.08% MMP loss, while *S. persica* root extract treatment at 50, 100, and 200 μg/mL concentrations on MCF-7 cells showed the loss of MMP to 16.67%, 19.86%, and 35.43%, respectively (Figure 5B,C).

### 2.7. S. persica Root Extract Induces Cell Cycle Arrest in MCF-7 Cells

**Figure 6:**
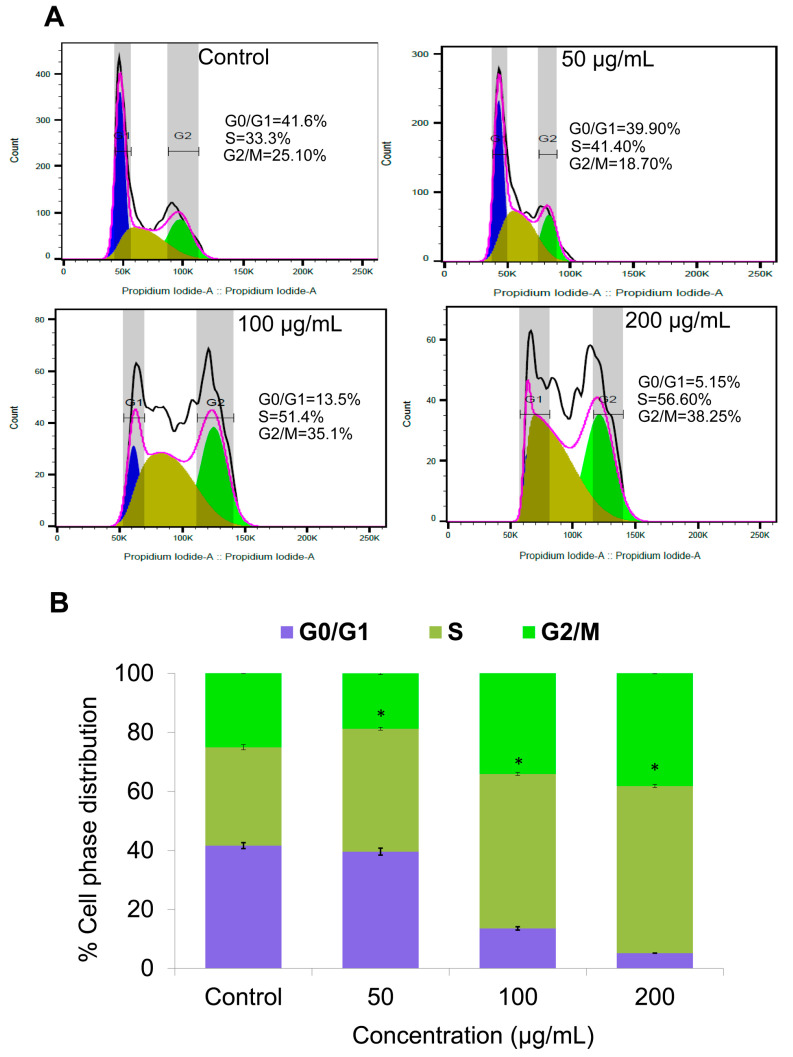
MCF-7 cells’ DNA content was determined using flow cytometry. (**A**) A graph illustrating the average percentage of cells in each stage of the cell cycle after a 24 h treatment with three effective doses of *S. persica* root extract. (**B**) A histogram displays the average percentage of cells in each stage of the cell cycle. * *p* < 0.05 when compared to the control group (*n* = 3).

Cell cycle analysis by flow cytometry was performed on MCF-7 cells after 24 h exposure to *S. persica* root extract. As depicted in Figure 6A,B, exposure to *S. persica* root extract greatly increased the proportion of MCF-7 cells in S phase while decreasing the number of cells in the G0/G1 phase. There was a potential increase in the proportion of cells in the S phase of the cell cycle after exposure to *S. persica* root extract. These results imply that the S checkpoints of the cell cycle were triggered by *S. persica* root extract in MCF-7 cells.

### 2.8. Molecular Docking and Interaction Studies of Detected Phytochemicals at Positive ESI

The molecular docking study was conducted to evaluate the binding potential of phytoconstituents identified in the positive ion mode against multiple therapeutic targets, including estrogen receptor α (ERα), progesterone receptor (PR), epidermal growth factor receptor (EGFR), insulin-like growth factor-1 receptor (IGF-1R), human epidermal growth factor receptor 3 (HER3, ERBB3), and G-protein coupled estrogen receptor (GPER) (Table 3, Tables S1 and S2). The binding affinities of the selected compounds ranged from −8.7 to −4.2 kcal/mol, indicating varying degrees of interaction strength. Among all tested compounds, Benzoxazinone glucoside exhibited the highest binding affinity, particularly against EGFR (−8.7 kcal/mol), followed by strong interactions with PR (−7.9 kcal/mol) and HER3 (−7.6 kcal/mol), suggesting its potential to modulate these signaling pathways. Compounds such as p-coumaroylagmatine, macamide B, and pipercitine also demonstrated notable binding affinities across multiple receptors, indicating their possible pharmacological relevance. In the case of ERα, pipercitine and p-coumaroylagmatine showed the highest binding affinity (−7.4 kcal/mol), primarily stabilized through hydrophobic interactions with key residues such as LEU346, ALA350, LEU387, and LEU525 (Table S4). Additionally, hydrogen bond formation with residues like GLU353 and THR347 contributed to enhanced binding stability, particularly in p-coumaroylagmatine. The presence of π-alkyl and π–π interactions with residues such as PHE404 and TYR537 further supports strong ligand accommodation within the receptor binding pocket. For the progesterone receptor, Benzoxazinone glucoside demonstrated the highest binding affinity (−7.9 kcal/mol), forming multiple hydrogen bonds with residues including VAL698, GLN815, ASP697, and ARG766 (Table S4). These interactions were further supported by electrostatic (π-cation) and hydrophobic interactions, indicating a stable ligand–receptor complex. Similarly, in IGF-1R, Benzoxazinone glucoside exhibited strong binding (−7.2 kcal/mol), predominantly mediated through hydrogen bonding with residues such as ARG104, ASN105, and ARG222, highlighting the importance of polar interactions in stabilizing the complex. The interaction analysis with EGFR revealed that Benzoxazinone glucoside possesses the strongest binding affinity among all compounds, forming multiple hydrogen bonds with SER196, ARG220, and SER205, along with hydrophobic and π–π interactions involving HIS209. This strong interaction profile suggests its potential may modulate the EGFR-mediated signaling pathways. In HER3, p-coumaroylagmatine and Benzoxazinone glucoside displayed high binding affinities (−7.7 and −7.6 kcal/mol, respectively), supported by hydrogen bonding with residues such as THR768, ASN820, and ASP833, along with hydrophobic interactions involving VAL704 and LEU822. Furthermore, p-coumaroylagmatine exhibited the highest binding affinity toward mPRα (−7.8 kcal/mol), forming stable hydrogen bonds with SER121, HIS279, and SER210, in addition to hydrophobic interactions with aromatic residues such as PHE248 and PHE278. In the case of GPER, Benzoxazinone glucoside showed the strongest binding (−6.7 kcal/mol), stabilized by hydrogen bonding with TYR175, ARG191, and ASN171, along with π–π stacking interactions that enhance ligand stability within the receptor binding site.

### 2.9. Molecular Docking and Interaction Studies of Chemical Compounds at Negative Ion Mode

The molecular docking analysis of phytoconstituents identified in the negative ion mode revealed significant binding interactions with multiple therapeutic targets, including Estrogen Receptor α (ERα), Progesterone receptor, IGF-1R, EGFR, HER3, mPRα, and GPER (Table 4 and Table S3). Among the evaluated compounds, quercetin 3-(2-caffeoylglucuronoside) and (−)-epicatechin 3′-O-glucuronide consistently demonstrated strong binding affinities across most targets, indicating their potential as key bioactive constituents. These compounds exhibited binding energies ranging from −8.2 to −10.2 kcal/mol, suggesting stable ligand–receptor complex formation compared to other identified metabolites. In the case of Estrogen Receptor α (ERα), both quercetin 3-(2-caffeoylglucuronoside) and (−)-epicatechin 3′-O-glucuronide showed the highest binding affinity (−8.2 kcal/mol). The interaction profile revealed multiple conventional hydrogen bonds with key amino acid residues such as TRP393, GLN441, HIS398, ASN439, and ARG394, along with electrostatic and hydrophobic interactions including π-alkyl and π–π stacking (Table S5). These interactions indicate strong stabilization within the active binding pocket of ERα. In contrast, fatty acid derivatives such as 8S-HODE and 3-keto stearic acid displayed comparatively weaker binding energies and were predominantly stabilized by hydrophobic interactions. For the Progesterone receptor, quercetin 3-(2-caffeoylglucuronoside) exhibited the strongest binding affinity (−9.9 kcal/mol), followed by (−)-epicatechin 3′-O-glucuronide (−9.5 kcal/mol). These ligands formed multiple hydrogen bonds with residues such as ARG766, CYS891, SER898, and GLN815, along with electrostatic interactions like π-cation and hydrophobic contacts (Table S5). The presence of both hydrogen bonding and hydrophobic interactions suggests a highly stable and specific binding conformation. Glucotropaeolin also showed good affinity (−8.0 kcal/mol), whereas other compounds demonstrated moderate to weak interactions. Similarly, in IGF-1R docking, (−)-epicatechin 3′-O-glucuronide showed the highest binding affinity (−8.4 kcal/mol), followed closely by quercetin 3-(2-caffeoylglucuronoside) (−8.1 kcal/mol). These compounds formed multiple hydrogen bonds with residues such as ARG104, ASN105, and ARG222, along with hydrophobic interactions involving LEU129 and MET184. Such interactions highlight their potential to modulate receptor activity effectively. Glucotropaeolin exhibited moderate binding affinity, while lipid-derived compounds displayed weaker binding patterns. In the case of EGFR, both quercetin 3-(2-caffeoylglucuronoside) and (−)-epicatechin 3′-O-glucuronide demonstrated strong binding affinities (−9.5 kcal/mol). Their interaction patterns included extensive hydrogen bonding with residues such as SER340, CYS287, GLU376, and ARG285, along with electrostatic and hydrophobic interactions. These findings suggest their potential role in modulating EGFR-mediated signaling pathways. Glucotropaeolin also showed appreciable binding (−8.1 kcal/mol), whereas other compounds showed comparatively lower affinities. For HER3 (ERBB3), quercetin 3-(2-caffeoylglucuronoside) exhibited a binding affinity of −9.8 kcal/mol, forming multiple hydrogen bonds with residues such as CYS721, SER698, ARG819, and ASN820, along with π-anion and hydrophobic interactions. (−)-Epicatechin 3′-O-glucuronide also demonstrated strong binding (−8.8 kcal/mol), supported by extensive hydrogen bonding networks. These interactions indicate a strong binding orientation within the receptor pocket, contributing to the stability of the ligand–protein complex. The docking results with membrane progesterone receptor alpha (mPRα) revealed the highest binding affinity for quercetin 3-(2-caffeoylglucuronoside) (−10.2 kcal/mol), indicating it as the most potent ligand among all studied compounds. It formed multiple hydrogen bonds with residues such as ASN192, LYS193, GLN206, and HIS279, along with electrostatic and hydrophobic interactions, including π–π stacking. (−)-Epicatechin 3′-O-glucuronide also showed strong binding (−8.8 kcal/mol), whereas glucotropaeolin exhibited moderate interaction strength. Finally, in GPER (GPR30), quercetin 3-(2-caffeoylglucuronoside) again demonstrated the highest binding affinity (−7.8 kcal/mol), followed by (−)-epicatechin 3′-O-glucuronide (−7.7 kcal/mol). The interaction profile included multiple hydrogen bonds with residues such as GLN39, TYR228, and GLU42, along with electrostatic and hydrophobic interactions. Other compounds exhibited relatively weaker binding affinities and fewer stabilizing interactions.

### 2.10. Molecular Docking and Interaction Analysis of Reference Drug Tamoxifen

To validate the docking protocol and provide a benchmark for comparison, the reference anti-estrogen drug Tamoxifen (4-hydroxytamoxifen) was docked against seven breast cancer–related molecular targets. The binding affinity values ranged from −6.8 to −7.9 kcal/mol, indicating favorable interactions with all selected receptors (Table S5). Among the evaluated targets, PR (−7.9 kcal/mol), mPRα (−7.9 kcal/mol), followed by ERα (−7.7 kcal/mol) and HER3 (−7.5 kcal/mol), displayed the strongest binding affinities, suggesting high receptor compatibility. The lowest affinity was observed for EGFR (−6.8 kcal/mol). Detailed interaction analyses revealed that hydrophobic contacts, hydrogen bonds, and electrostatic interactions played important roles in stabilizing the ligand–protein complexes. These findings are consistent with the known pharmacological behavior of tamoxifen and establish a reliable reference framework for comparing the docking performance of newly identified phytochemicals or lead compounds against these breast cancer therapeutic targets. These results validate the docking protocol and provide a benchmark for evaluating the therapeutic potential of the screened phytocompounds.

### 2.11. Physicochemical Properties of S. persica Root Phytoconstituents at Positive Ion Mode

The physicochemical profiling of phytoconstituents identified from *S. persica* root using HRLC-MS/MS provides important insights into their drug-likeness, bioavailability, and potential biological activity (Table 5). Overall, the molecular weight (MW) of the identified compounds ranges from 136.23 to 403.34 g/mol, with most compounds falling within the acceptable range (<500 g/mol) suggested by Lipinski’s rule of five. This indicates that the majority of phytoconstituents possess favorable characteristics for oral bioavailability. Compounds such as (+)-alpha-pinene and benzyl isothiocyanate exhibit low molecular weights, suggesting higher permeability, whereas benzoxazinone glucoside represents the upper limit but still remains within acceptable bounds. The number of heavy atoms and aromatic heavy atoms varies significantly, reflecting structural diversity. Aromaticity is particularly notable in compounds like p-coumaroylagmatine, 5-methoxydimethyltryptamine, and benzyl isothiocyanate, which may contribute to enhanced binding interactions through π–π stacking and hydrophobic interactions during molecular docking. Fraction Csp^3^ values indicate molecular complexity and saturation. Compounds such as medicanine, europine, and N-(14-methylhexadecanoyl) pyrrolidine show higher Csp^3^ fractions (>0.8), suggesting better three-dimensional conformations and potentially improved pharmacokinetic properties. In contrast, lower values in aromatic compounds reflect planar structures, which may influence target binding differently. Rotatable bonds, an indicator of molecular flexibility, range from 0 to 17. Highly flexible molecules such as macamide B and pipercitine may exhibit better adaptability within protein binding sites but could also suffer from reduced bioavailability due to entropy penalties. Conversely, rigid molecules like (+)-alpha-pinene may show limited conformational adaptability but enhanced membrane permeability. Hydrogen bond acceptors (HBA) and donors (HBD) are critical for ligand–protein interactions. Most compounds fall within acceptable limits (HBA ≤ 10 and HBD ≤ 5), supporting their drug-like nature. However, compounds such as benzoxazinone glucoside and asparaginyl-cysteine show higher polarity with increased HBA/HBD counts, which may enhance solubility but could reduce membrane permeability. Topological polar surface area (TPSA) values further support this observation. Compounds with TPSA below 140 Å^2^, such as medicanine, europine, and macamide B, are likely to exhibit good intestinal absorption. In contrast, highly polar compounds like asparaginyl-cysteine (TPSA: 174.31 Å^2^) may have limited permeability but could be beneficial for specific biological targets requiring high polarity. Molar refractivity (MR), reflecting molecular volume and polarizability, ranges from 40.58 to 117.1, indicating moderate to high binding potential across the compounds. Higher MR values, as seen in pipercitine and macamide B, suggest stronger van der Waals interactions in receptor binding. Notably, non-polar compounds such as (+)-alpha-pinene and N-(14-methylhexadecanoyl) pyrrolidine exhibit zero or very low TPSA and hydrogen bonding capacity, indicating high lipophilicity and potential for membrane permeability, but possibly limited aqueous solubility.

### 2.12. Physicochemical Properties of S. persica Root Phytoconstituents at Negative Ion Mode

The physicochemical profiling of phytoconstituents identified from *S. persica* root (HRLC-MS/MS, negative ion mode) revealed substantial diversity in molecular size, polarity, and structural flexibility (Table 6). The MW of the compounds ranged from 149.21 Da (benzyl isothiocyanate) to 640.5 Da (quercetin 3-(2-caffeoylglucuronoside)), indicating the presence of both low molecular weight bioactive and larger polyphenolic conjugates. Notably, glucotropaeolin and flavonoid glycosides such as quercetin derivatives and epicatechin glucuronide exhibited relatively high molecular weights (>400 Da), which may influence their permeability and bioavailability. Analysis of heavy atoms and aromaticity demonstrated that flavonoid-based compounds possessed a higher number of aromatic heavy atoms (12–22), reflecting their polyphenolic nature. In contrast, fatty acid derivatives such as 8S-HODE, 16-hydroxy hexadecanoic acid, and 3-keto stearic acid showed no aromaticity, consistent with their aliphatic structures. Fraction Csp^3^ values further supported this distinction, with fatty acids exhibiting higher saturation (0.72–0.94), whereas flavonoids showed lower values (0.17–0.38), indicating greater structural rigidity and aromatic character. Rotatable bond analysis revealed significant variability, with fatty acid derivatives showing the highest flexibility (14–16 rotatable bonds), while flavonoid conjugates and glucotropaeolin exhibited moderate flexibility (4–8 rotatable bonds). Benzyl isothiocyanate displayed minimal flexibility with only 2 rotatable bonds, suggesting a more rigid structure. Hydrogen bonding capacity varied considerably across the compounds. Flavonoid glycosides demonstrated high hydrogen bond acceptors (12–16) and donors (8–9), contributing to increased polarity and potential for strong intermolecular interactions. In contrast, benzyl isothiocyanate showed minimal hydrogen bonding capability (1 acceptor, 0 donors), indicating relatively low polarity. Fatty acids exhibited moderate hydrogen bonding potential (3 acceptors, 1–2 donors). Topological polar surface area (TPSA) ranged from 44.45 Å^2^ to 274.11 Å^2^. Highly polar compounds such as quercetin 3-(2-caffeoylglucuronoside) (274.11 Å^2^) and glucotropaeolin (199.79 Å^2^) may have limited membrane permeability, whereas benzyl isothiocyanate (44.45 Å^2^) falls within an optimal range for passive diffusion. Fatty acids exhibited moderate TPSA values (~54–57 Å^2^), suggesting balanced polarity and permeability. Molar refractivity (MR), indicative of molecular volume and polarizability, ranged from 45.38 to 154.16, with higher values observed for larger polyphenolic compounds. Overall, the dataset highlights a clear distinction between highly polar, polyphenolic glycosides with lower drug-likeness potential (due to high TPSA and molecular weight) and smaller or moderately polar compounds such as benzyl isothiocyanate and fatty acid derivatives, which may exhibit better pharmacokinetic properties. These findings provide a foundational basis for further in silico ADMET and molecular docking studies.

### 2.13. Lipophilicity Properties of Salvadora persica Root Phytoconstituents (Positive and Negative Ion Modes)

The lipophilicity profiling of *S. persica* root metabolites, assessed in both ionization modes, demonstrated a wide distribution of consensus Log P values (−2.09 to 6.64), reflecting considerable chemical heterogeneity with direct implications for absorption and distribution (Tables S8 and S9). Highly polar constituents, such as glucotropaeolin and (−)-epicatechin 3′-O-glucuronide, exhibited negative Log P values, indicating strong aqueous solubility but relatively poor membrane permeability due to their glycosylated structures. In contrast, compounds with intermediate lipophilicity (Log P ~1–3), particularly several flavonoid derivatives, showed a more favorable balance between solubility and permeability, suggesting better suitability for oral bioavailability. More hydrophobic molecules, including benzyl isothiocyanate, (+)-α-pinene, and long-chain fatty acid derivatives like 16-hydroxy hexadecanoic acid and 3-keto stearic acid, displayed higher Log P values, which may enhance membrane diffusion but could limit solubility and increase the likelihood of bioaccumulation at elevated levels. Collectively, this broad lipophilicity spectrum underscores the presence of structurally diverse metabolites with complementary pharmacokinetic characteristics, supporting their potential utility as multi-target bioactive candidates in drug discovery and computational studies.

### 2.14. Water Solubility Properties of Salvadora persica Root Phytoconstituents (Positive and Negative Ion Modes)

The estimated aqueous solubility of *S. persica* root constituents, assessed under both positive and negative ionization conditions, shows a wide range of behavior governed by differences in molecular size, polarity, and lipophilicity (Tables S11 and S12). Strongly polar compounds, including glucotropaeolin and (−)-epicatechin 3′-O-glucuronide, were consistently predicted to be highly soluble across ESOL, Ali, and Silicos-IT models, indicating good dissolution capacity but potentially reduced membrane permeability. In contrast, compounds with moderate lipophilicity, such as benzyl isothiocyanate and selected fatty acid derivatives (e.g., 8S-HODE), displayed intermediate solubility, suggesting a more balanced profile between aqueous solubility and membrane transport. More structurally complex polyphenols, including quercetin 3-(2-caffeoylglucuronoside), exhibited comparatively lower solubility, likely due to their higher molecular weight and increased structural complexity.

### 2.15. Pharmacokinetic Properties of S. persica Root Phytoconstituents at Positive Ion Mode

Most *S. persica* phytoconstituents demonstrate high gastrointestinal absorption, indicating favorable oral bioavailability, while a few (e.g., macamide B and benzoxazinone glucoside) show lower absorption due to extreme polarity or lipophilicity. Only select compounds, including 5-methoxydimethyltryptamine, (+)-α-pinene, and benzyl isothiocyanate, are predicted to cross the blood–brain barrier, suggesting limited but potential CNS activity, whereas most remain peripherally active (Table 7). A small number (e.g., leucyl-histidine, europine, pipercitine) are identified as P-glycoprotein substrates, which may reduce intracellular accumulation. The majority exhibit minimal inhibition of key CYP450 enzymes, implying a low risk of drug–drug interactions, although exceptions such as macamide B warrant attention. Skin permeability varies, with more lipophilic compounds showing better transdermal potential. Collectively, the ADME profile supports their promise as pharmacokinetically viable therapeutic candidates.

### 2.16. Pharmacokinetic Properties of S. persica Root Phytoconstituents at Negative Ion Mode

As shown in Table 8, GI absorption analysis revealed a clear distinction between compound classes. Glucotropaeolin, quercetin 3-(2-caffeoylglucuronoside), and (−)-epicatechin 3′-O-glucuronide exhibited low GI absorption, which may be attributed to their high polarity, large molecular size, and elevated topological polar surface area (TPSA). In contrast, benzyl isothiocyanate, 8S-HODE, 16-hydroxy hexadecanoic acid, and 3-keto stearic acid demonstrated high GI absorption, suggesting better oral bioavailability potential. BBB permeability results indicated that only the more lipophilic compounds—benzyl isothiocyanate and the fatty acid derivatives (8S-HODE, 16-hydroxy hexadecanoic acid, and 3-keto stearic acid)—are capable of crossing the blood–brain barrier. Conversely, polar flavonoid glycosides and glucotropaeolin were predicted to be non-permeant, limiting their central nervous system (CNS) exposure. Regarding efflux transport, glucotropaeolin was identified as a P-gp substrate, suggesting possible active efflux that could further reduce its intracellular accumulation and bioavailability. All other compounds were predicted to be non-substrates, indicating a lower likelihood of P-gp-mediated efflux. Cytochrome P450 inhibition profiling showed minimal interaction for most compounds, which is favorable in terms of reduced risk of drug–drug interactions. Notably, 16-hydroxy hexadecanoic acid was predicted to inhibit CYP2D6, while 3-keto stearic acid showed inhibitory activity against CYP1A2. The remaining compounds did not exhibit inhibitory effects on major CYP isoforms (CYP1A2, CYP2C19, CYP2C9, CYP2D6, and CYP3A4), suggesting a generally safe metabolic profile. Skin permeability (log Kp) values ranged from −10 to −3.17 cm/s. Highly negative values observed for glucuronide derivatives (e.g., −10 for epicatechin glucuronide) indicate poor skin permeation, consistent with their hydrophilic nature. In contrast, fatty acids and benzyl isothiocyanate showed less negative log Kp values (−3.17 to −4.97), reflecting relatively better dermal permeability.

### 2.17. Drug-Likeness Properties of S. persica Root Phytoconstituents (Positive and Negative Ion Modes)

The evaluation of drug-likeness for *S. persica* root phytoconstituents, analyzed in both positive and negative ion modes using Lipinski, Ghose, Veber, Egan, and Muegge criteria, demonstrated considerable variation influenced by molecular structure (Tables S15 and S16). Most low to moderately sized compounds adhered to Lipinski’s rule of five, suggesting good potential for oral drug development, although a few molecules, such as (+)-α-pinene and macamide B, displayed minor exceptions related to lipophilicity or size. The majority of compounds also satisfied Veber and Egan parameters, indicating suitable flexibility and polar surface area for efficient absorption. In contrast, structurally complex and highly polar constituents, particularly flavonoid glycosides like quercetin 3-(2-caffeoylglucuronoside), showed multiple deviations across these rules, largely due to their higher molecular weight and increased polarity.

### 2.18. Medicinal Chemistry Properties of S. persica Root Phytoconstituents (Positive and Negative Ion Modes)

The medicinal chemistry profiling of *S. persica* root–derived phytoconstituents, evaluated across both ionization modes, indicates a generally favorable landscape for drug development (Tables S18 and S19). The majority of compounds showed no PAINS alerts, suggesting minimal risk of false-positive bioactivity and supporting the credibility of their pharmacological potential; only quercetin 3-(2-caffeoylglucuronoside) displayed a minor alert. Structural liability assessment revealed relatively few Brenk alerts overall, although compounds such as glucotropaeolin (multiple alerts) and benzyl isothiocyanate or 8S-HODE (moderate alerts) may warrant further optimization to address possible reactivity or pharmacokinetic limitations. Lead-likeness evaluation indicated only minor deviations (typically one or two violations), with certain molecules, including benzyl isothiocyanate, exhibiting more favorable profiles. Synthetic accessibility analysis showed that most constituents fall within an easily to moderately synthesizable range (SA ~1.6–5.0), supporting their feasibility for large-scale production, whereas structurally complex flavonoids presented higher scores, implying greater synthetic effort. Collectively, these results underscore the promise of *S. persica* phytochemicals as drug candidates while highlighting a few compounds that may benefit from targeted structural refinement.

### 2.19. Toxicity Potential Analysis of S. persica Root Phytoconstituents at Positive Ion Mode

The toxicity potential of *S. persica* root phytoconstituents, predicted using Osiris DataWarrior, indicates that most compounds exhibit favorable safety profiles with low risks of mutagenicity, tumorigenicity, irritancy, and reproductive toxicity (Table 9). Several compounds, including medicanine, 3β,6β-dihydroxynortropane, and p-coumaroylagmatine, showed “green” indicators across all endpoints, suggesting good therapeutic safety. However, europine and benzyl isothiocyanate displayed mutagenicity alerts, while benzoxazinone glucoside and benzyl isothiocyanate indicated moderate tumorigenic potential. (+)-α-Pinene showed possible irritant effects, and benzyl isothiocyanate also raised concerns regarding reproductive toxicity. Drug-likeness scores varied widely, with compounds such as 5-methoxydimethyltryptamine and 3β,6β-dihydroxynortropane demonstrating favorable profiles, whereas peptide-like molecules showed lower scores.

### 2.20. Toxicity Potential Analysis of S. persica Root Phytoconstituents at Negative Ion Mode

The toxicity potential analysis of phytoconstituents identified from *S. persica* L. root in negative ion mode revealed varied safety profiles based on mutagenicity, tumorigenicity, irritant effects, reproductive toxicity, and drug-likeness parameters. Most of the compounds demonstrated favorable toxicity profiles, indicating their relative safety and suitability for further pharmacological investigations (Table 10). Among the analyzed compounds, quercetin 3-(2-caffeoylglucuronoside) and (−)-epicatechin 3′-O-glucuronide exhibited the most promising safety profiles, as they were predicted to be non-mutagenic, non-tumorigenic, non-irritant, and free from reproductive toxicity (all green indicators). Their relatively acceptable drug-likeness scores (−1.68 and −0.39, respectively) further support their potential as drug candidates. Similarly, glucotropaeolin showed a largely safe profile with green indicators for mutagenicity, tumorigenicity, and irritancy; however, it exhibited a red flag for reproductive toxicity, suggesting a need for cautious evaluation in reproductive-related studies. In contrast, benzyl isothiocyanate displayed notable toxicity concerns, as it was predicted to be mutagenic (red) and showed moderate risks for tumorigenicity and reproductive effects (orange), although it was non-irritant. Its poor drug-likeness score (−4.55) further indicates limitations in its therapeutic applicability. Fatty acid derivatives such as 8S-HODE, 16-hydroxy hexadecanoic acid, and 3-keto stearic acid were generally non-toxic across most parameters, although 16-hydroxy hexadecanoic acid showed irritant potential (red). Despite their low toxicity, these compounds exhibited very poor drug-likeness scores (ranging from −24.6 to −27.6), which may limit their drug development potential due to unfavorable pharmacokinetic properties.

### 2.21. ADMET Profiling of the Reference Drug Tamoxifen: Physicochemical, Pharmacokinetic, and Toxicological Assessment

The in silico ADMET analysis demonstrated that tamoxifen possesses favorable physicochemical and pharmacokinetic characteristics consistent with its established clinical use as an orally active anticancer drug. The compound exhibited suitable molecular properties, high lipophilicity, and strong membrane permeability, supporting efficient receptor interaction and pharmacological activity. Although tamoxifen showed poor aqueous solubility, its favorable permeability and distribution properties compensate for this limitation. Drug-likeness evaluation indicated acceptable pharmaceutical attributes with a moderate bioavailability score despite minor rule-based violations. Furthermore, medicinal chemistry assessment revealed good synthetic accessibility and minimal risk of assay interference, while toxicity prediction suggested low risks of mutagenicity, tumorigenicity, and irritation, with only a potential concern for reproductive effects. Collectively, these findings support the favorable ADMET profile of tamoxifen and are consistent with its long-standing therapeutic effectiveness in breast cancer treatment (Tables S6, S7, S10, S13, S14, S17, S20 and S21).

## 3. Discussion

Breast cancer continues to be the most frequently diagnosed malignancy among women worldwide and remains a significant contributor to cancer-related mortality despite advancements in therapeutic strategies. Recent global estimates indicate approximately 2.3 million new cases annually, highlighting the persistent burden of this disease (https://www.who.int/news-room/fact-sheets/detail/breast-cancer; accessed on 26 May 2026). The emergence of drug resistance, metastatic progression, and systemic toxicity associated with conventional chemotherapeutics underscores the urgent need for safer and more effective treatment alternatives [3]. In this regard, medicinal plants represent a valuable source of structurally diverse bioactive compounds with promising anticancer potential. The present study demonstrates that the ethanolic extract of *S. persica* root exerts significant cytotoxic effects against MCF-7 breast cancer cells through multiple mechanistic pathways. Unlike previous studies that primarily reported general biological activities or limited cytotoxic observations [7,9], this work integrates phytochemical profiling, mechanistic cell-based assays, molecular docking, and ADMET prediction to establish a broader framework for understanding the anticancer potential of *S. persica* root extract against breast cancer. The identification of flavonoid conjugates, glucosinolates, isothiocyanates, and other bioactive metabolites exhibiting favorable interactions with multiple breast cancer-related therapeutic targets, including ERα, PR, EGFR, HER3, IGF-1R, mPRα, and GPER, suggests that the observed biological activity may arise from a multi-target mode of action. Furthermore, the favorable pharmacokinetic and safety predictions obtained for several identified compounds support their potential as lead candidates for future drug development. The observed anti-proliferative activity appears to be mediated via induction of oxidative stress, disruption of mitochondrial integrity, activation of apoptosis, and interference with cell cycle progression. These biological effects were further corroborated by phytochemical profiling and molecular docking analyses, suggesting that *S. persica* root -derived compounds may modulate key signaling pathways involved in breast cancer development and progression. Preliminary cytotoxicity assessment indicated that the *S. persica* root extract produced a more pronounced inhibitory effect in MCF-7 cells than in MDA-MB-231 cells. Accordingly, MCF-7 cells were chosen for subsequent mechanistic analyses to obtain clearer and more reproducible biological responses. Moreover, MCF-7 cells are well-suited for studying ROS-mediated mitochondrial apoptosis and express key hormone receptors (ERα, PR, IGF-1R) that are relevant to the molecular docking targets identified in this study [10,11], thereby providing coherence between the experimental and computational findings and supporting the use of MCF-7 cells for in-depth mechanistic investigation.

HRLC-MS-based phytochemical analysis revealed a chemically diverse metabolite composition in *S. persica* root extract, encompassing alkaloids, phenolics, terpenoids, fatty acid derivatives, and peptide-like constituents. Such diversity is characteristic of medicinal plants and is often associated with broad-spectrum pharmacological activities. Earlier studies have also reported the presence of flavonoids, tannins, and sulfur-containing compounds in *S. persica*, which are known to possess antioxidant, anti-inflammatory, and antimicrobial properties [12]. Importantly, these classes of phytochemicals have been widely documented to exhibit anticancer effects by modulating intracellular signaling cascades and redox homeostasis [13,14]. The extract exhibited dose-dependent cytotoxicity against MCF-7 cells, with an IC_50_ value of 167 µg/mL, indicating moderate yet significant antiproliferative potential. Notably, minimal toxicity was observed in normal Vero cells, suggesting selective targeting of cancer cells. This selective cytotoxicity is a critical feature for potential therapeutic agents, as it minimizes damage to normal tissues and reduces adverse effects commonly associated with chemotherapy. Morphological alterations such as cell shrinkage, detachment, and chromatin condensation further confirmed the induction of apoptotic cell death. These observations were substantiated by AO/EtBr and Annexin V-FITC/PI staining, which demonstrated a significant increase in both early and late apoptotic cell populations in a concentration-dependent manner. Collectively, these findings indicate that *S. persica* root -induced cytotoxicity predominantly occurs via programmed cell death rather than necrosis.

A key mechanistic insight from this study is the substantial elevation of intracellular reactive oxygen species (ROS) following *S. persica* root treatment. ROS plays a complex role in cancer biology, functioning as both signaling molecules and mediators of cellular damage [15]. While moderate ROS levels may support tumor growth, excessive accumulation leads to oxidative stress, damaging essential biomolecules and triggering apoptosis [16]. The marked increase in ROS levels observed in treated MCF-7 cells suggests that *S. persica* root extract disrupts redox balance, thereby promoting oxidative stress-mediated cytotoxicity. This increase in ROS was closely associated with a significant loss of MMP, indicating activation of the intrinsic apoptotic pathway. Mitochondrial dysfunction is a hallmark event in apoptosis, leading to the release of pro-apoptotic factors such as cytochrome c and subsequent activation of caspase cascades [17]. The observed mitochondrial depolarization suggests that *S. persica* root phytoconstituents may directly impair mitochondrial function, thereby committing cells to apoptosis. Furthermore, cell cycle analysis revealed arrest at the S phase, indicating that *S. persica* root interferes with DNA synthesis and replication processes. Cell cycle arrest at this checkpoint prevents the proliferation of damaged cells and can lead to apoptotic elimination if repair mechanisms fail [18]. This suggests that *S. persica* root extract may modulate key regulatory proteins involved in cell cycle control, thereby inhibiting tumor growth and progression.

The molecular docking analysis provided additional mechanistic validation by demonstrating strong interactions between *S. persica* root-derived phytoconstituents and clinically relevant breast cancer targets. Notably, benzoxazinone glucoside exhibited high binding affinity toward several receptors, including G-protein-coupled estrogen receptor (GPER), insulin-like growth factor receptor (IGF-1R), progesterone receptor (PR), epidermal growth factor receptor (EGFR), HER3, and estrogen receptor alpha (ERα). This multi-target binding profile is particularly significant, as breast cancer progression is driven by the dysregulation of multiple signaling pathways. Inhibition of hormone receptors such as ERα and PR suggests potential efficacy in hormone-responsive breast cancer, while targeting EGFR, HER3, and IGF-1R may suppress proliferative and survival signaling pathways associated with aggressive tumor phenotypes and therapeutic resistance [19]. Additionally, interactions with GPER highlight the potential to modulate non-genomic estrogen signaling, which plays a role in tumor progression and endocrine resistance [20]. The ability of *S. persica* root phytoconstituents to simultaneously target multiple receptors suggests a synergistic therapeutic effect, which is a distinct advantage of plant-derived compounds over single-target synthetic drugs. Pharmacokinetic and drug-likeness predictions further support the therapeutic potential of these compounds. The pharmacokinetic analysis indicates that most *S. persica* phytoconstituents exhibit favorable ADME characteristics, including high gastrointestinal absorption and minimal CYP inhibition, supporting their suitability for oral use. Several metabolites demonstrated compliance with established criteria for oral bioavailability, indicating favorable absorption and distribution characteristics. Toxicity predictions suggested a generally safe profile for most compounds, particularly quercetin 3-(2-caffeoylglucuronoside) and (−)-epicatechin 3′-O-glucuronoside, although certain metabolites require cautious evaluation due to potential bioactivity-related risks. The identification of glucosinolate-derived compounds such as benzyl isothiocyanate is particularly noteworthy, as these molecules are well-known for their chemopreventive properties, including induction of detoxification enzymes, inhibition of cell proliferation, and promotion of apoptosis [21]. Thus, the findings of this study suggest that *S. persica* root extract exerts anticancer effects through a multifaceted mechanism involving oxidative stress induction, mitochondrial dysfunction, cell cycle arrest, and modulation of key oncogenic receptors. The presence of multiple bioactive compounds may further enhance therapeutic efficacy through synergistic interactions. Furthermore, to provide a comparative standard for the docking analysis, Tamoxifen was included as a reference anti-breast cancer drug. Several phytoconstituents identified from *S. persica* bark exhibited binding affinities comparable to or, in some cases, stronger than Tamoxifen against the selected therapeutic targets, suggesting their potential to interact effectively with proteins involved in breast cancer progression.

The present study has certain limitations that should be considered when interpreting the findings. Although some identified phytoconstituents exhibited predicted toxicity alerts, the crude *S. persica* root extract demonstrated moderate cytotoxicity against breast cancer cells while showing minimal toxicity toward normal Vero cells. Moreover, the induction of ROS generation, mitochondrial membrane depolarization, apoptosis, and cell-cycle arrest suggests that the observed anticancer activity cannot be attributed solely to non-specific toxicity. However, as the extract represents a complex mixture of phytochemicals, the individual contributions of beneficial and potentially harmful constituents remain unclear. Therefore, bioactivity-guided fractionation, isolation of active compounds, and detailed toxicological evaluations are required to establish their safety and therapeutic potential. Additionally, the anticancer activity was evaluated only in MCF-7 and MDA-MB-231 cells; although the latter represents the clinically aggressive triple-negative breast cancer subtype, further studies using additional breast cancer models and in vivo systems are needed to confirm the broader applicability of the observed mechanisms. Furthermore, while several phytoconstituents exhibited favorable docking scores comparable to the reference drug 4-hydroxytamoxifen, these findings remain predictive and require validation through molecular dynamics simulations, target-based biochemical assays, and in vivo studies to confirm their biological relevance.

## 4. Materials and Methods

### 4.1. Reagents and Chemicals

Sigma-Aldrich (St. Louis, MO, USA) provided Dulbecco’s Modified Eagle Medium/Nutrient Mixture F-12 (DMEM/F-12), penicillin–streptomycin solution, fetal bovine serum (FBS), 2′,7′-dichlorofluorescin diacetate (DCFH-DA), and Rhodamine-123. HiMedia Laboratories (India) provided the MTT reagent and 4′,6-diamidino-2-phenylindole (DAPI) stain. Annexin V-FITC apoptosis detection kit was brought from BioVision (Milpitas, CA, USA). Throughout the investigation, only analytical-grade chemicals and reagents were utilized.

### 4.2. Collection and Authentication of Plant Material

Fresh specimens of *S. persica* (Miswak), including roots, were collected from Maharaj Ganj, Raebareli district, Uttar Pradesh, India, in August 2025. The plant material was taxonomically authenticated, and a voucher specimen (Accession No. IU/PHAR/HRB/25/08) was deposited in the Herbarium of the Department of Pharmacognosy and Phytochemistry, Integral University, Lucknow, India, for future reference.

### 4.3. Preparation of Hydroethanolic Extract of Miswak Roots

The collected roots were separated, thoroughly rinsed with tap water followed by double-distilled water, and shade-dried for approximately two weeks. The dried material was then coarsely powdered using a mechanical grinder (Bajaj Rex, Mumbai, Maharashtra, India). Extraction was carried out by cold percolation using 75% ethanol at ambient temperature. The powdered material was immersed in the solvent and allowed to stand for 48 h, after which the extract was filtered using Whatman No. 1 filter paper (125 mm). This procedure was repeated multiple times to ensure exhaustive extraction. The combined filtrates were concentrated under reduced pressure at 45 °C using a rotary evaporator (Buchi Rotavapor R-205, Flawil, Switzerland). The semi-solid extract obtained was further concentrated on a water bath and stored in an airtight container at 4 °C. A single batch of hydroethanolic *S. persica* bark extract was used throughout all phytochemical and biological assays to ensure experimental consistency and reduce batch-to-batch variability.

### 4.4. Phytochemical Profiling by HR-LC–MS/MS Analysis

Using a UHPLC system in conjunction with an electrospray ionization quadrupole time-of-flight mass spectrometer (UHPLC-ESI-QTOF-MS; Agilent Technologies, Santa Clara, CA, USA), high-resolution liquid chromatography–mass spectrometry (HR-LC–MS/MS) was used to profile the extract’s metabolites. MassHunter LC/MS Data capture software (version B.06.01) was used for data capture and instrument control, whereas MassHunter Qualitative and Quantitative Analysis software (version B.07.00) was used for data processing. All samples were passed through a 0.2 μm nylon membrane filter before analysis. A gradient elution system consisting of (A) water with 0.1% formic acid and (B) acetonitrile with 0.1% formic acid was used to accomplish chromatographic separation on a Zorbax Eclipse C18 column (2.1 × 150 mm, 5 μm particle size). 0–20 min (95% A, 5% B), 20–25 min (5% A, 95% B), and 26–30 min (95% A, 5% B) were the settings for the gradient program. With a system pressure of about 1200 bar, the flow rate was kept at 0.2 mL/min. Both positive and negative electrospray ionization modalities were used for mass spectrometric detection. The extraction voltage, cone voltage, and capillary voltage were all set to 4 V, 30 V, and 3.25 kV in positive mode. At a flow rate of 900 L/h, nitrogen was utilized as the desolvation gas. The temperatures of the source and desolvation were kept at 120 °C and 550 °C, respectively. Mass spectra were obtained at a resolution of about 22,000 FWHM over a *m*/*z* range of 100–1200.

### 4.5. Cell Culture

Human breast adenocarcinoma cells (MCF-7), triple-negative breast cancer (MDA-MB-231), and normal kidney epithelial cells (Vero) were procured from NCCS, Pune, India. Cells were cultured in DMEM/F-12 (1:1) medium supplemented with 2 mM L-glutamine, 10% FBS, and 1% antibiotic–antimycotic solution. Cultures were maintained in T-25 cm^2^ flasks at 37 °C in a humidified atmosphere containing 5% CO_2_ using a CO_2_ incubator (Forma Steri-Cycle, Thermo Scientific, Waltham, MA, USA).

### 4.6. MTT Cytotoxicity Assay

The antiproliferative potential of *S. persica* root extract was evaluated in MCF-7, MDA-MB-231, and Vero cell lines using the MTT assay following a previously established protocol [22]. Cells were incubated in culture plate of 96-well (1 × 10^4^ cells/mL) and allowed to adhere overnight. The extract was dissolved in culture medium and serially diluted (25–300 µg/mL), followed by 24 h exposure. After treatment, MTT reagent (5 mg/mL) was added, and absorbance was recorded at 550 nm with a reference wavelength of 630 nm using a microplate reader (Thermo PW41, Waltham, MA, USA). IC_50_ values were calculated using linear regression curve analysis. Morphological alterations were examined under an inverted phase-contrast microscope (Nikon Eclipse TS100, Tokyo, Japan).

### 4.7. Trypan Blue Exclusion Assay

This assay directly distinguishes viable and non-viable cells based on membrane integrity and is independent of mitochondrial enzyme activity [23]. For this, both types of cells were seeded into 6-well plates and treated with a desired concentration for 24 h. After the treatment period, cells were harvested, and trypan blue solution (0.4%, Sigma-Aldrich, St. Louis, MO, USA) was used to stain the sample for 3–4 min at RT. Both live and dead cells were counted under an Inverted microscope. Percent cell viability was calculated based on the formula of (total live cells/total cells) × 100.

### 4.8. Staining Procedure of Acridine Orange/Ethidium Bromide (AO/EtBr)

Apoptotic changes were assessed using AO/EtBr dual staining as per the published study [24]. Following 24 h exposure to selected concentrations of the extract, cells were harvested and stained with AO and EtBr (2 µg/mL each). After incubation at 37 °C for 10 min, cells were washed with cold PBS and visualized using a fluorescence microscope (Zeiss AxioVert 135, Oberkochen, Baden-Württemberg, Germany) to distinguish viable, apoptotic, and necrotic populations.

### 4.9. Annexin V–FITC/PI Apoptosis Assay

Apoptosis was quantified using an Annexin V–FITC/PI staining kit and analyzed by flow cytometry (FACS Lyric, BD Biosciences, San Jose, CA, USA). Cells (1 × 10^6^ cells/mL) were treated with selected extract concentrations for 24 h, harvested, and resuspended in binding buffer. Before analysis, cells were stained with propidium iodide and Annexin V-FITC and allowed to sit at room temperature in the dark for 15 min.

### 4.10. Measurement of Intracellular ROS

Intracellular ROS levels were evaluated using DCFH-DA staining [22]. For qualitative analysis, cells were incubated with 10 µM DCFH-DA for 20 min, washed, and imaged under a fluorescence microscope. For quantitative assessment, treated and control cells were stained similarly and analyzed using flow cytometry to determine ROS-associated fluorescence intensity.

### 4.11. MMP (ΔΨm) Analysis

Changes in MMP were assessed using Rhodamine-123 staining [22]. Treated cells were incubated with 10 µM Rh-123 for 30 min in the dark, washed with PBS, and analyzed by flow cytometry. Fluorescence microscopy was also performed to visualize mitochondrial depolarization.

### 4.12. Cell Cycle Analysis

Propidium iodide staining was used to detect cell cycle distribution in accordance with a prior technique [25]. Cells were treated with extract concentrations (LD, MD, and HD) for 24 h, fixed in 70% chilled ethanol, and incubated with RNase A followed by PI staining (5 µg/mL). DNA content was analyzed using flow cytometry to determine cell cycle phase distribution.

### 4.13. Ligand Preparation

Three-dimensional structures of selected phytoconstituents were retrieved from the PubChem database in SDF format. Energy minimization was performed using Avogadro 1.2.0 with the MMFF94 force field. Ligands were further processed in AutoDockTools 1.5.7 and converted to PDBQT format using Open Babel 3.1.1 for docking studies [26].

### 4.14. Protein Preparation

Crystal structures of target proteins, including ERα (1R5K), PR (4OAR), IGF-1R (1IGR), EGFR (1IVO), HER3 (3KEX), mPRα (Q86WK9), and GPER (8XOG), were obtained from the Protein Data Bank. Protein structures were refined using BIOVIA Discovery Studio 2022 by eliminating water molecules, ligands, and heteroatoms according to a published study [27].

### 4.15. Molecular Docking (PyRx)

Docking simulations were performed using PyRx 0.8 with AutoDock Vina 1.1.2 as the scoring engine. Prepared ligands and proteins were imported, and grid boxes were defined around active sites. Docking was conducted with an exhaustiveness value of 8. Binding affinities were recorded, and top-ranked conformations were selected based on consensus scoring. Interaction profiles were visualized using BIOVIA Discovery Studio 2022 [28].

### 4.16. SwissADME Analysis

Pharmacokinetic and drug-likeness properties of the identified compounds were predicted using the SwissADME web server. Parameters analyzed included lipophilicity, solubility, molecular descriptors, gastrointestinal absorption, BBB permeability, CYP450 inhibition, and compliance with drug-likeness rules [29].

### 4.17. Toxicity Prediction

Osiris Property Explorer (https://openmolecules.org/datawarrior/; accessed on 10 February 2026)/DataWarrior 5.5.0 was used to assess toxicological profiles. Factors including reproductive toxicity, irritation, tumorigenicity, and mutagenicity, and drug-likeness scores were assessed using QSAR-based predictions [30].

### 4.18. Statistical Analysis

The mean ± SD of three separate experiments was used to express the experimental findings. Before statistical analysis, data distribution was assessed using the Shapiro–Wilk test. Owing to the limited number of replicates per treatment group (*n* = 3), normality was assessed using pooled experimental observations for each cell line. The analysis indicated no substantial departure from normality; therefore, parametric statistical methods were considered appropriate. One-way ANOVA and Dunnett’s multiple comparison test using GraphPad Prism v5.01 (San Diego, CA, USA) were used to establish statistical significance; *p* < 0.05 was deemed statistically significant.

## 5. Conclusions

This study provides strong scientific evidence supporting the therapeutic potential of *S. persica* root as a promising source of novel anti-breast cancer agents and lays a solid foundation for future translational oncology research. The present study demonstrates that *S. persica* root extract exerts potent anticancer effects against MCF-7 breast cancer cells through a combination of mechanisms involving ROS-mediated oxidative stress, mitochondrial dysfunction, apoptosis induction, and cell cycle arrest. The integration of phytochemical profiling, in vitro assays, and molecular docking analysis provides a comprehensive understanding of the molecular basis of its anticancer activity. However, further studies, including in vivo models, molecular pathway validation, and isolation of active compounds, are required to fully elucidate the therapeutic potential of *S. persica* in breast cancer treatment.

## Figures and Tables

**Table 1:** Identification of chemical compounds from *S. persica* root extract by HRLC-MS/MS at Positive ion mode, representing the retention time, score, chemical formula, exact mass, *m*/*z* value, and class of compounds.

S. No.	Phytochemicals	RT	Mass	Formula	PubChem CID	*m*/*z*	2D Structure	Class of Compound
1.	Medicanine	1.42	159.087	C_7_H_13_NO_3_	101409750	160.095	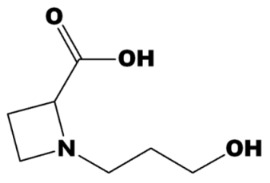	Alkaloids
2.	3beta,6beta Dihydroxynortropane	1.75	143.093	C_7_H_13_NO_2_	22297531	144.100	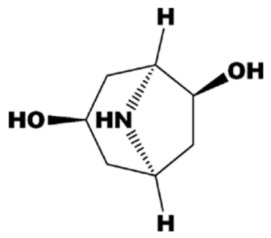	Alkaloids
3.	N-Acetyl-leucyl-leucin	6.06	286.185	C_14_H_26_N_2_O_4_	443129	287.192	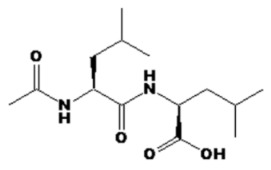	Small Peptides
4.	Leucyl-Histidine	7.40	268.153	C_12_H_20_N_4_O_3_	6992828	269.160	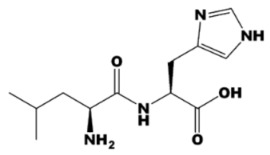	Dipeptide
5.	p-Coumaroylagmatine	9.53	276.158	C_14_H_20_N_4_O_2_	440362	277.165	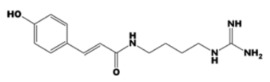	Phenylpropanoid-polyamine
6.	5- Methoxydimethyltryptamine	11.41	218.141	C_13_H_18_N_2_O	1832	241.130	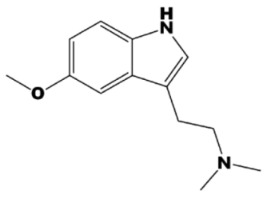	Indole alkaloid
7.	N(alpha)-gamma-L Glutamylhistamine	11.89	240.122	C_10_H_16_N_4_O_3_	440238	241.13	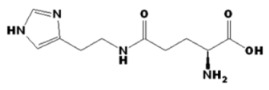	Amino-histamine conjugate
8.	Europine	12.69	329.184	C_16_H_27_NO_6_	5462451	330.191	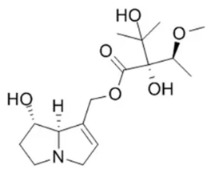	Pyrrolizidine alkaloid
9.	(+)-alpha-Pinene	13.77	136.123	C_10_H_16_	82227	159.113	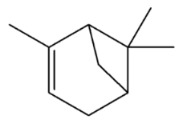	Monoterpene
10.	Benzyl isothiocyanate	15.28	149.028	C_8_H_7_NS	2346	294.065	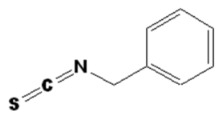	Isothiocyanate
11.	Asparaginyl-Cysteine	19.83	235.063	C_7_H_13_N_3_O_4_S	18218178	236.070	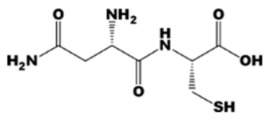	Amino acid conjugate
12.	Macamide B	22.83	345.300	C_23_H_39_NO	11198769	368.29	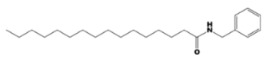	Fatty acid amide
13.	Benzoxazinone glucoside	23.77	403.112	C_16_H_21_NO_11_	77195081	404.119	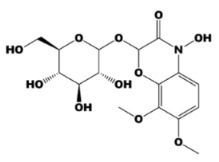	Glycosylated defense metabolite
14.	N-(14-Methylhexadecanoyl)pyrrolidine	25.01	323.316	C_21_H_41_NO	6430518	346.305	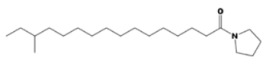	Fatty amide
15.	Pipercitine	25.37	349.331	C_23_H_43_NO	12575258	372.320	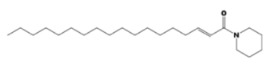	Piperamide

**Table 2:** Identification of chemical compounds from *S. persica* Root extract by HRLC-MS/MS at Negative ion mode, representing the retention time, score, chemical formula, exact mass, *m*/*z* value, and class of compounds.

S. No.	Compound Name	RT (min)	Mass (Da)	MF	Abundance	PubChem CID	*m*/*z*	2D Structure	Chemical Class
1.	Glucotropaeolin	3.65	409.04	C_14_H_19_NO_9_S_2_	465,977.25	9548605	408.04	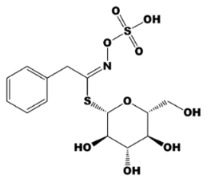	Glucosinolate
2.	Quercetin 3-(2-caffeoylglucuronoside)	4.82	640.11	C_30_H_24_O_16_	14,584.44	131753131	699.12	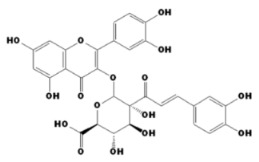	Flavonoid glycoside
3.	(−)-Epicatechin 3′-O-glucuronide	5.93	466.11	C_21_H_22_O_12_	128,354.55	76969982	511.11	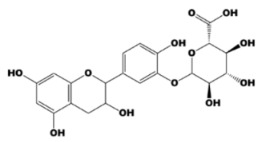	Flavonoid conjugate
4.	Benzyl isothiocyanate	10.71	149.0286	C_8_H_7_NS	570	2346	294.06	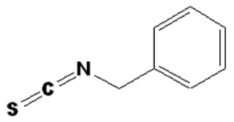	Isothiocyanate
5.	8S-HODE	17.31	296.23	C_18_H_32_O_3_	68,167.35	16061037	295.22	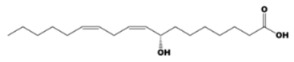	Oxidized fatty acid
6.	16-Hydroxy hexadecanoic acid	18.97	272.23	C_16_H_32_O_3_	353,409.25	10466	271.22	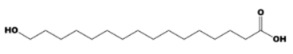	Hydroxy fatty acid
7.	3-Keto stearic acid	21.04	298.25	C_18_H_34_O_3_	168,273.86	5283005	297.24	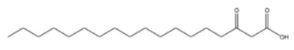	Fatty acid derivative

**Table 3:** Molecular docking and interaction studies of chemical compounds identified at Positive ion mode with therapeutic membrane receptors of breast cancer, viz. Estrogen Receptor α (ERα/ESR1; PDB ID: 1R5K), Progesterone receptor (PR; PDB ID: 4OAR), Insulin-Like Growth Factor-1 Receptor (IGF-1R; PDB ID: 1IGR), Epidermal Growth Factor Receptor (EGFR/ERBB1; PDB ID: 1IVO), Human Epidermal growth factor Receptor 3 (HER3; PDB ID: 3KEX), Membrane progesterone receptor alpha (mPRα; PDB identifier/UniProt entry Q86WK9) and G-Protein Coupled Estrogen Receptor (GPER/GPR30; PDB ID: 8XOG) through PyRx 1.1 docking tool.

S. No.	Compounds Name	Binding Affinity (kcal/mol)	3D Interaction	2D Interaction
Binding interaction with Estrogen Receptor α (ERα/ESR1)				
1.	Pipercitine	−7.4	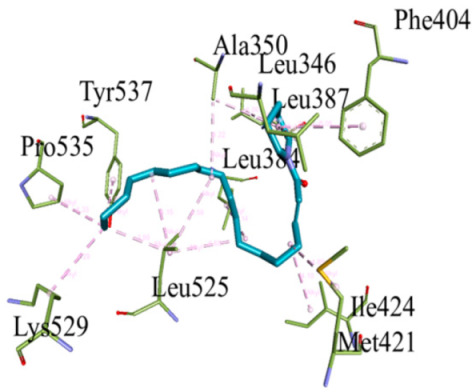	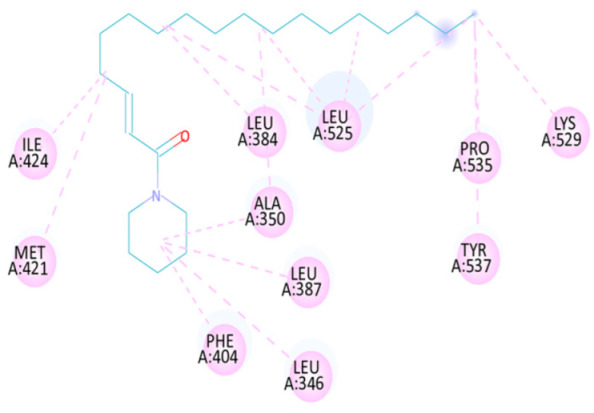
2.	p-Coumaroylagmatine	−7.4	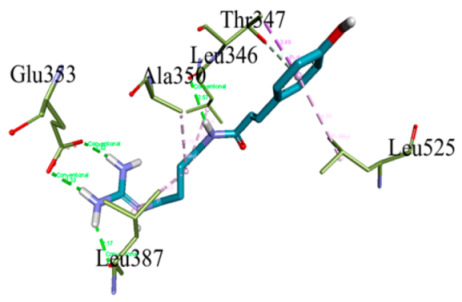	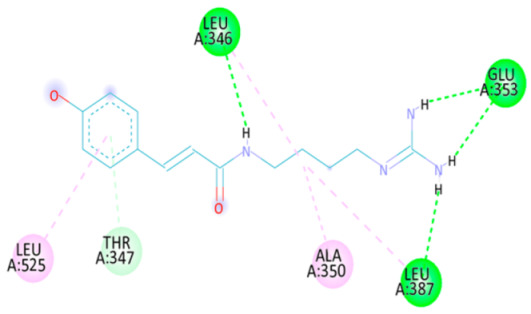
Binding interaction with Progesterone receptor				
3.	Benzoxazinone glucoside	−7.9	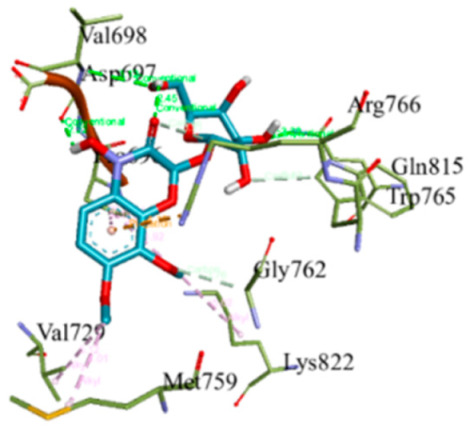	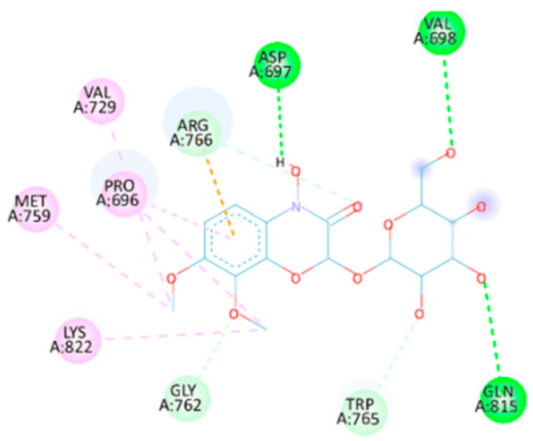
Binding interaction with Insulin-Like Growth Factor-1 Receptor (IGF-1R)				
4.	Benzoxazinone glucoside	−7.2	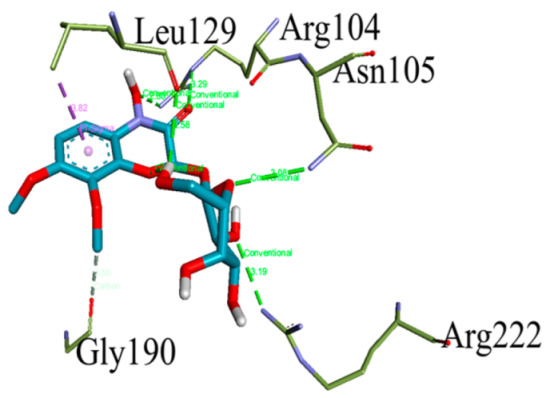	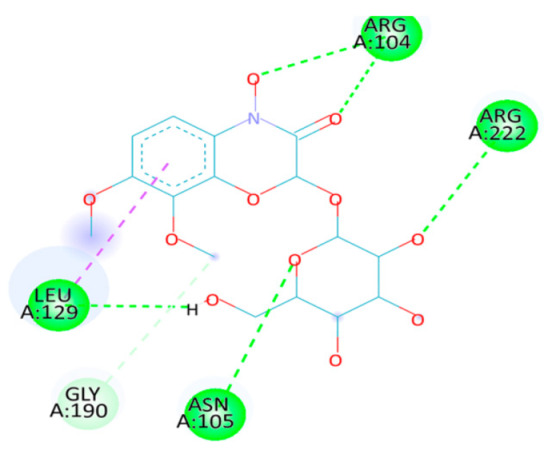
Binding interaction with Epidermal Growth Factor Receptor (EGFR/ERBB1)				
5.	Benzoxazinone glucoside	−8.7	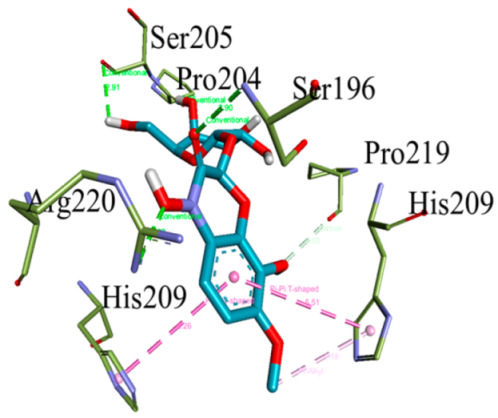	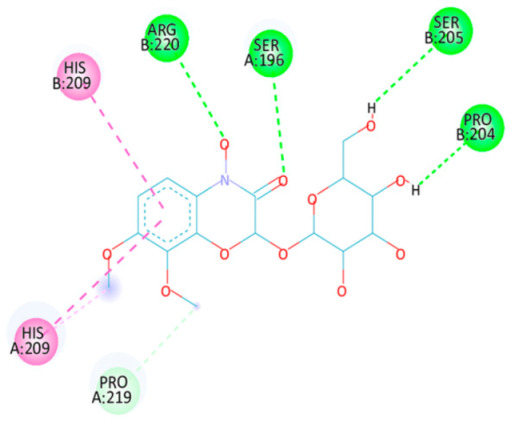
Binding interaction with human epidermal growth factor Receptor 3 (HER3)				
6.	p-Coumaroylagmatine	−7.7	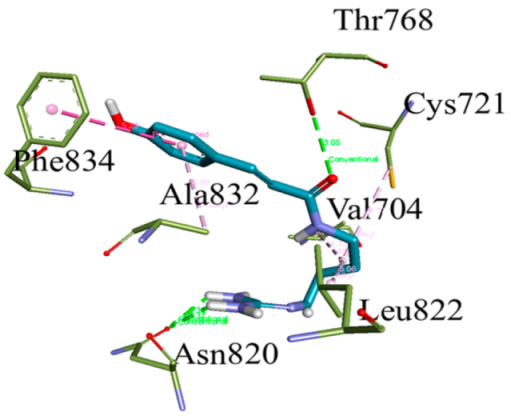	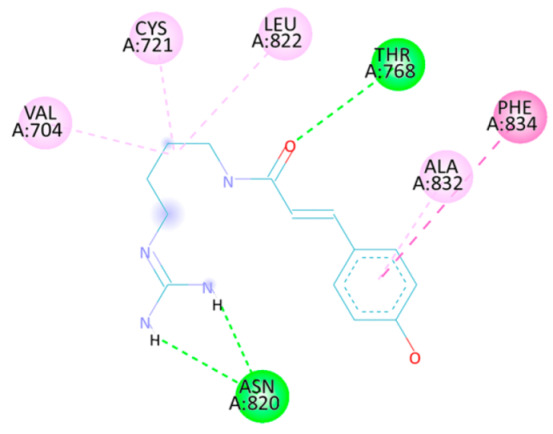
7.	Benzoxazinone glucoside	−7.6	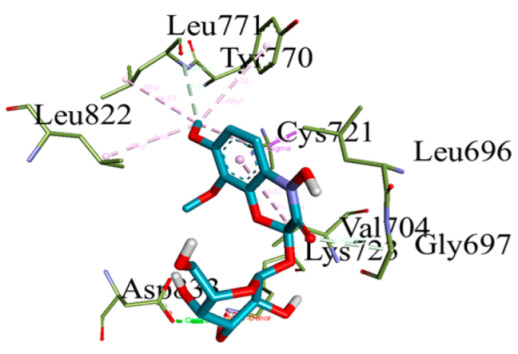	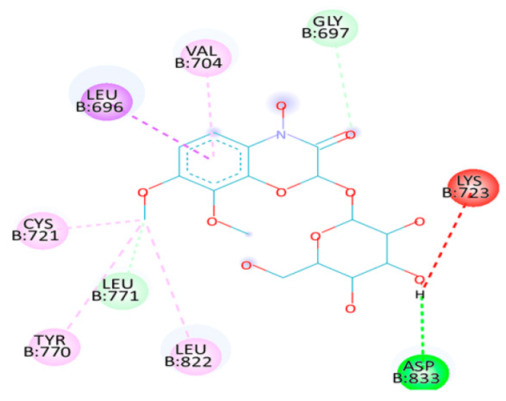
Binding interaction with Membrane progesterone receptor alpha (mPRα)				
8.	p-Coumaroylagmatine	−7.8	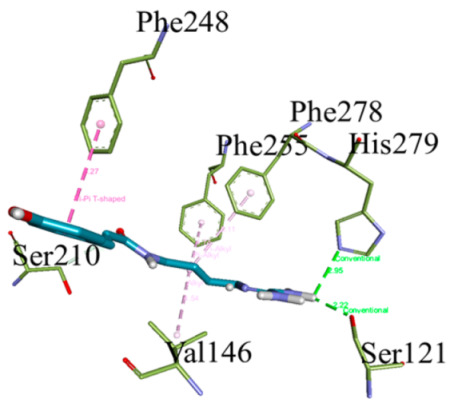	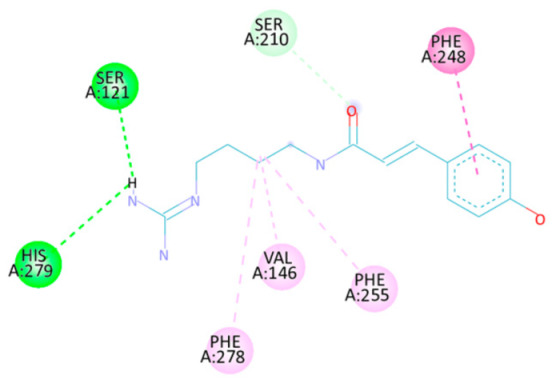
Binding interaction with G-Protein Coupled Estrogen Receptor (GPER/GPR30)				
9.	Benzoxazinone glucoside	−6.7	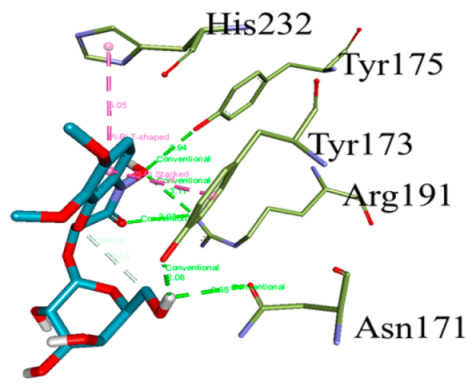	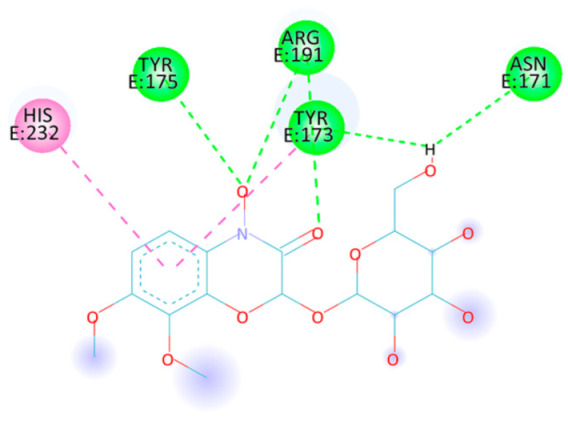

**Table 4:** Molecular docking and interaction studies of chemical compounds identified at the negative ion mode with membrane receptors of breast cancer, viz. Estrogen Receptor α (ERα; PDB ID: 1R5K), Progesterone receptor (PR; PDB ID: 4OAR), Insulin-Like Growth Factor-1 Receptor (IGF-1R; PDB ID: 1IGR), Epidermal Growth Factor Receptor (EGFR; PDB ID: 1IVO), Human Epidermal Growth Factor Receptor 3 (HER3; PDB ID: 3KEX), Membrane progesterone receptor alpha (mPRα; PDB identifier/UniProt entry Q86WK9), and G-Protein Coupled Estrogen Receptor (GPER; PDB ID: 8XOG) through the PyRx docking tool.

S. No.	Compounds Name	Binding Affinity (kcal/mol)	3D Interaction	2D Interaction
Binding interaction with Estrogen Receptor α (ERα/ESR1)				
1.	Quercetin 3-(2-caffeoylglucuronoside)	−8.2	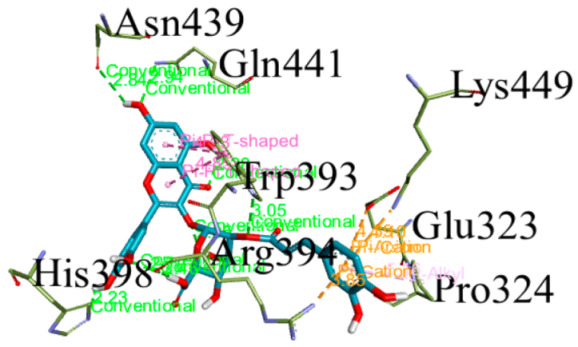	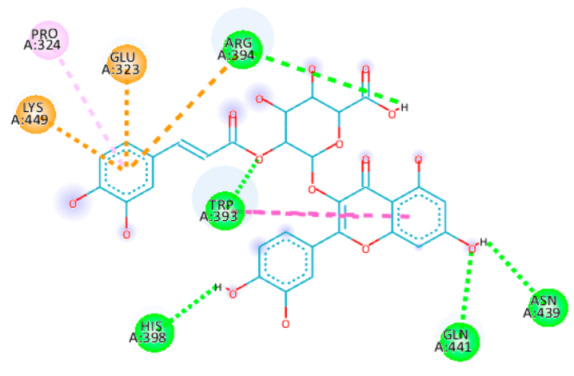
2.	(−)-Epicatechin 3′-O-glucuronide	−8.2	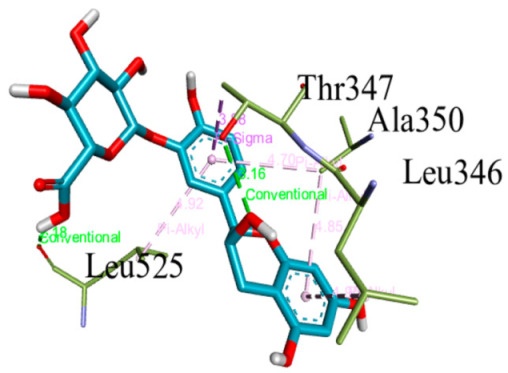	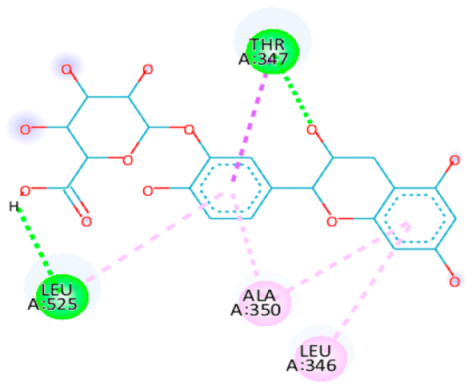
Binding interaction with Progesterone receptor				
3.	Quercetin 3-(2-caffeoylglucuronoside)	−9.9	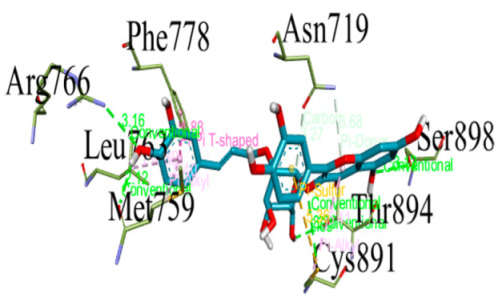	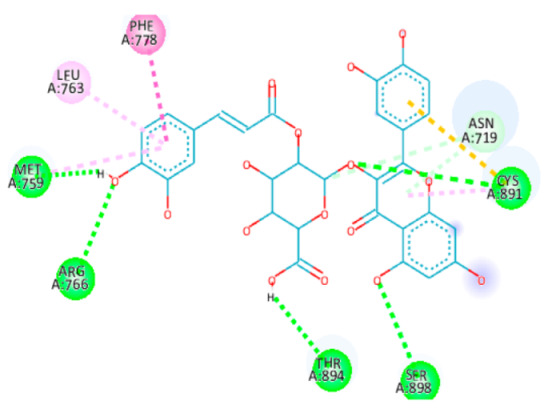
4.	(−)-Epicatechin 3′-O-glucuronide	−9.5	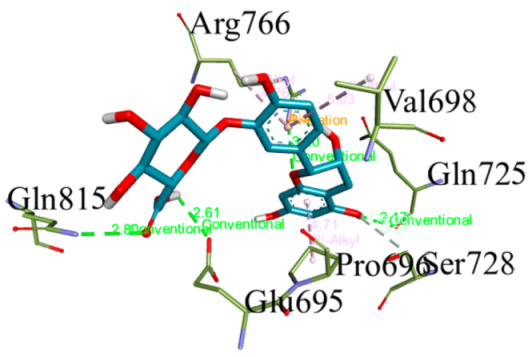	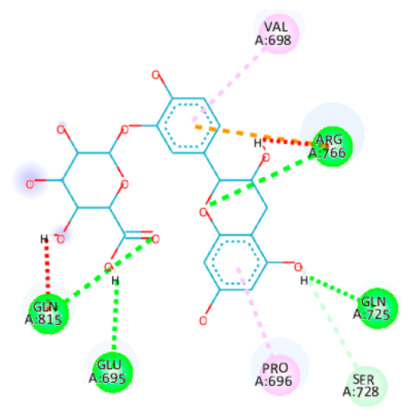
5.	Glucotropaeolin	−8	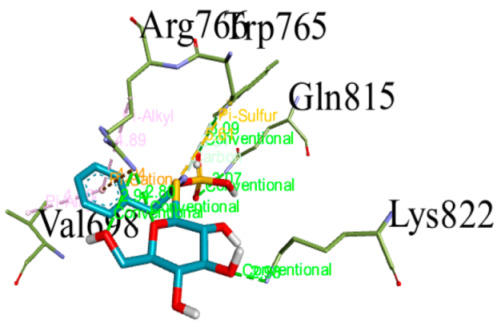	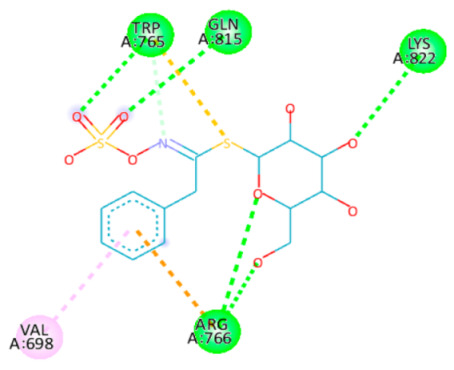
Binding interaction with Insulin-Like Growth Factor-1 Receptor (IGF-1R)				
6.	(−)-Epicatechin 3′-O-glucuronide	−8.4	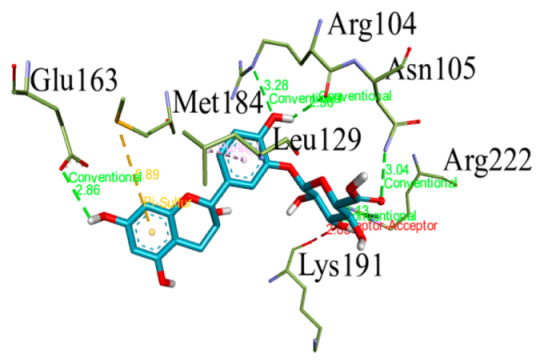	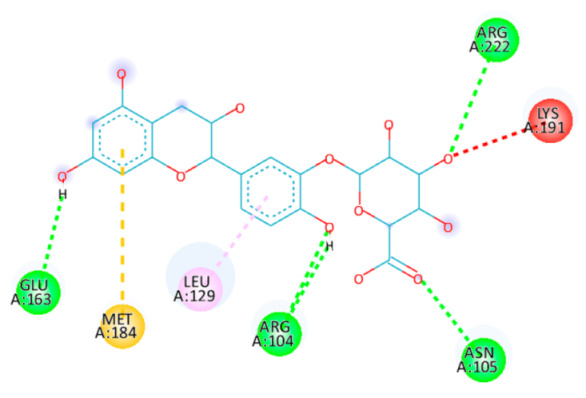
7.	Quercetin 3-(2-caffeoylglucuronoside)	−8.1	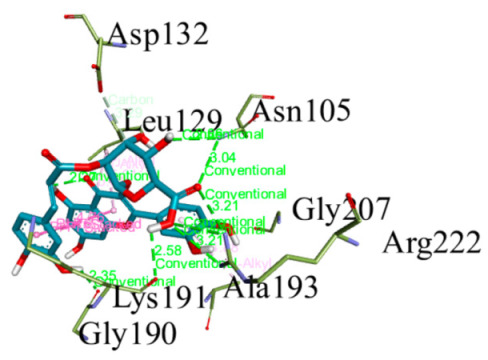	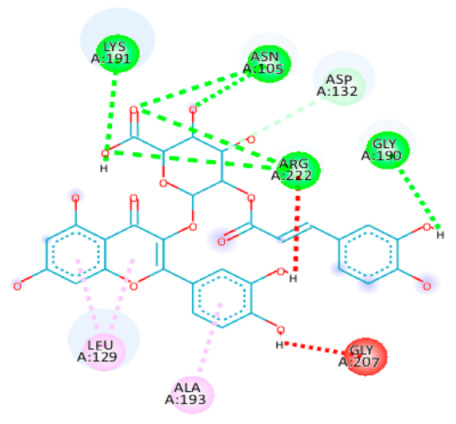
Binding interaction with Epidermal Growth Factor Receptor (EGFR/ERBB1)				
8.	Quercetin 3-(2-caffeoylglucuronoside)	−9.5	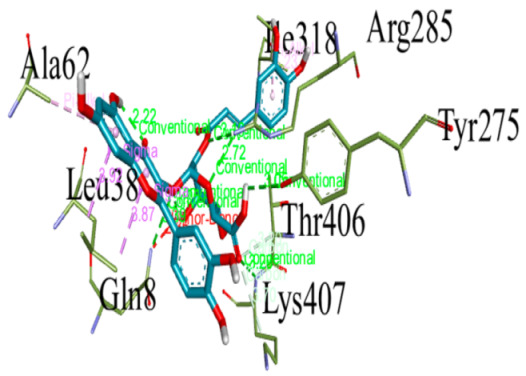	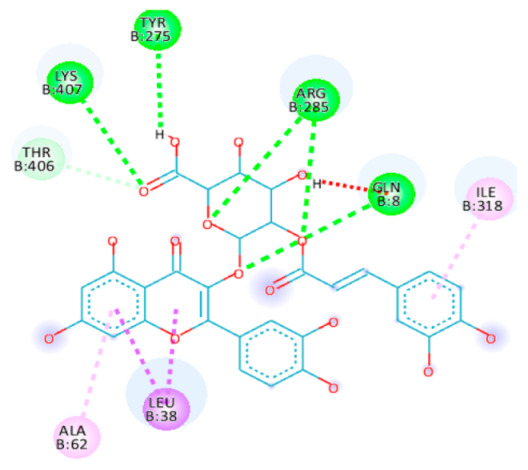
9.	(−)-Epicatechin 3′-O-glucuronide	−9.5	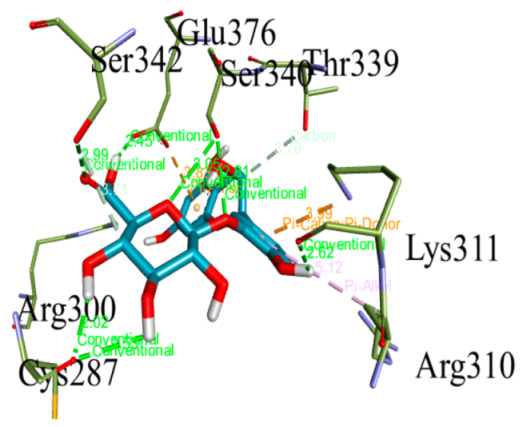	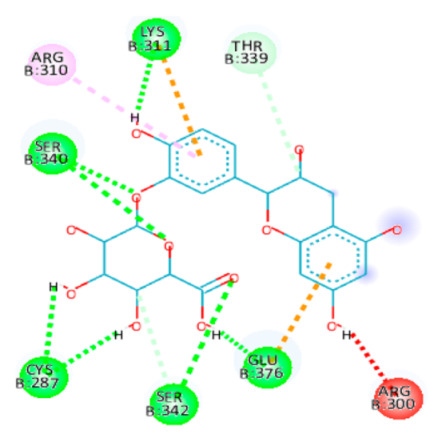
10.	Glucotropaeolin	−8.1	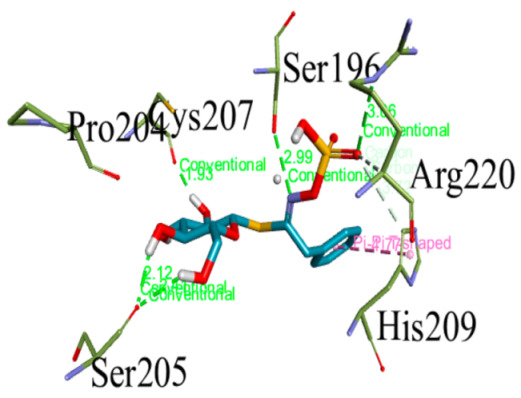	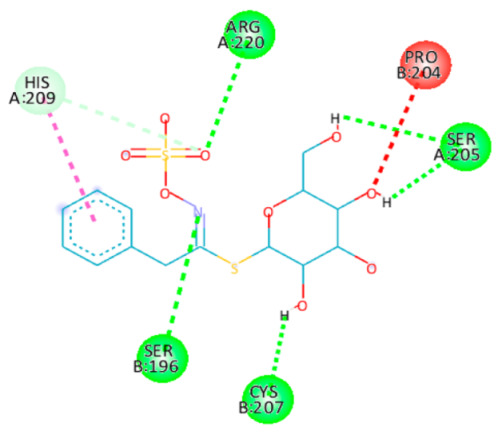
Binding interaction with Human epidermal growth factor receptor 3 (HER3)				
11.	Quercetin 3-(2-caffeoylglucuronoside)	−9.8	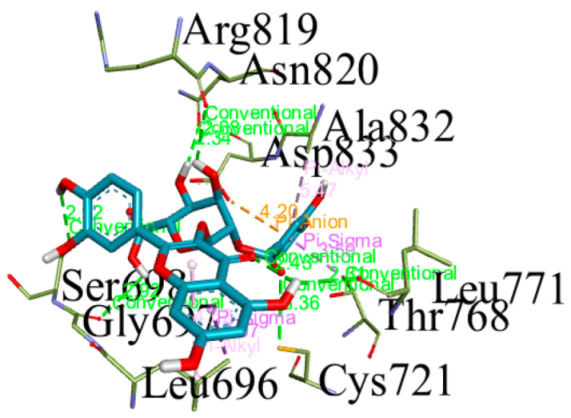	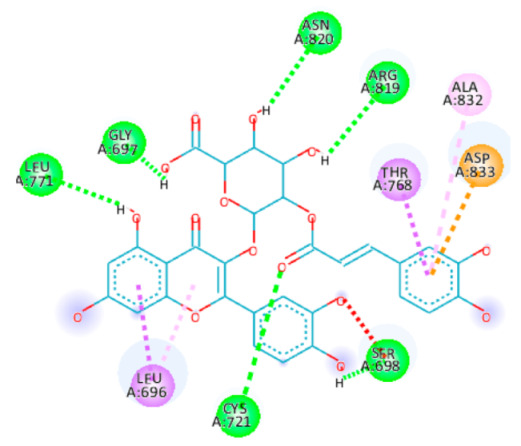
12.	(−)-Epicatechin 3′-O-glucuronide	−8.8	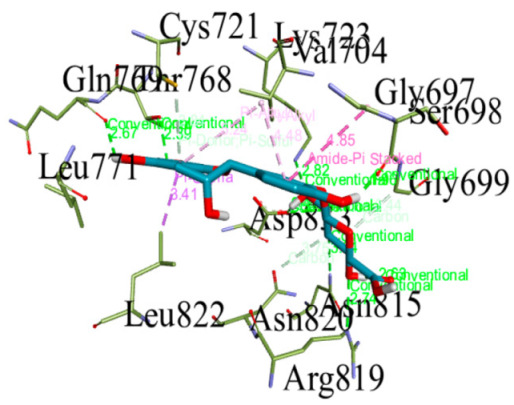	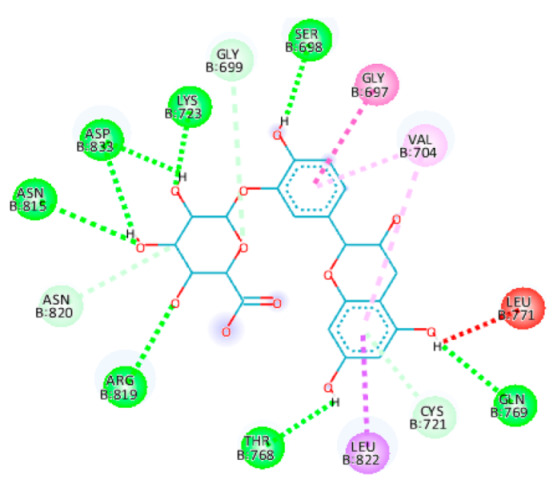
Binding interaction with Membrane progesterone receptor alpha (mPRα)				
13.	Quercetin 3-(2-caffeoylglucuronoside)	−10.2	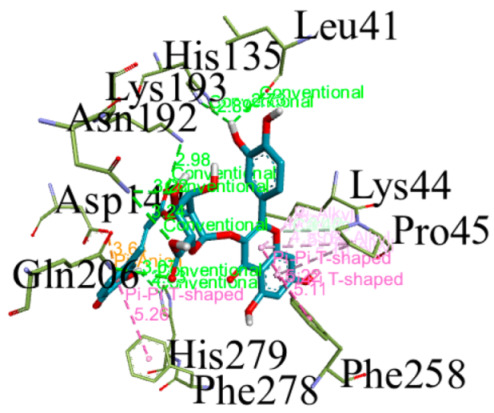	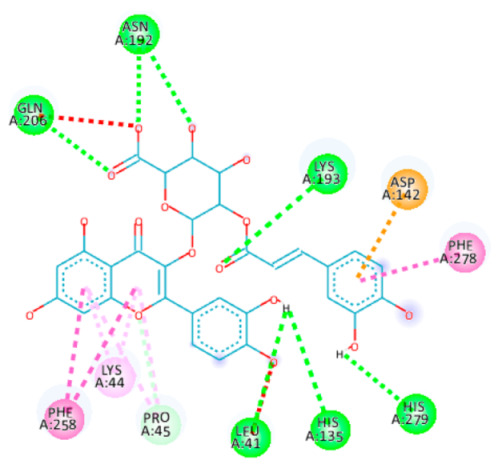
14.	(−)-Epicatechin 3′-O-glucuronide	−8.8	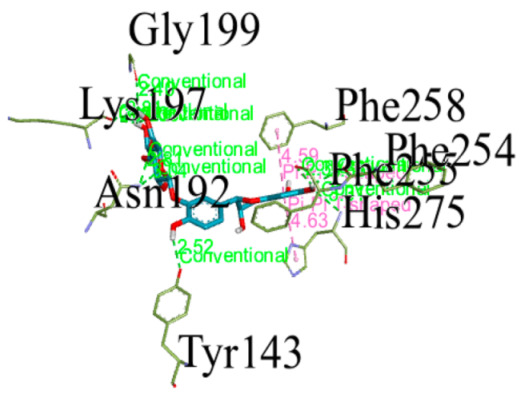	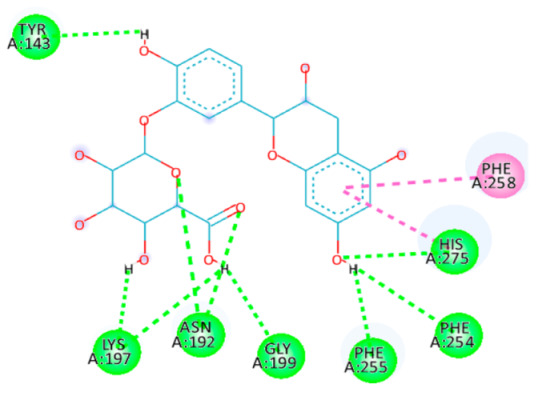
Binding interaction with G-Protein Coupled Estrogen Receptor (GPER/GPR30)				
15.	Quercetin 3-(2-caffeoylglucuronoside)	−7.8	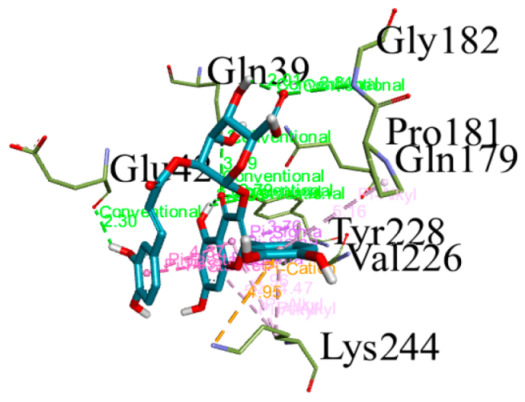	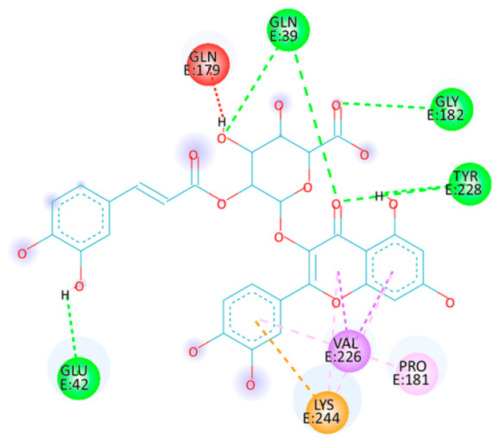
16.	(−)-Epicatechin 3′-O-glucuronide	−7.7	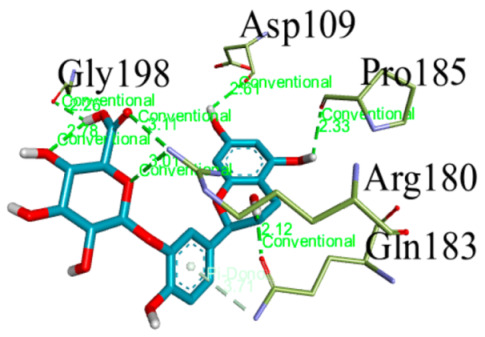	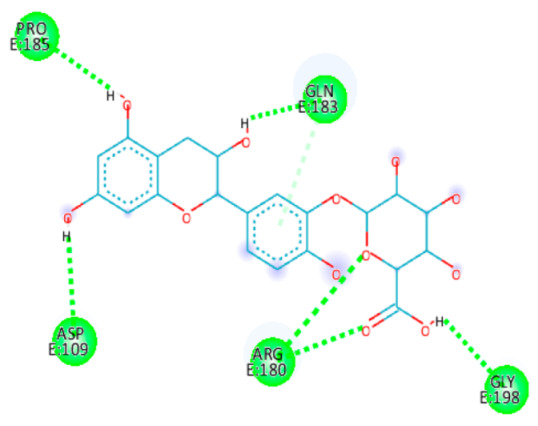

**Table 5:** Physicochemical Properties of *S. persica* L. root phytoconstituents obtained through HRLC-MS/MS at positive ion mode.

S. No.	Compounds Name	Formula	MW	Heavy Atoms	Aromatic Heavy Atoms	Fraction Csp^3^	Rotatable Bonds	H-Bond Acceptors	H-Bond Donors	MR	TPSA
1.	Medicanine	C7H_13_NO_3_	159.18	11	0	0.86	4	4	2	43.39	60.77
2.	3beta,6beta Dihydroxynortropane	C7H_13_NO_2_	143.18	10	0	1	0	3	3	40.58	52.49
3.	N-Acetyl-leucyl-leucin	C_14_H_26_N_2_O_4_	286.37	20	0	0.79	10	4	3	77.19	95.5
4.	Leucyl-Histidine	C_12_H_20_N_4_O_3_	268.31	19	5	0.58	8	5	4	69.49	121.1
5.	p-Coumaroylagmatine	C_14_H_20_N_4_O_2_	276.33	20	6	0.29	8	3	4	79.5	113.73
6.	5-Methoxydimethyltryptamine	C_13_H_18_N_2_O	218.29	16	9	0.38	4	2	1	67.07	28.26
7.	N(alpha)-gamma-L Glutamylhistamine	C_10_H_16_N_4_O_3_	240.26	17	5	0.5	8	5	4	59.88	121.1
8.	Europine	C_16_H_27_NO_6_	329.39	23	0	0.81	7	7	3	87.07	99.46
9.	(+)-alpha-Pinene	C_10_H_16_	136.23	10	0	0.8	0	0	0	45.22	0
10.	Asparaginyl-Cysteine	C_7_H_13_N_3_O_4_S	235.26	15	0	0.57	7	5	4	54.08	174.31
11.	Macamide B	C_23_H_39_NO	345.56	25	6	0.7	17	1	1	111.3	29.1
12.	Benzoxazinone glucoside	C_16_H_21_NO_11_	403.34	28	6	0.56	5	11	5	90.61	167.61
13.	N-(14-Methylhexadecanoyl) pyrolidine	C_21_H_41_NO	323.56	23	0	0.95	15	1	0	107.96	20.31
14.	Pipercitine	C_23_H_43_NO	349.59	25	0	0.87	16	1	0	117.1	20.31
15.	Benzyl isothiocyanate	C_8_H_7_NS	149.21	10	6	0.12	2	1	0	45.38	44.45

**Table 6:** Physicochemical properties of *S. persica* L. root phytoconstituents obtained through HRLC-MS/MS at negative ion mode.

S. No.	Molecule	Formula	MW	Heavy Atoms	Aromatic Heavy Atoms	Fraction Csp^3^	Rotatable Bonds	H-Bond Acceptors	H-Bond Donors	MR	TPSA
1.	Glucotropaeolin	C_14_H_19_NO_9_S_2_	409.43	26	6	0.5	7	10	5	91.57	199.79
2.	Quercetin 3-(2-caffeoylglucuronoside)	C_30_H_24_O_16_	640.5	46	22	0.17	8	16	9	154.16	274.11
3.	(−)-Epicatechin 3′-O-glucuronide	C_21_H_22_O_12_	466.39	33	12	0.38	4	12	8	107.07	206.6
4.	Benzyl isothiocyanate	C_8_H_7_NS	149.21	10	6	0.12	2	1	0	45.38	44.45
5.	8S-HODE	C_18_H_32_O_3_	296.44	21	0	0.72	14	3	2	90.63	57.53
6.	16-Hydroxy hexadecanoic acid	C_16_H_32_O_3_	272.42	19	0	0.94	15	3	2	81.96	57.53
7.	3-Keto stearic acid	C_18_H_34_O_3_	298.46	21	0	0.89	16	3	1	90.61	54.37

**Table 7:** Pharmacokinetic properties of *S. persica* L. root phytoconstituents obtained through HRLC-MS/MS at positive ion mode.

S. No.	Compounds Name	GI Absorption	BBB Permeant	Pgp Substrate	CYP1A2 Inhibitor	CYP2C19 Inhibitor	CYP2C9 Inhibitor	CYP2D6 Inhibitor	CYP3A4 Inhibitor	log Kp (cm/s)
1.	Medicanine	High	No	No	No	No	No	No	No	−9.1
2.	3beta,6beta Dihydroxynortropane	High	No	No	No	No	No	No	No	−7.66
3.	N-Acetyl-leucyl-leucin	High	No	No	No	No	No	No	No	−7.67
4.	Leucyl-Histidine	High	No	Yes	No	No	No	No	No	−9.48
5.	p-Coumaroylagmatine	High	No	No	No	No	No	No	No	−7.67
6.	5-Methoxydimethyltryptamine	High	Yes	No	Yes	No	No	Yes	No	−5.65
7.	N(alpha)-gamma-L Glutamylhistamine	High	No	No	No	No	No	No	No	−10.34
8.	Europine	High	No	Yes	No	No	No	No	No	−8.66
9.	(+)-alpha-Pinene	Low	Yes	No	No	No	Yes	No	No	−3.95
10.	Asparaginyl-Cysteine	Low	No	No	No	No	No	No	No	−11.18
11.	Macamide B	Low	No	No	Yes	Yes	No	Yes	Yes	−2.47
12.	Benzoxazinone glucoside	Low	No	No	No	No	No	No	No	−9.92
13.	N-(14-Methylhexadecanoyl) pyrrolidine	High	No	No	Yes	No	No	No	No	−2.74
14.	Pipercitine	Low	No	Yes	Yes	No	No	No	No	−2.14
15.	Benzyl isothiocyanate	High	Yes	No	No	No	No	No	No	−4.97

**Table 8:** Pharmacokinetic properties of *S. persica* root phytoconstituents obtained through HRLC-MS/MS at negative ion mode.

S. No.	Molecule	GI Absorption	BBB Permeant	Pgp Substrate	CYP1A2 Inhibitor	CYP2C19 Inhibitor	CYP2C9 Inhibitor	CYP2D6 Inhibitor	CYP3A4 Inhibitor	log Kp (cm/s)
1.	Glucotropaeolin	Low	No	Yes	No	No	No	No	No	−8.95
2.	Quercetin 3-(2-caffeoylglucuronoside)	Low	No	No	No	No	No	No	No	−8.39
3.	(−)-Epicatechin 3′-O-glucuronide	Low	No	No	No	No	No	No	No	−10
4.	Benzyl isothiocyanate	High	Yes	No	No	No	No	No	No	−4.97
5.	8S-HODE	High	Yes	No	No	No	No	No	No	−4.97
6.	16-Hydroxy hexadecanoic acid	High	Yes	No	No	No	No	Yes	No	−3.99
7.	3-Keto stearic acid	High	Yes	No	Yes	No	No	No	No	−3.17

**Table 9:** Toxicity potential analysis of *S. persica* L. root phytoconstituents obtained through HRLC-MS/MS at positive ion mode.

S. No.	Compounds Name	Mutagenic	Tumorigenic	Irritant	Reproductive Effect	Drug Likeness
1.	Medicanine	Green	Green	Green	Green	−1.03
2.	3beta,6beta Dihydroxynortropane	Green	Green	Green	Green	1.5
3.	N-Acetyl-leucyl-leucin	Green	Green	Green	Green	−14.2
4.	Leucyl-Histidine	Green	Green	Green	Green	−18.3
5.	p-Coumaroylagmatine	Green	Green	Green	Green	−1.17
6.	5-Methoxydimethyltryptamine	Green	Green	Green	Green	1.71
7.	N(alpha)-gamma-L Glutamylhistamine	Green	Green	Green	Green	−15.1
8.	Europine	Red	Green	Green	Green	4.55
9.	(+)-alpha-Pinene	Green	Green	Red	Green	−1.8
10.	Asparaginyl-Cysteine	Green	Green	Green	Green	−10.0
11.	Macamide B	Green	Green	Green	Green	−19.4
12.	Benzoxazinone glucoside	Green	Orange	Green	Green	−1.9
13.	N-(14-Methylhexadecanoyl) pyrolidine	Green	Green	Green	Green	−3.34
14.	Pipercitine	Green	Green	Green	Green	−18.7
15.	Benzyl isothiocyanate	Red	Orange	Green	Orange	−4.55

**Table 10:** Toxicity potential analysis of *S. persica* L. root phytoconstituents obtained through HRLC-MS/MS at negative ion mode.

S. No.	Compounds Name	Mutagenic	Tumorigenic	Irritant	Reproductive Effect	Drug Likeness
1.	Glucotropaeolin	Green	Green	Green	Red	−0.43
2.	Quercetin 3-(2-caffeoylglucuronoside)	Green	Green	Green	Green	−1.68
3.	(−)-Epicatechin 3′-O-glucuronide	Green	Green	Green	Green	−0.39
4.	Benzyl isothiocyanate	Red	Orange	Green	Orange	−4.55
5.	8S-HODE	Green	Green	Green	Green	−24.6
6.	16-Hydroxy hexadecanoic acid	Green	Green	Red	Green	−24.6
7.	3-Keto stearic acid	Green	Green	Green	Green	−27.6

## Data Availability

The original contributions presented in this study are included in the article/Supplementary Materials. Further inquiries can be directed to the corresponding authors.

## Supplementary Materials

The following supporting information can be downloaded at: https://www.mdpi.com/article/10.3390/ijms27135751/s1.

## Abbreviations

TNBC	Triple-negative breast cancer
UHPLC	Ultra-High Performance Liquid Chromatography
TFC	Total Flavonoid Contents
TPC	Total Phenolic Contents
MTT	3-(4,5-dimethylthiazol-2-yl)-2,5-diphenyltetrazolium bromide
ROS	Reactive oxygen species
MMP	Mitochondrial membrane potential
ERα	Estrogen Receptor Alpha
PR	Progesterone Receptor
EGFR	Epidermal Growth Factor Receptor
HER3	Human Epidermal Growth Factor Receptor 3
IGF-1R	Insulin-Like Growth Factor 1 Receptor
GPER	G Protein-Coupled Estrogen Receptor
FBS	fetal bovine serum
DCFH-DA	2′,7′-dichlorodihydrofluorescein diacetate
Rh123	Rhodamine 123
DMSO	Dimethyl Sulfoxide
DMEM/F-12	Dulbecco’s Modified Eagle Medium/Nutrient Mixture F-12
IC50	Half-Maximal Inhibitory Concentration
