# Supplementary material for: Anticancer Activity of Miswak Root Extract in Breast Cancer Cell Line: HRLC-MS/MS Profiling, In Vitro Evaluation, and In Silico Analysis
Source: Int J Mol Sci. 2026 Jun 25;27(13):5751. doi: 10.3390/ijms27135751 (PMC13362529; doi:10.3390/ijms27135751)
Supplement: Supplementary file 1 [file ijms-27-05751-s001.zip › ijms-4325257-supplementary.pdf]

Supplementary Data

Table S1. Docking Grid Parameters for Targeted Receptor Proteins of Breast Cancer Cells.

S. No.	Receptor Proteins	PDB ID/UNIPROT PDB ID	Center (x, y, z) Å	Dimensions (x, y, z) Å
1	Estrogen Receptor α (ERα / ESR1) 1ERR	1R5K	-12.6199<br>47.5369<br>344.2233	50.1956<br>46.5369<br>49.9821
2	Progesterone receptor (PR)	4OAR	7.3569<br>32.6613<br>12.0236	150<br>171<br>103
3	Insulin-Like Growth Factor-1 Receptor (IGF-1R)	1IGR	40.0180<br>29.4763<br>52.6054	54.2532<br>99.0955<br>80.4993
4	Epidermal Growth Factor Receptor (EGFR / ERBB1)	1IVO	83.7200<br>56.2037<br>50.1817	122.0712<br>99.9033<br>71.8433
5	Human Epidermal Growth Factor Receptor 3 (HER3)	3KEX	-0.0974<br>-1.5834<br>-18.8019	103.1080<br>55.4982<br>57.7400
6	Progesterone Receptor (PR) – Membrane-Associated mPRs Membrane progesterone receptor alpha (mPRα)	Q86WK9	-17.8377<br>2.3085<br>-8.0843	108.9797<br>53.5160<br>74.5650
7	G-Protein Coupled Estrogen Receptor (GPER / GPR30)	8XOG	95.3170<br>88.9648 110.2016	70.6051<br>86.1638<br>102.3255

Table S2. Molecular docking and interaction studies of chemical compounds identified at Positive ion mode with therapeutic membrane receptors of breast cancer, viz. Estrogen Receptor α (ERα / ESR1; PDB ID: 1R5K), Progester-one receptor (PR; PDB ID: 4OAR), Insulin-Like Growth Factor-1 Receptor (IGF-1R; PDB ID: 1IGR), Epidermal Growth Factor Receptor (EGFR / ERBB1; PDB ID: 1IVO), Human Epidermal growth factor Receptor 3 (HER3; PDB ID: 3KEX), Membrane progesterone receptor alpha (mPRα; PDB identifier/UniProt entry Q86WK9) and G-Protein Coupled Estrogen Receptor (GPER/ GPR30; PDB ID: 8XOG) through PyRx docking tool.

**Molecular docking with Estrogen Receptor α (ERα / ESR1)**				
**S. No.**	**Compounds Name (PubChem CID)**	**Binding Affinity (Kcal/mol)**	**3D interaction**	**2D Interaction**
1.	Macamide B (11198769)	-7.3	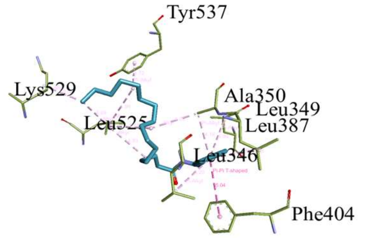	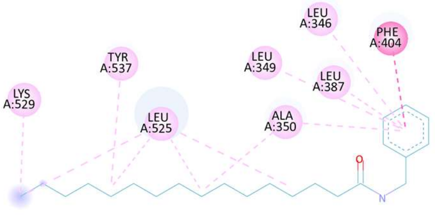
2.	Benzoxazinone glucoside (77195081)	-7.2	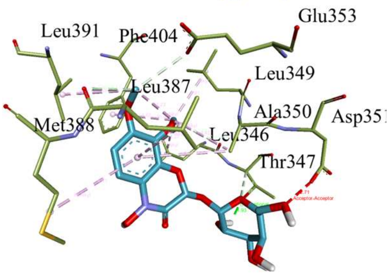	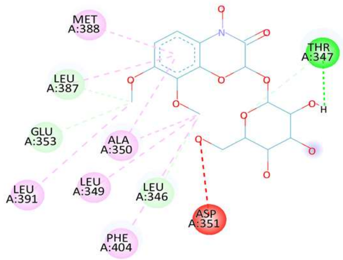
3.	N-(14-Methylhexadecanoyl)pyrrolidine (6430518)	-7	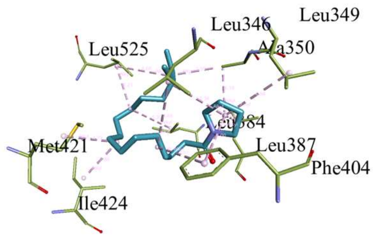	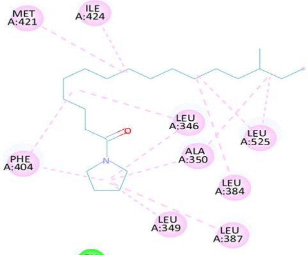
4.	N-Acetyl-leucyl-leucin (443129)	-6.7	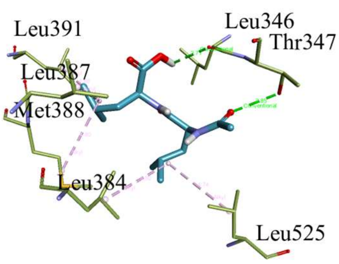	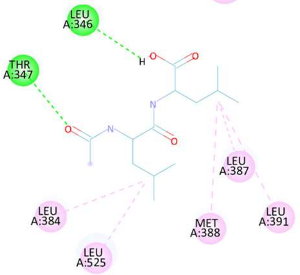
5.	5-Methoxydimethyltryptamine (1832)	-6.3	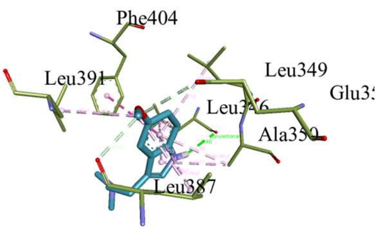	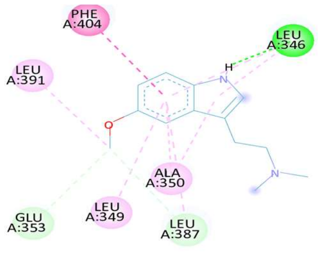

6.	Leucyl-Histidine (6992828)	-6.3	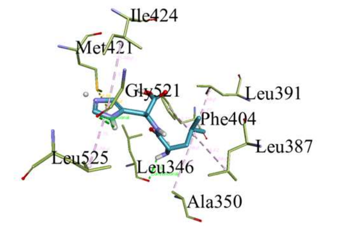	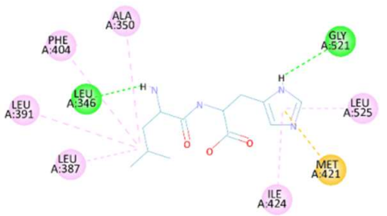
7.	Asparaginyl-Cysteine (18218178)	-6.1	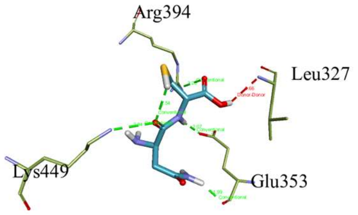	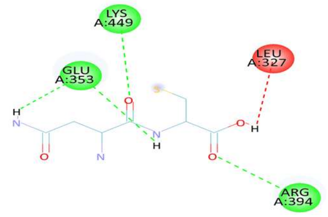
8.	N(alpha)-gamma-Lglutamylhistamine (440238)	-6	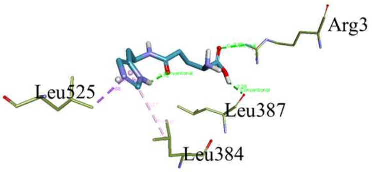	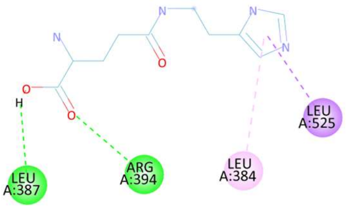
9.	Europine (5462451)	-5.5	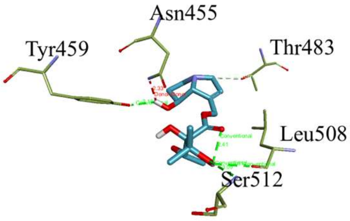	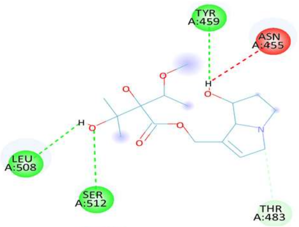
10.	(+)-alpha-Pinene (82227)	-5.5	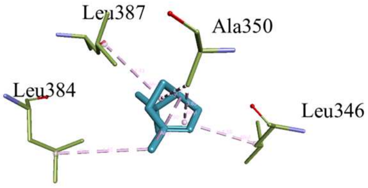	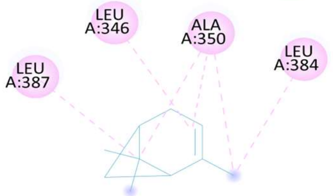
11.	Benzyl isothiocyanate (2346)	-5.4	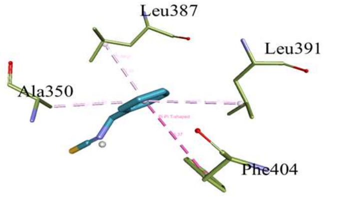	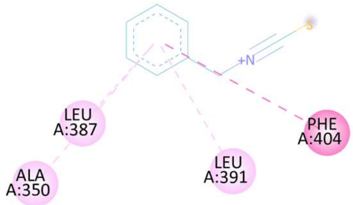
12.	3beta,6beta Dihydroxynortropane (22297531)	-5	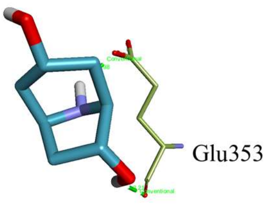	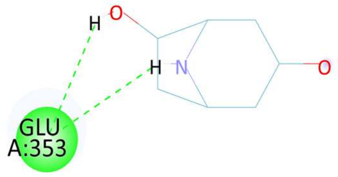
13.	Medicanine (101409750)	-4.8	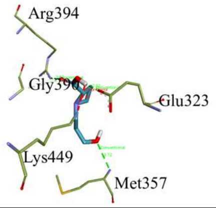	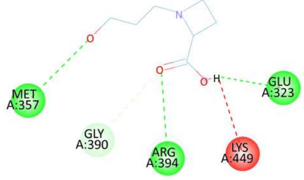

Molecular docking with Progesterone receptor

1.	Europine (5462451)	-6.9	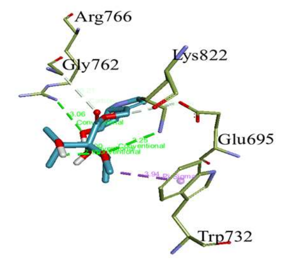	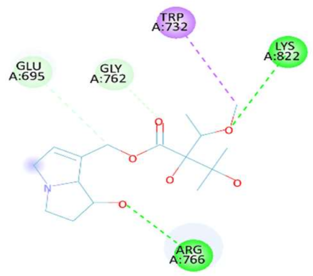
2.	Leucyl-Histidine (6992828)	-6.7	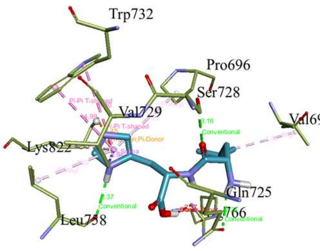	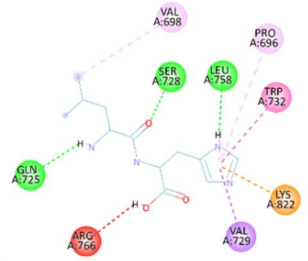
3.	Macamide B (11198769)	-6.5	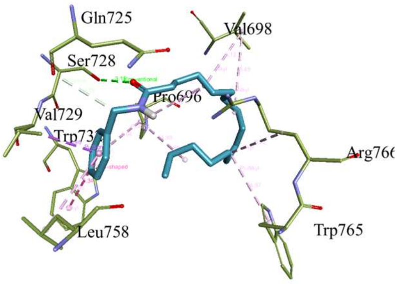	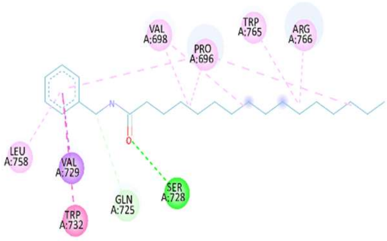
4.	p-Coumaroylagmatine (440362)	-6.4	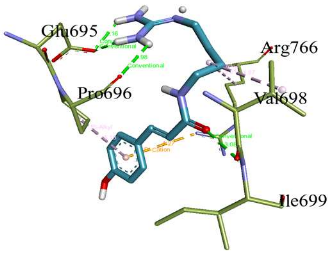	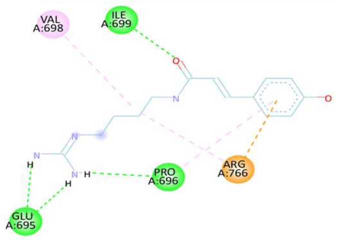
5.	N-Acetyl-leucyl-leucin (443129)	-6.4	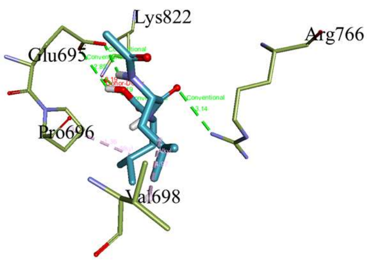	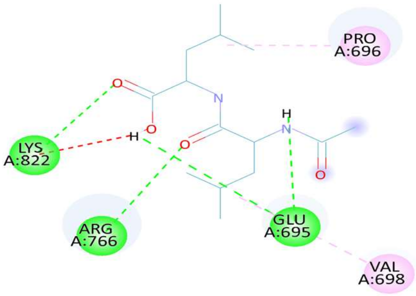
6.	N-(14-Methylhexadecanoyl)pyrrolidine (6430518)	-6.4	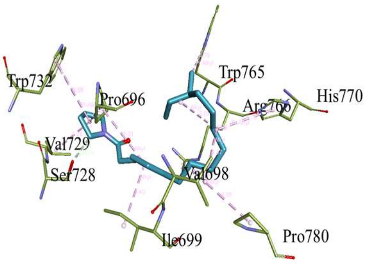	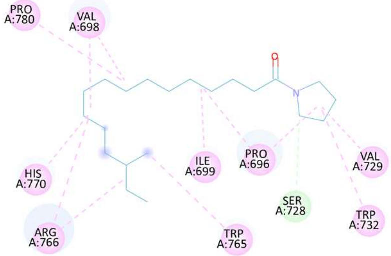

7.	(+)-alpha-Pinene (82227)	-6.4	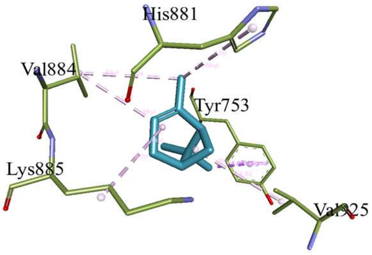	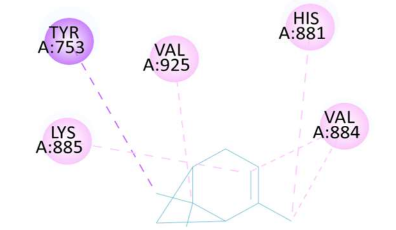
8.	5-Methoxydimethyltryptamine (1832)	-6.3	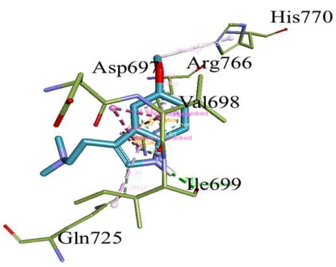	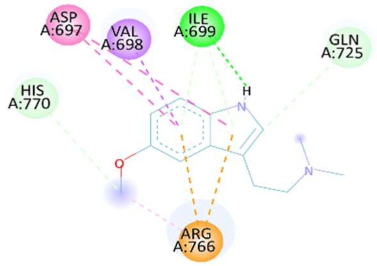
9.	3beta,6beta Dihydroxynortropane (22297531)	-6.2	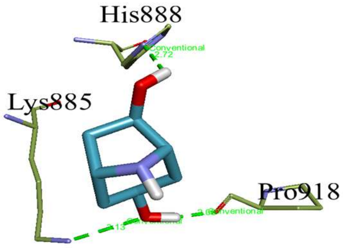	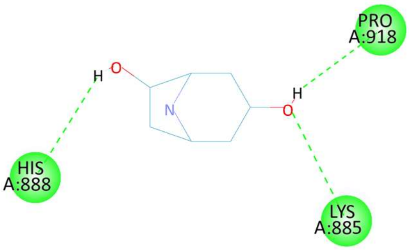
10.	N(alpha)-gamma-Lglutamylhistamine (440238)	-6	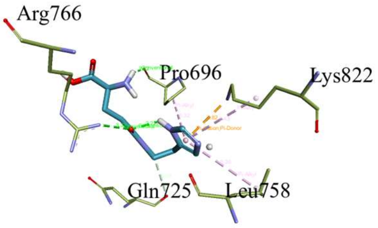	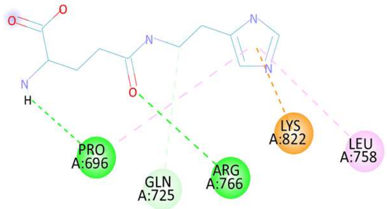
11.	Pipercitine (12575258)	-5.7	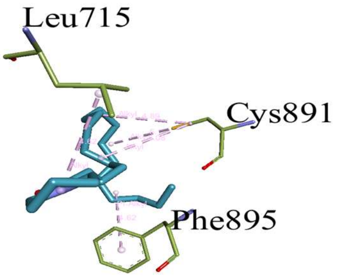	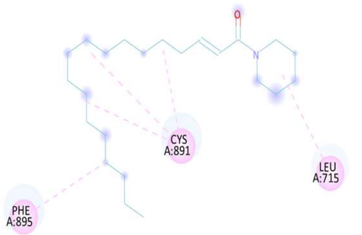
12.	Medicanine (101409750)	-5.6	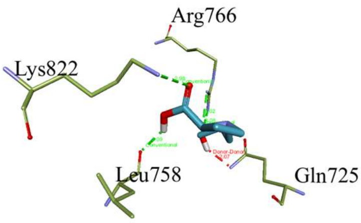	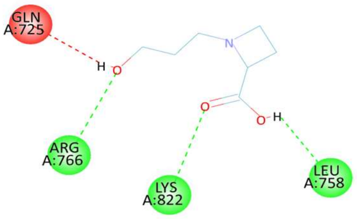

13.	Asparaginyl -Cysteine (18218178)	-5.6	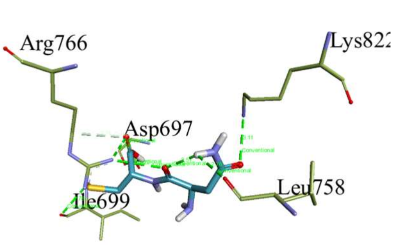	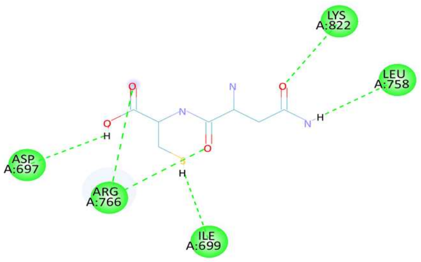
14.	Benzyl isothiocyanate (2346)	-4.8	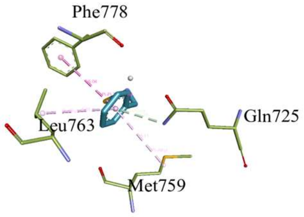	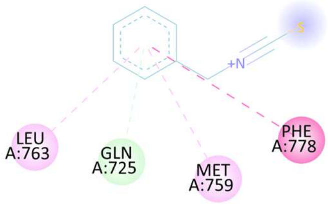
**Molecular docking with Insulin-Like Growth Factor-1 Receptor (IGF-1R)**				
1.	p-Coumaroylagmatine (440362)	-6.2	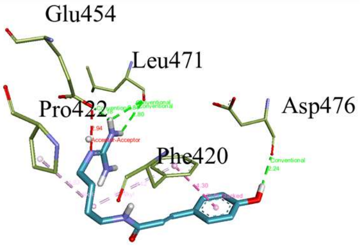	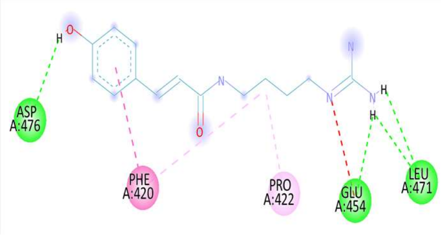
2.	Leucyl-Histidine (6992828)	-6.1	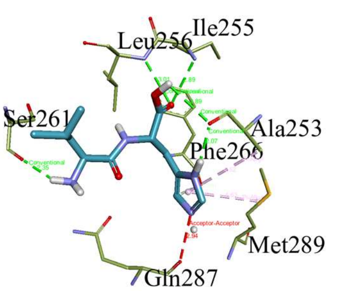	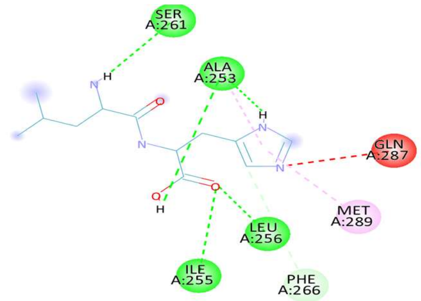
3.	5-Methoxydimethyltryptamine (1832)	-5.9	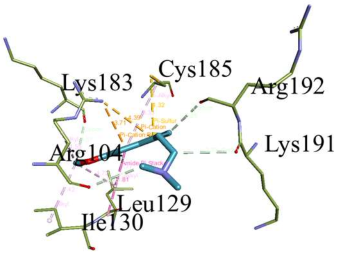	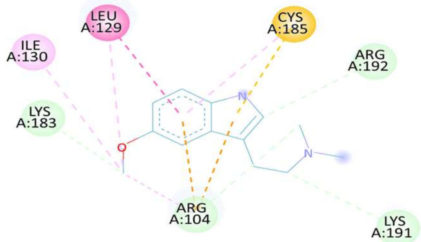
4.	Europine (5462451)	-5.6	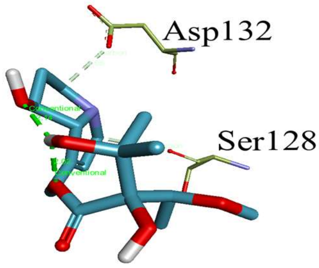	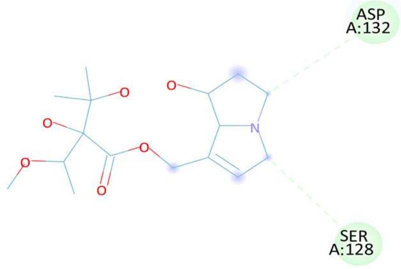

5.	N(alpha)-gamma-Lglutamylhistamine (440238)	-5.3	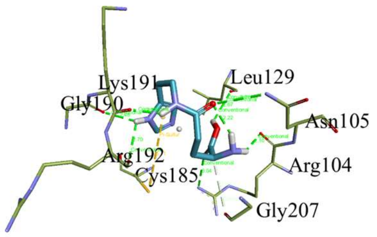	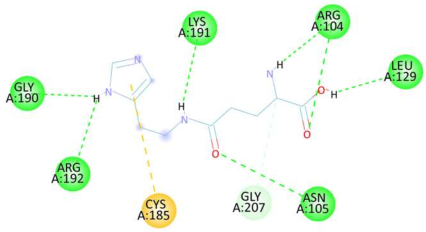
6.	N-Acetyl-leucyl-leucin (443129)	-5.3	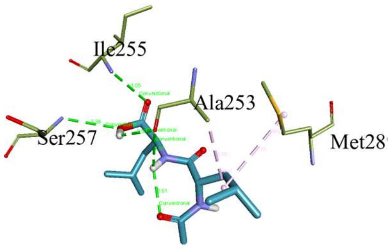	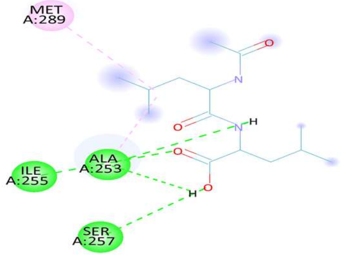
7.	(+)-alpha-Pinene (82227)	-5.3	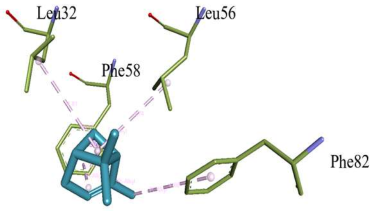	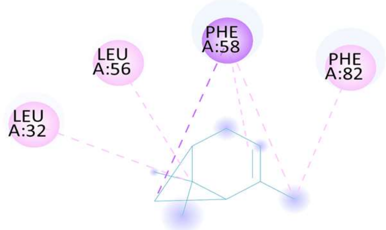
8.	Macamide B (11198769)	-5.1	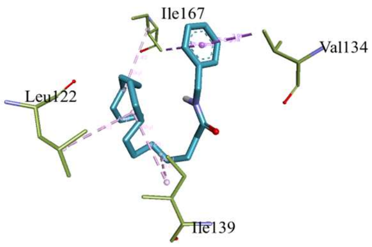	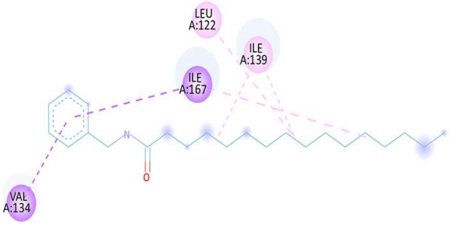
9.	N-(14-Methylhexadecanoyl)pyrrolidine (6430518)	-5	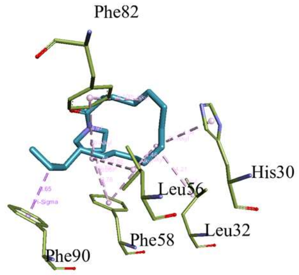	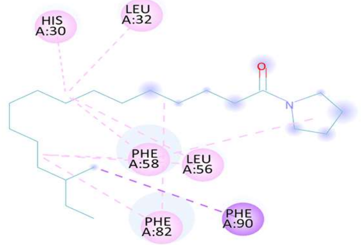
10.	Medicanine (101409750)	-4.9	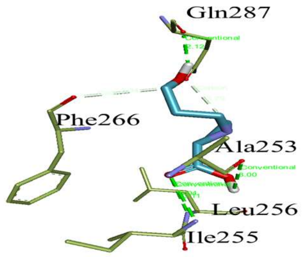	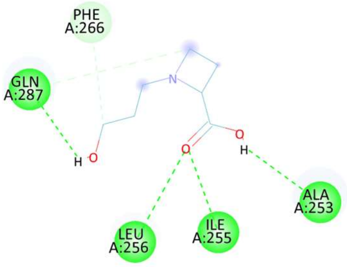
11.	3beta,6beta Dihydroxynortropane (22297531)	-4.9	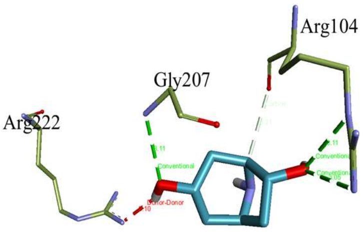	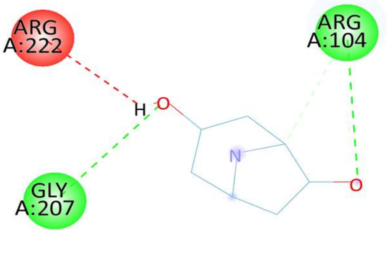

12.	Asparaginyl -Cysteine (18218178)	-4.6	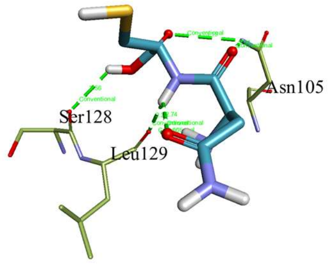	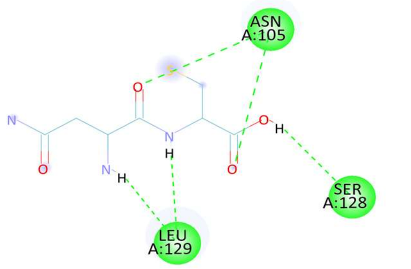
13.	Pipercitine (12575258)	-4.5	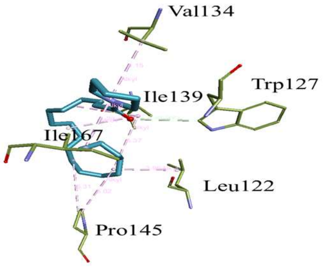	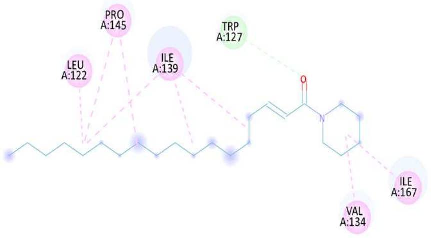
14.	Benzyl isothiocyana te (2356)	-4.3	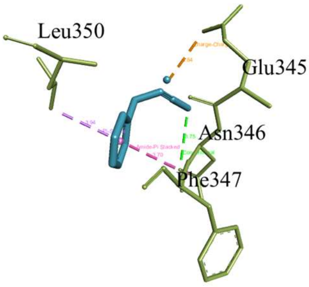	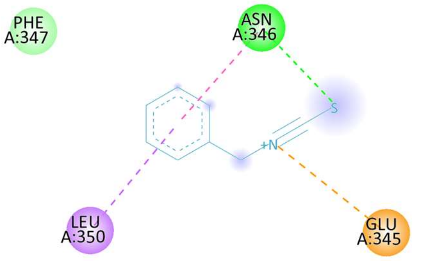
**Molecular docking with Epidermal Growth Factor Receptor (EGFR / ERBB1)**				
1.	p-Coumaroyla gmatine (440362)	-6.7	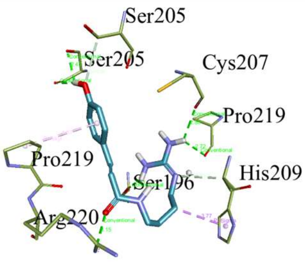	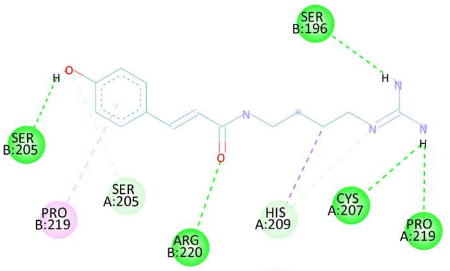
2.	Leucyl-Histidine (6992828)	-6.7	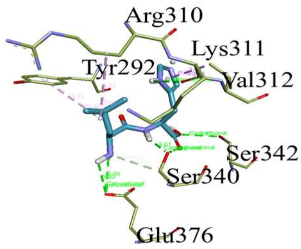	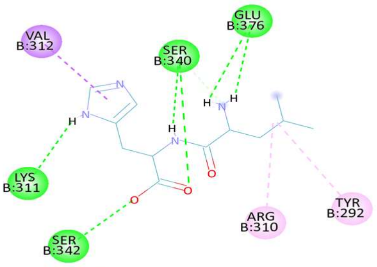
3.	N-Acetyl-leucyl-leucin (443129)	-6.4	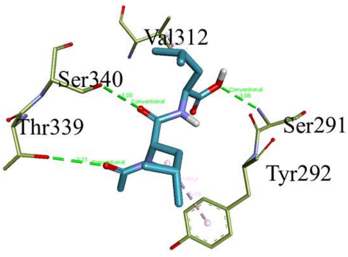	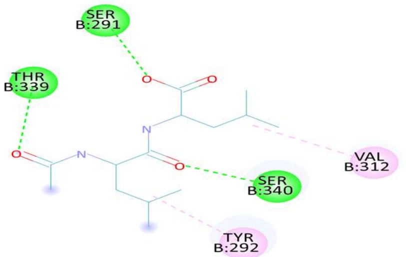

4.	Europine (5462451)	-6.2	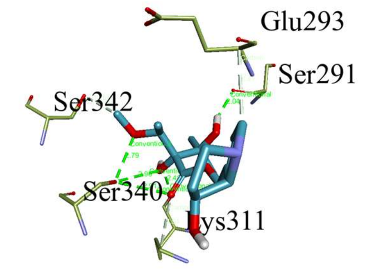	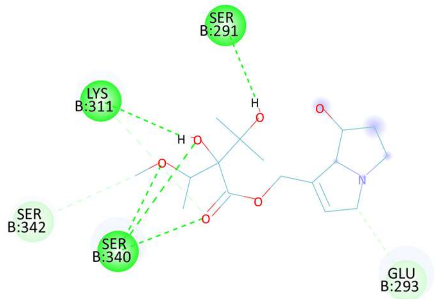
5.	5-Methoxydimethyltryptamine (1832)	-6.1	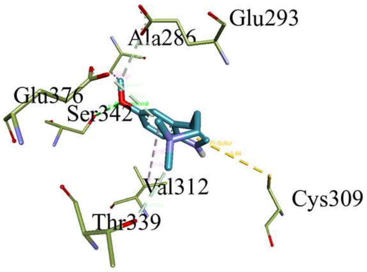	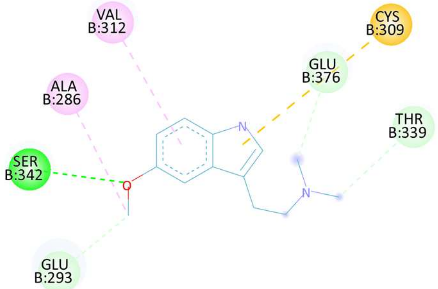
6.	N(alpha)-gamma-Lglutamylhistamine (440238)	-5.9	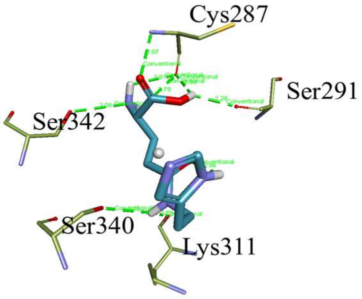	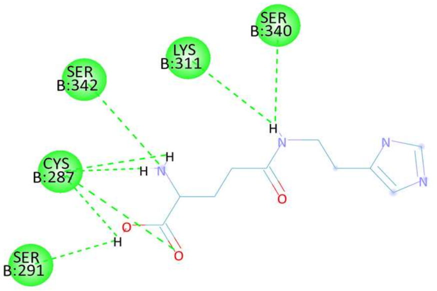
7.	Pipercitine (12575258)	-5.5	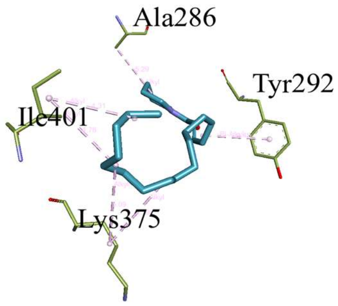	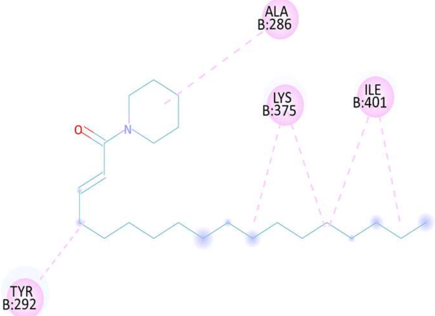
8.	Asparaginyl-Cysteine (18218178)	-5.5	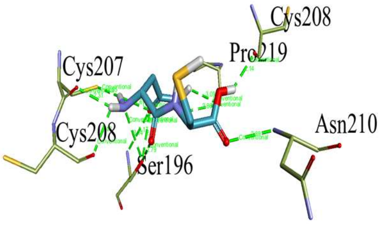	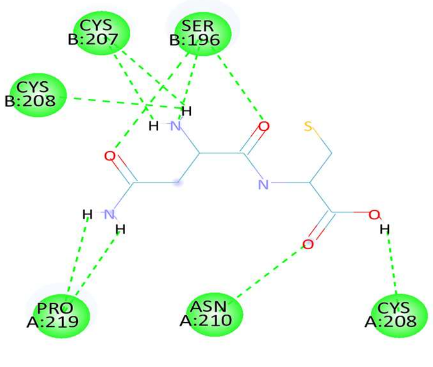

9.	3beta,6beta Dihydroxynortropane (22297531)	-5.4	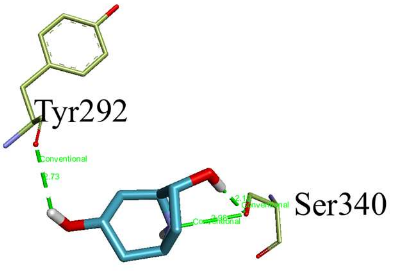	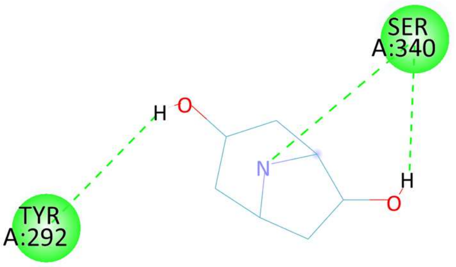
10.	Macamide B (11198769)	-5.3	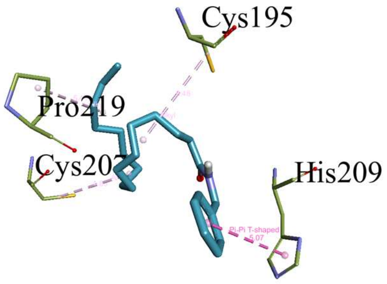	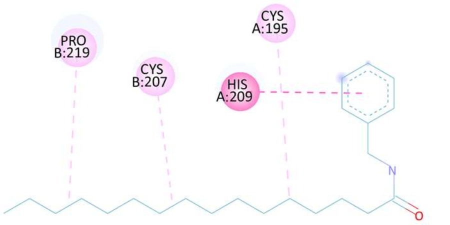
11.	N-(14-Methylhexadecanoyl)pyrrolidine (6430518)	-5.1	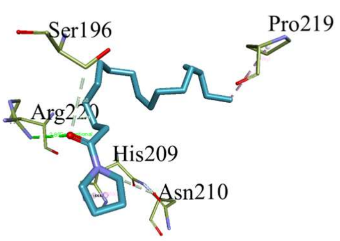	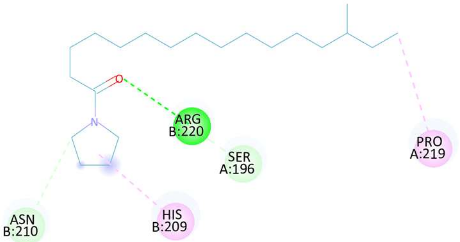
12.	(+)-alpha-Pinene (82227)	-5	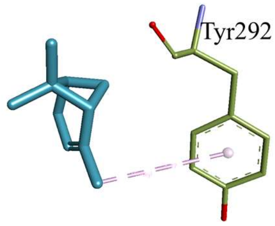	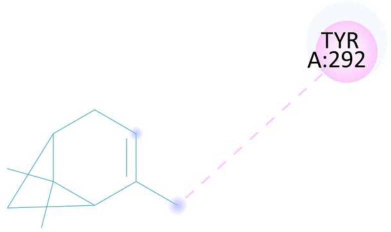
13.	Medicanine (101409750)	-4.7	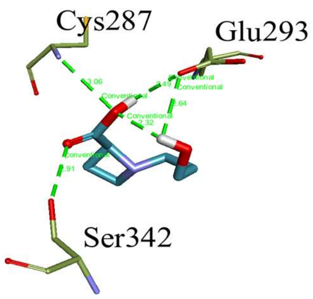	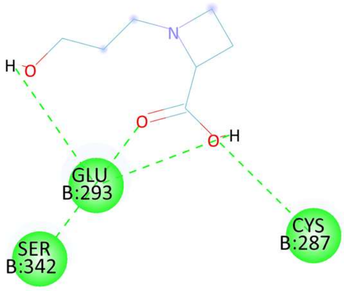
14.	Benzyl isothiocyanate (2346)	-4.7	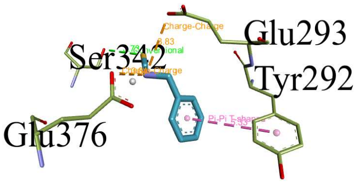	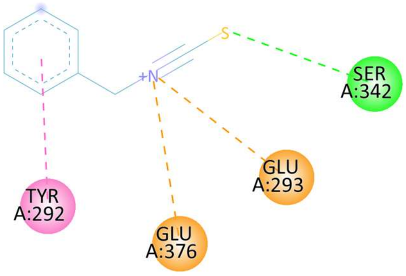
**Molecular docking with human epidermal growth factor Receptor 3 (HER3)**				

1.	Macamide B (11198769)	-7	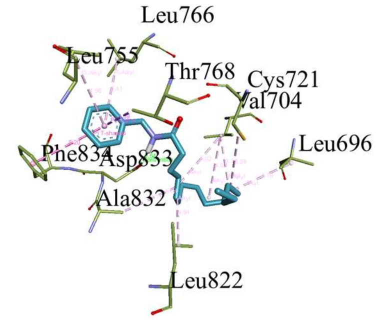	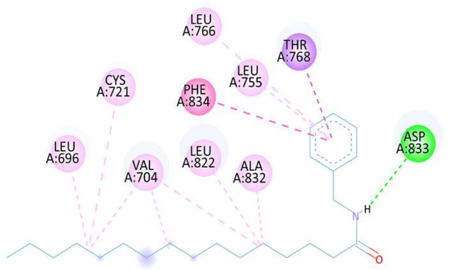
2.	Leucyl-Histidine (6992828)	-6.8	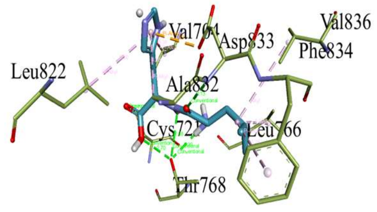	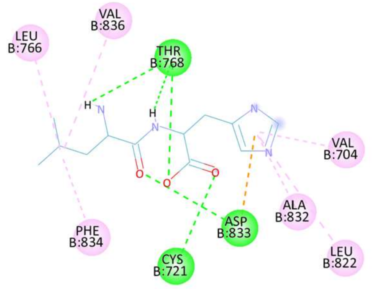
3.	Pipercitine (12575258)	-6.7	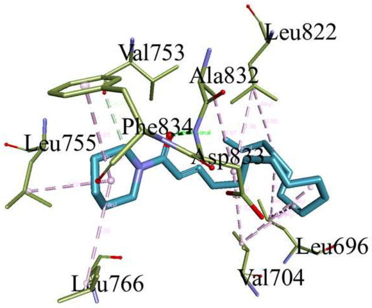	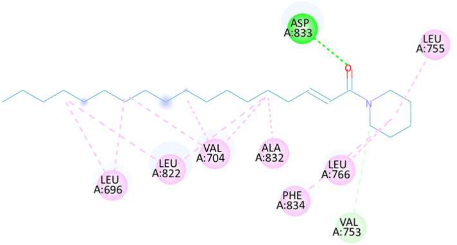
4.	N(alpha)-gamma-Lglutamylhistamine (440238)	-6.6	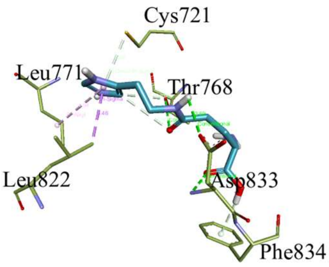	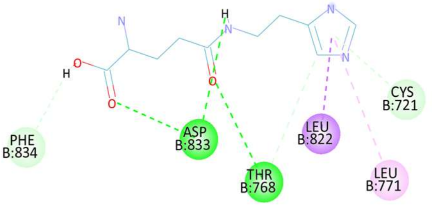
5.	N-Acetyl-leucyl-leucin (443129)	-6.3	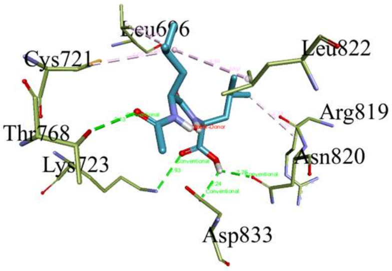	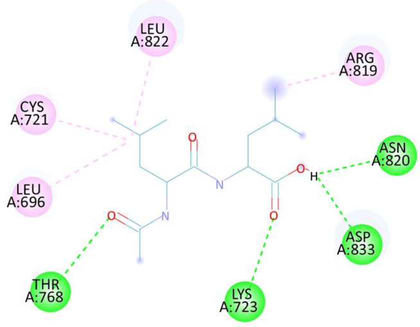

6.	N-(14-Methylhexadecanoyl)pyrrolidine (6430518)	-6.3	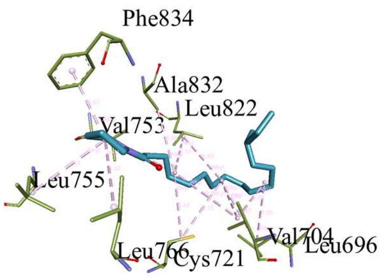	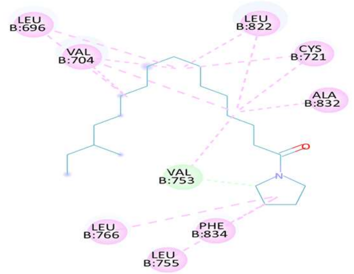
7.	5-Methoxydimethyltryptamine (1832)	-6	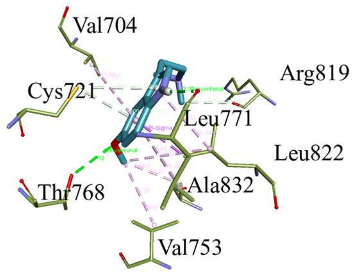	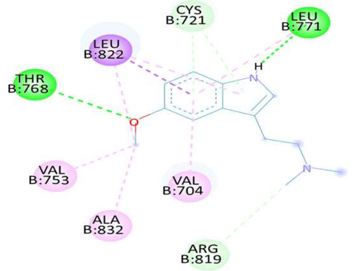
8.	Europine (5462451)	-6	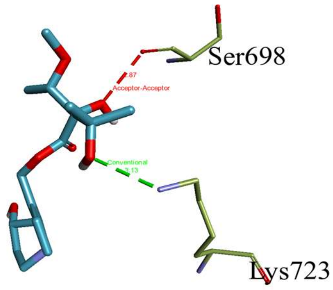	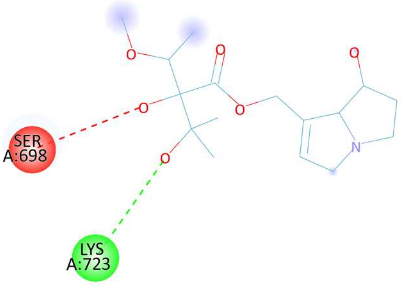
9.	(+)-alpha-Pinene (82227)	-5.6	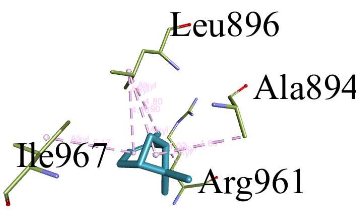	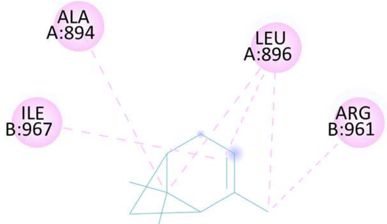
10.	Benzyl isothiocyanate (2346)	-5.6	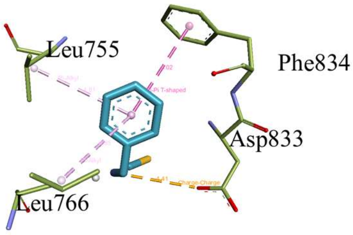	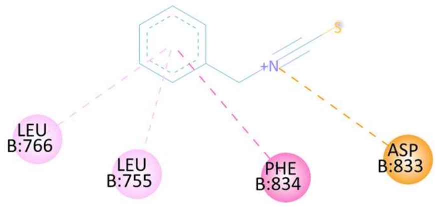
11.	Asparaginyl-Cysteine (18218178)	-5.3	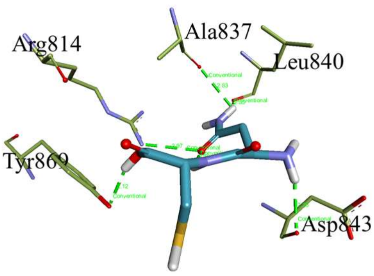	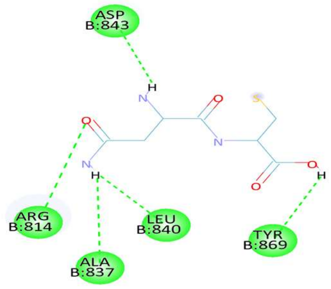

12.	3beta,6beta Dihydroxynortropane (22297531)	-5.3	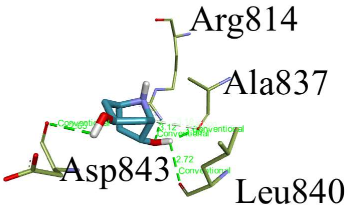	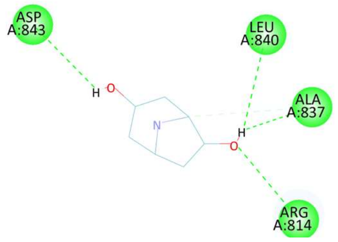
13.	Medicanine (101409750)	-5.1	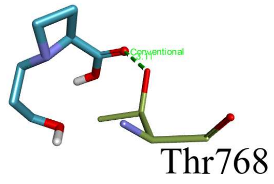	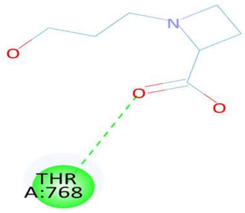
**Molecular docking with Membrane progesterone receptor alpha (mPR*α*)**				
1.	Macamide B (11198769)	-7.5	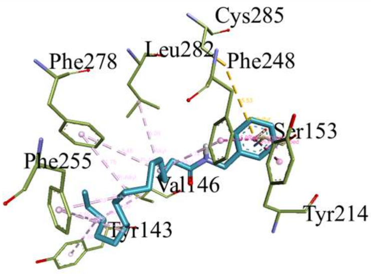	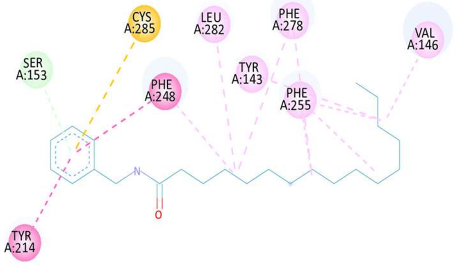
2.	Benzoxazinone glucoside (77195081)	-7.4	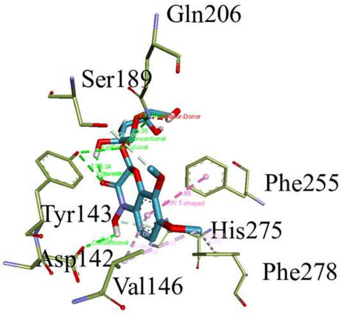	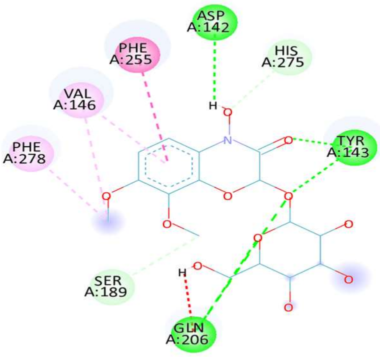
3.	N-Acetyl-leucyl-leucin (443129)	-6.8	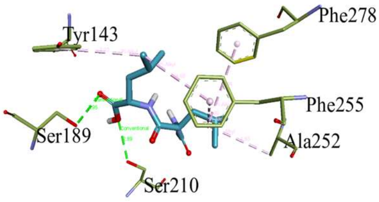	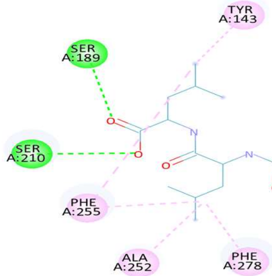
4.	N-(14-Methylhexadecanoyl)pyrrolidine (6430518)	-6.6	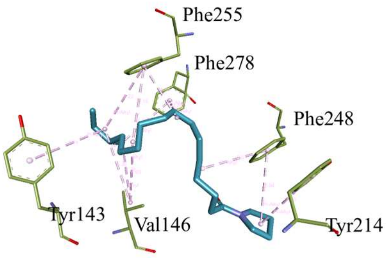	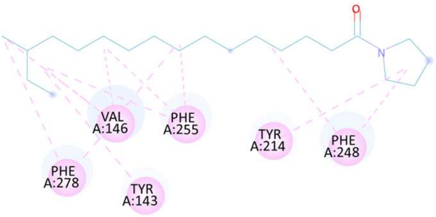

5.	5-Methoxydimethyltryptamine (1832)	-6	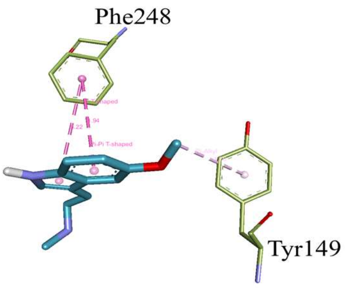	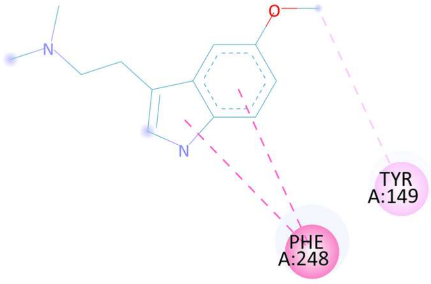
6.	N(alpha)-gamma-Lglutamylhistamine (440238)	-5.9	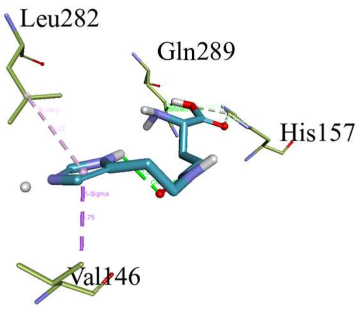	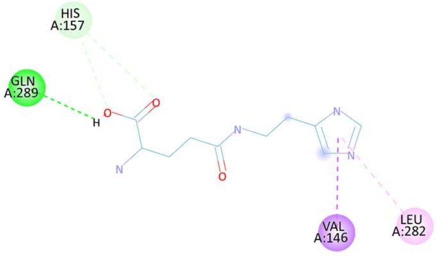
7.	Leucyl-Histidine (6992828)	-5.9	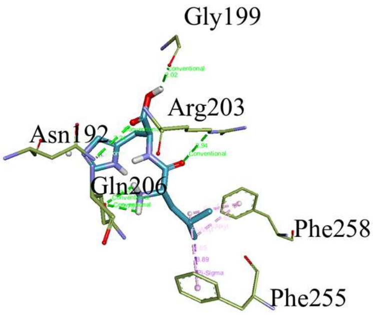	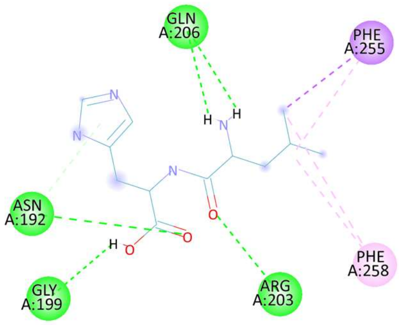
8.	(+)-alpha-Pinene (82227)	-5.7	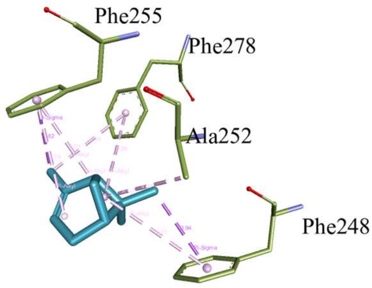	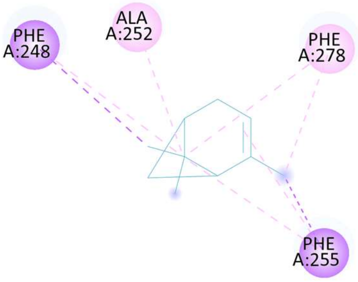
9.	Medicanine (101409750)	-5.3	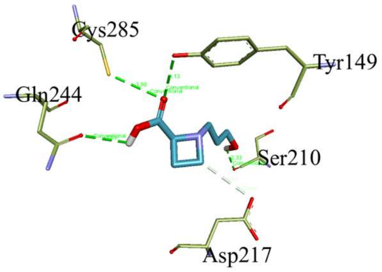	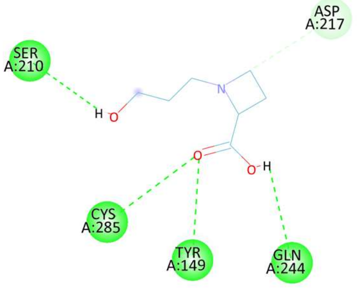
10.	Europine (5462451)	-5.3	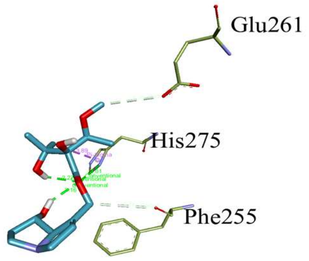	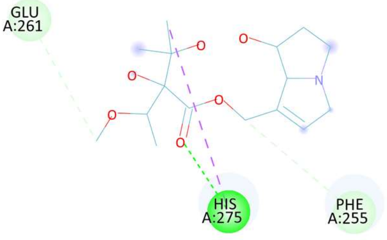

11.	Pipercitine (12575258)	-5.2	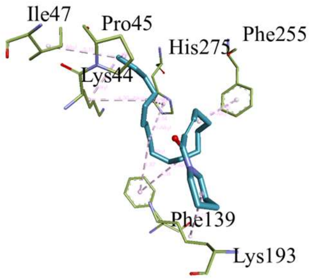	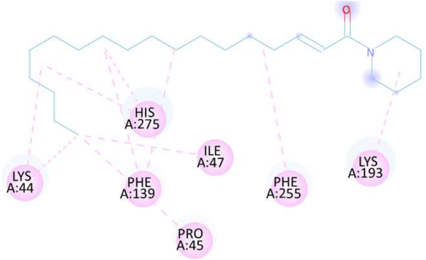
12.	3beta,6beta Dihydroxynortropane (22297531)	-5	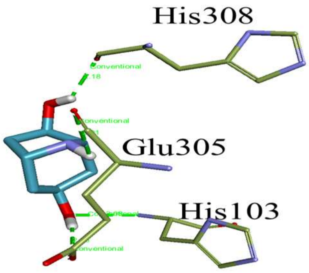	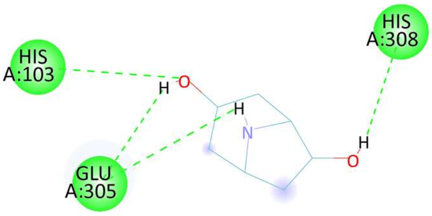
13.	Asparaginyl -Cysteine (18218178)	-4.9	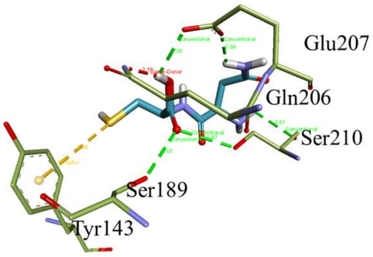	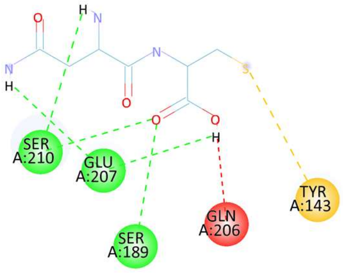
14.	Benzyl isothiocyanate (2346)	-4.5	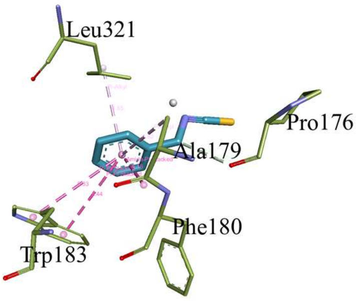	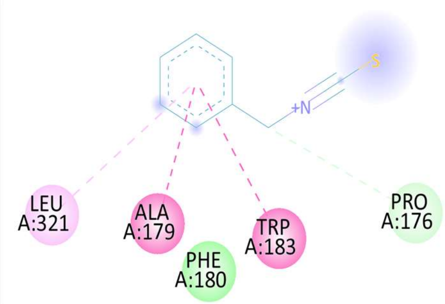
**Molecular docking with G-Protein Coupled Estrogen Receptor (GPER / GPR30)**				
1.	p-Coumaroylagmatine (440362)	-6.5	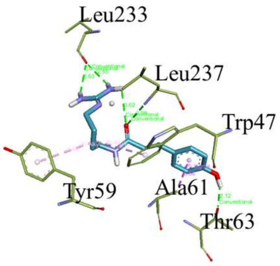	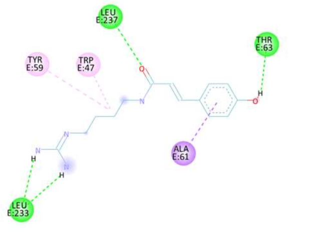
2.	Pipercitine (12575258)	-6.2	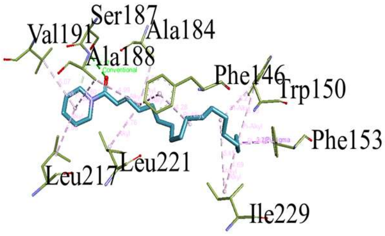	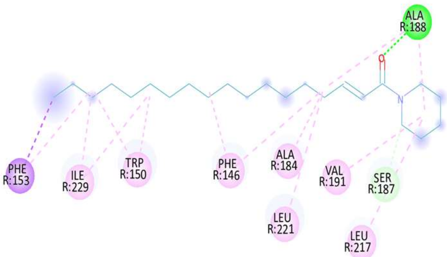

3.	Macamide B (11198769)	-6.1	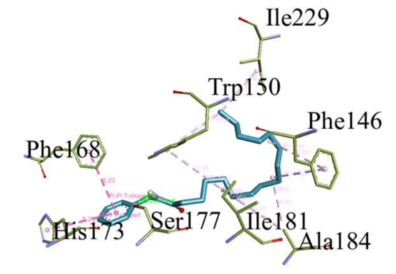	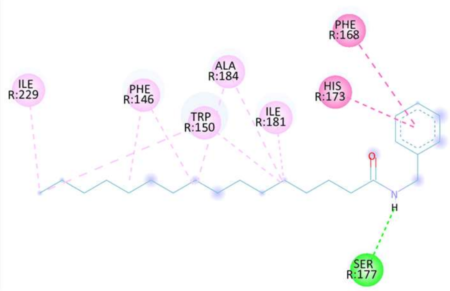
4.	Europine (5462451)	-6.1	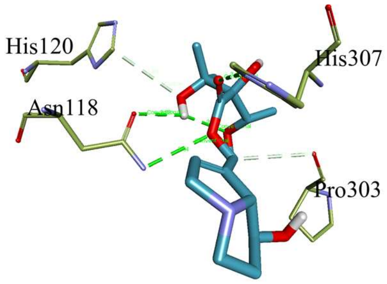	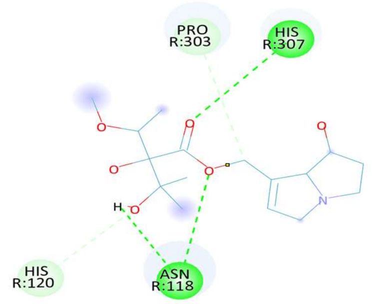
5.	N-(14-Methylhexadecanoyl)pyrrolidine (6430518)	-5.8	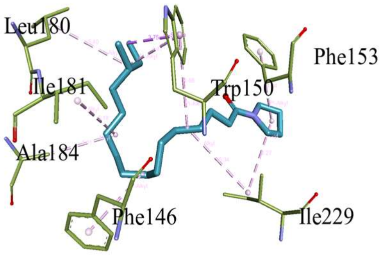	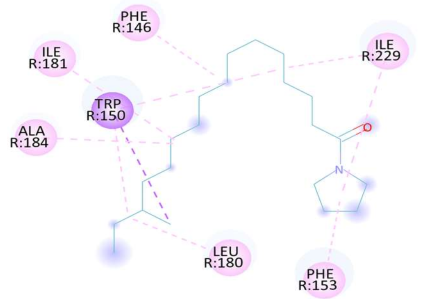
6.	Leucyl-Histidine (6992828)	-5.8	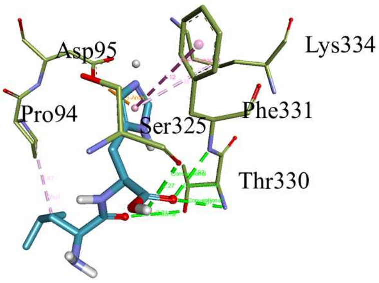	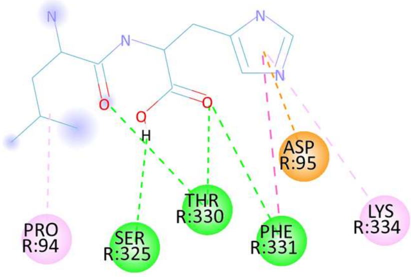
7.	N-Acetyl-leucyl-leucin (443129)	-5.6	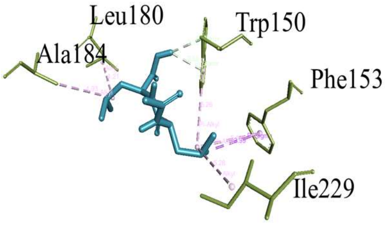	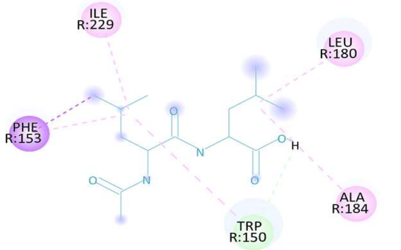
8.	5-Methoxydimethyltryptamine (1832)	-5.4	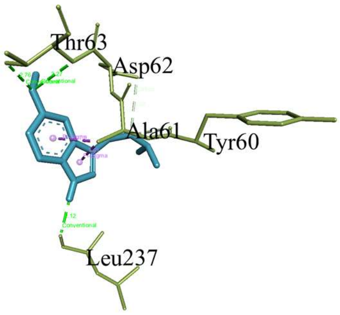	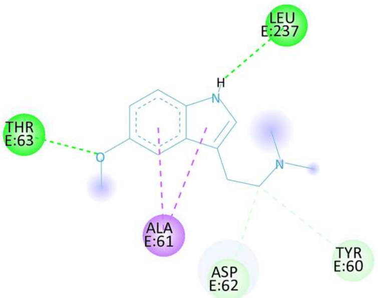

9.	N(alpha)-gamma-Lglutamylhistamine (440238)	-5.3	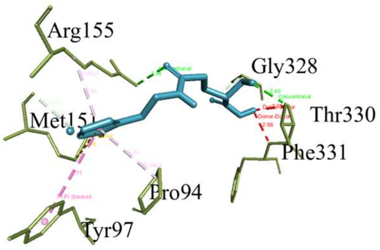	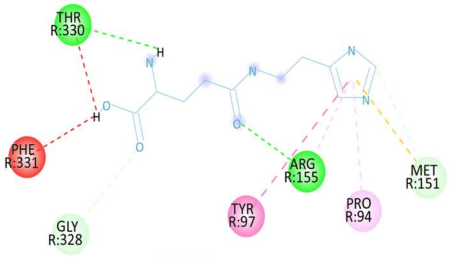
10.	(+)-alpha-Pinene (82227)	-5.2	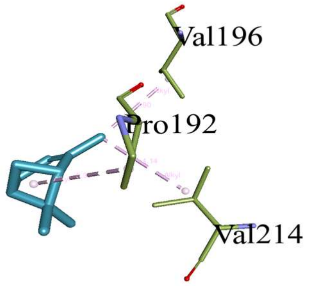	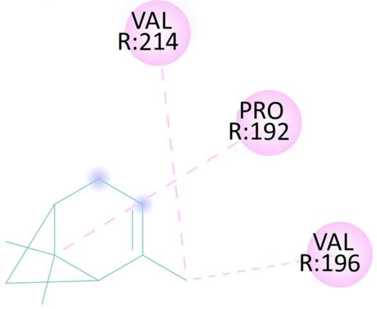
11.	Asparaginyl-Cysteine (18218178)	-5	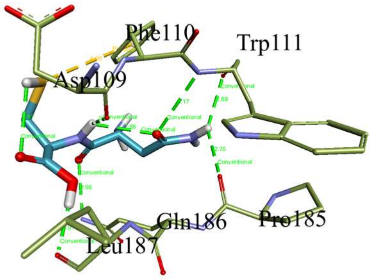	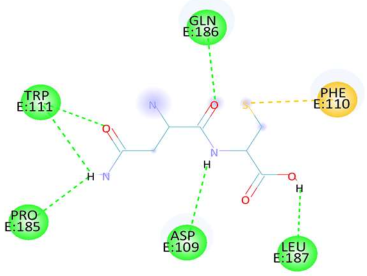
12.	Benzyl Isothiocyanate (2346)	-4.8	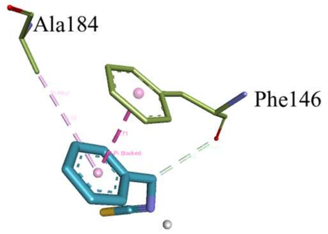	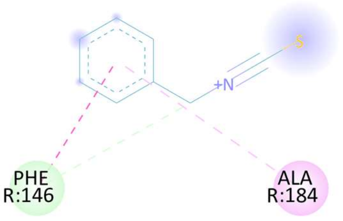
13.	3beta,6beta Dihydroxynortropane (22297531)	-4.6	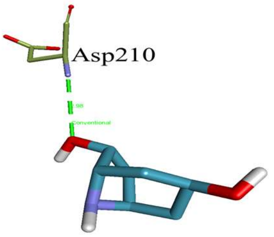	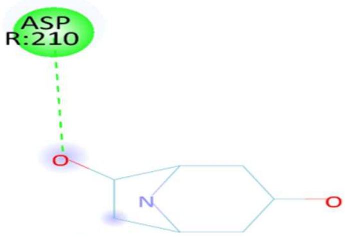
14.	Medicanine (101409750)	-4.2	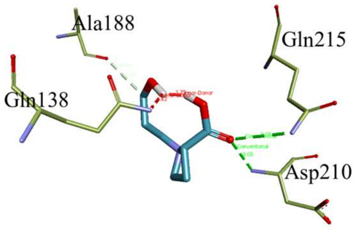	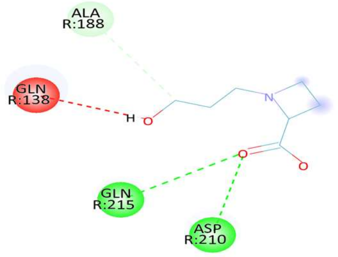

Table S3. Molecular docking and interaction studies of chemical compounds identified at Negative ion mode with therapeutic membrane receptors of breast cancer, *viz.* Estrogen Receptor *α* (ER*α* / ESR1; PDB ID: 1R5K), Progesterone receptor (PR; PDB ID: 4OAR), Insulin-Like Growth Factor-1 Receptor (IGF-1R; PDB ID: 1IGR), Epidermal Growth Factor Receptor (EGFR / ERBB1; PDB ID: 1IVO), Human Epidermal growth factor Receptor 3 (HER3; PDB ID: 3KEX), Membrane progesterone receptor alpha (mPR*α*; PDB identifier/UniProt entry Q86WK9) and G-Protein Coupled Estrogen Receptor (GPER/ GPR30; PDB ID: 8XOG) through PyRx docking tool.

**Molecular docking with Estrogen Receptor *α* (ER*α* / ESR1)**				
**S. No.**	**Compounds (PubChem CID)**	**Binding Affinity (Kcal/mol)**	**3D interaction**	**2D Interaction**
1.	Glucotropaeolin (9548605)	-7.6	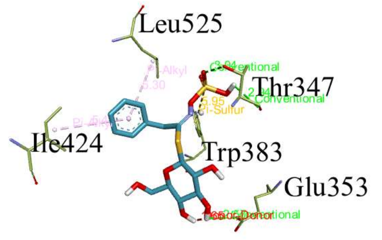	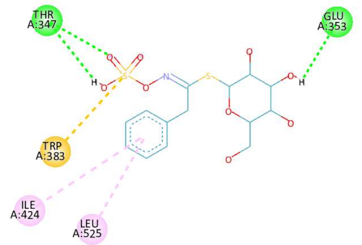
2.	8S-HODE (16061037)	-6.4	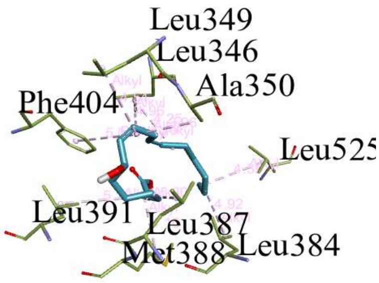	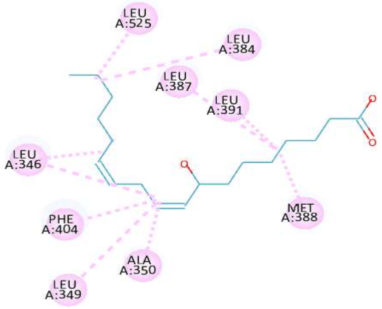
3.	3-Keto stearic acid (5283005)	-6.1	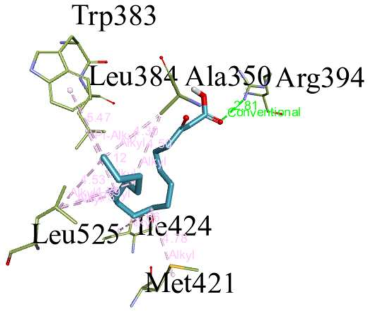	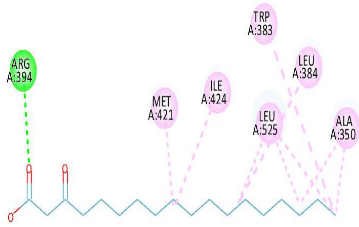
4.	16-Hydroxy hexadecanoic acid (10466)	-5.6	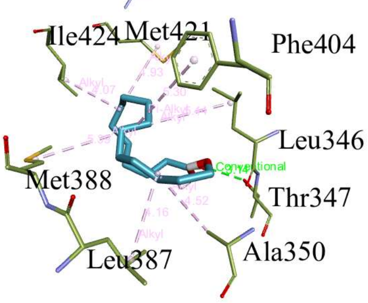	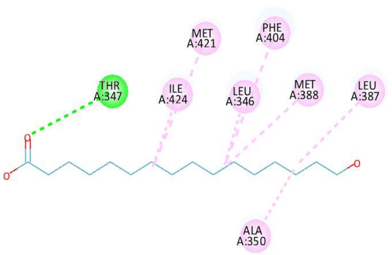

5.	Benzyl isothiocyanate (2346)	-5.3	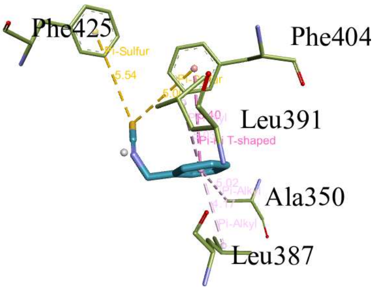	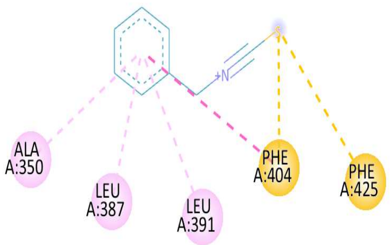
**Molecular docking with Progesterone receptor**				
1.	8S-HODE (16061037)	-6	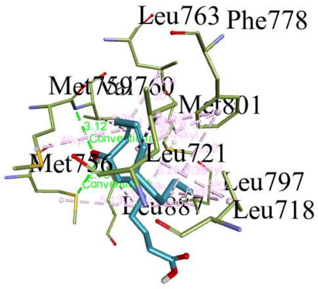	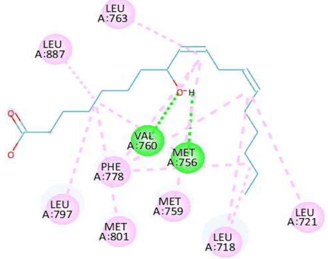
2.	16-Hydroxy hexadecanoic acid (10466)	-5.1	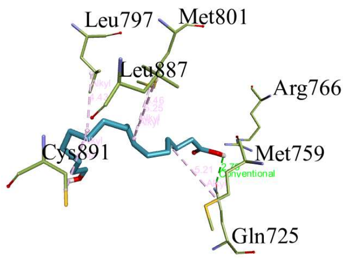	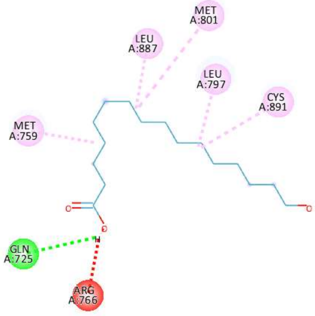
3.	3-Keto stearic acid (5283005)	-4.2	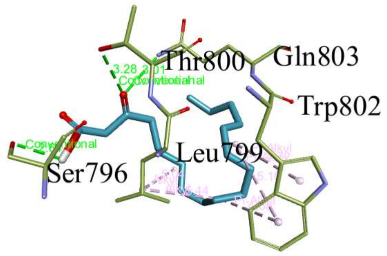	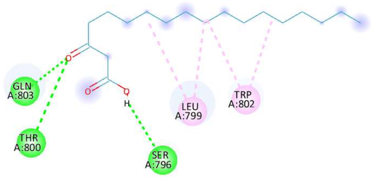
4.	Benzyl isothiocyanate (2346)	-4.1	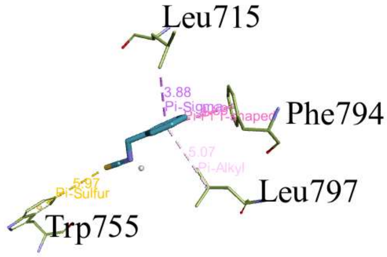	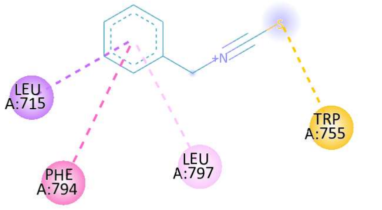
**Molecular docking with Insulin-Like Growth Factor-1 Receptor (IGF-1R)**				
1.	(-)-Epicatechin 3'-O-glucuronide (76969982)	-8.4	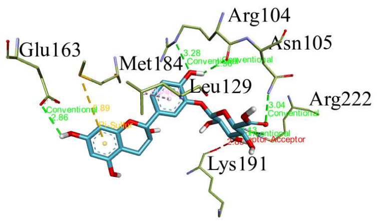	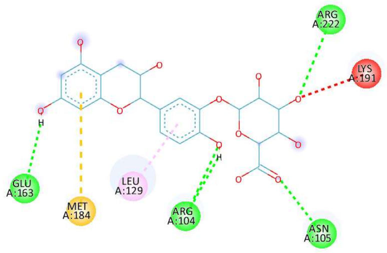

2.	Quercetin 3-(2-caffeoylglucuronoside) (131753131)	-8.1	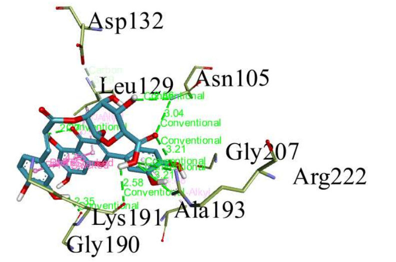	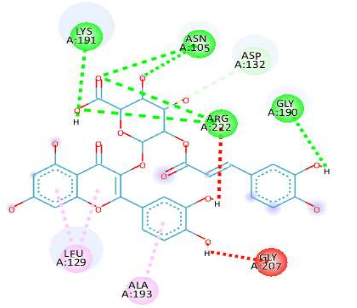
**Molecular docking with Epidermal Growth Factor Receptor (EGFR / ERBB1)**				
1.	Quercetin 3-(2-caffeoylglucuronoside) (131753131)	-9.5	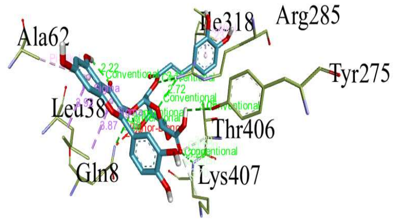	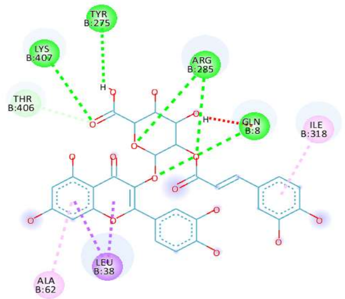
2.	(-)-Epicatechin 3'-O-glucuronide (76969982)	-9.5	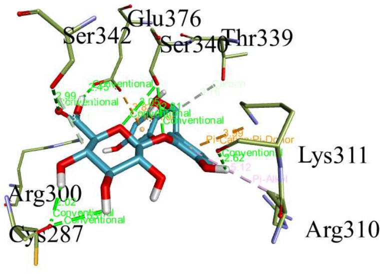	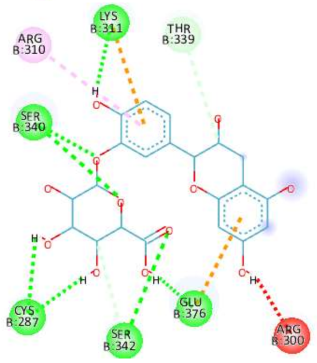
3.	Glucotropaeolin (9548605)	-8.1	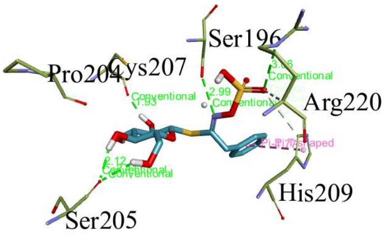	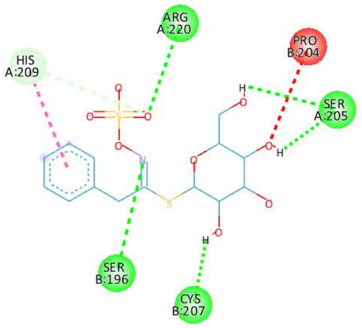
**Molecular docking with Human epidermal growth factor receptor 3 (HER3)**				
1.	Quercetin 3-(2-caffeoylglucuronoside) (131753131)	-9.8	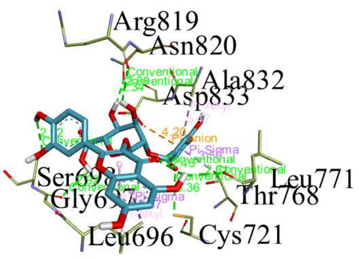	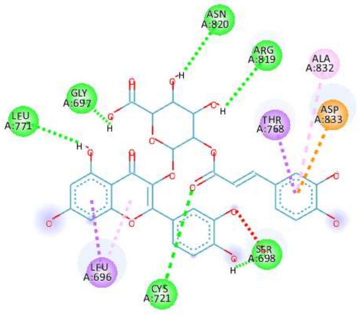

2.	(-)-Epicatechin 3'-O-glucuronide (76969982)	-8.8	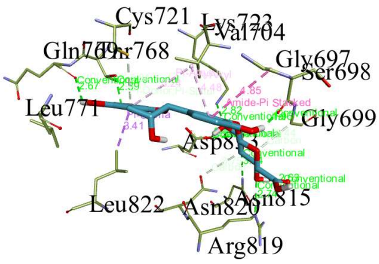	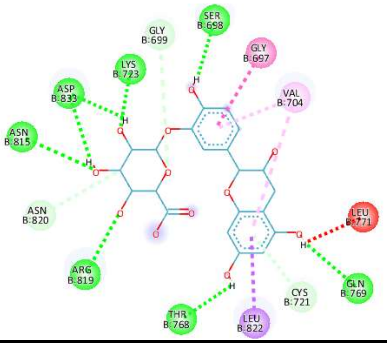
**Molecular docking with Membrane progesterone receptor alpha (mPRα)**				
1.	Quercetin 3-(2-caffeoylglucuronoside) (131753131)	-10.2	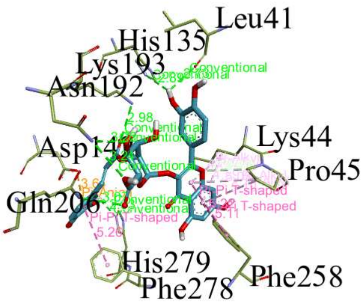	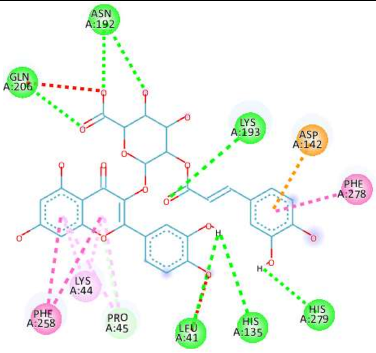
2.	(-)-Epicatechin 3'-O-glucuronide (76969982)	-8.8	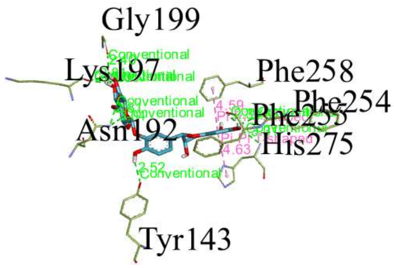	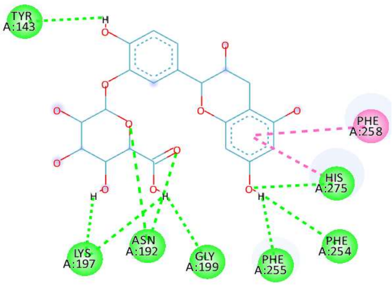
**Molecular docking with G-Protein Coupled Estrogen Receptor (GPER / GPR30)**				
1.	Quercetin 3-(2-caffeoylglucuronoside) (131753131)	-7.8	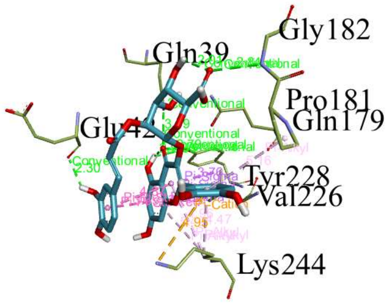	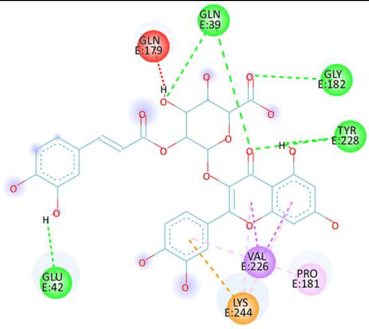
2.	(-)-Epicatechin 3'-O-glucuronide (76969982)	-7.7	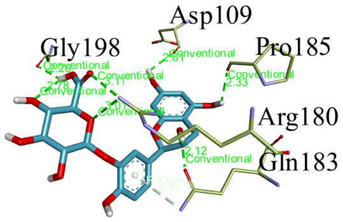	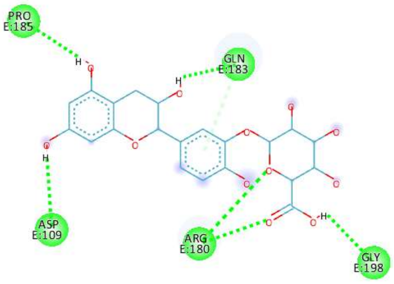

Table S4. Binding interaction studies of identified phytomolecules from *S. persica* root extract at positive-ESI mode against therapeutic membrane receptors of breast cancer, *viz.* Estrogen Receptor α (ERα / ESR1; PDB ID: 1R5K), Progesterone receptor (PR; PDB ID: 4OAR), Insulin-Like Growth Factor-1 Receptor (IGF-1R; PDB ID: 1IGR), Epidermal Growth Factor Receptor (EGFR / ERBB1; PDB ID: 1IVO), Human Epidermal growth factor Receptor 3 (HER3; PDB ID: 3KEX), Membrane progesterone receptor alpha (mPRα; PDB identifier/UniProt entry Q86WK9) and G-Protein Coupled Estrogen Receptor (GPER/ GPR30; PDB ID: 8XOG) through PyRx docking tool.

S. No.	Binding interaction with Estrogen Receptor α (ERα / ESR1)				
	**Phytochemicals**	**Interacting Amino acid residues**	**Bond Distance (Å)**	**Bond Category**	**Bond Types**
1.	Pipercitine (12575258)	LEU346	5.15433	Hydrophobic	Alkyl
		ALA350	4.22368	Hydrophobic	Alkyl
		ALA350	4.2604	Hydrophobic	Alkyl
		LEU387	5.03669	Hydrophobic	Alkyl
		LEU525	4.59462	Hydrophobic	Alkyl
		LEU384	5.13965	Hydrophobic	Alkyl
		LEU525	4.64746	Hydrophobic	Alkyl
		LEU525	4.14504	Hydrophobic	Alkyl
		LEU525	3.99426	Hydrophobic	Alkyl
		LYS529	4.29468	Hydrophobic	Alkyl
		PRO535	4.3284	Hydrophobic	Alkyl
		MET421	4.89362	Hydrophobic	Alkyl
		ILE424	4.10042	Hydrophobic	Alkyl
		PHE404	5.04604	Hydrophobic	Pi-Alkyl
		TYR537	5.03587	Hydrophobic	Pi-Alkyl
2.	p-Coumaroylagmatine (440362	LEU346	2.5703	Hydrogen Bond	Conventional Hydrogen Bond
		GLU353	2.32115	Hydrogen Bond	Conventional Hydrogen Bond
		GLU353	2.33067	Hydrogen Bond	Conventional Hydrogen Bond
		LEU387	2.17378	Hydrogen Bond	Conventional Hydrogen Bond
		THR347	4.10626	Hydrogen Bond	Pi-Donor Hydrogen Bond
		THR347	3.48861	Hydrophobic	Pi-Sigma
		ALA350	4.15756	Hydrophobic	Alkyl
		LEU346	5.39991	Hydrophobic	Alkyl
		LEU387	4.88846	Hydrophobic	Alkyl
		LEU525	4.55061	Hydrophobic	Pi-Alkyl
3.	Macamide B (11198769)	PHE404	5.03623	Hydrophobic	Pi-Pi T-shaped
		ALA350	4.09046	Hydrophobic	Alkyl
		LEU525	4.77013	Hydrophobic	Alkyl
		LEU525	4.93232	Hydrophobic	Alkyl
		LEU525	4.95128	Hydrophobic	Alkyl
		LEU525	4.05145	Hydrophobic	Alkyl
		LYS529	3.85158	Hydrophobic	Alkyl
		TYR537	4.71902	Hydrophobic	Pi-Alkyl
		LEU346	5.20089	Hydrophobic	Pi-Alkyl
		LEU349	5.41688	Hydrophobic	Pi-Alkyl
		ALA350	4.157	Hydrophobic	Pi-Alkyl
		LEU387	4.98885	Hydrophobic	Pi-Alkyl

4.	Benzoxazinone glucoside (77195081)	THR347	1.93051	Hydrogen Bond	Conventional Hydrogen Bond
		THR347	3.04438	Hydrogen Bond	Carbon Hydrogen Bond
		LEU346	3.47677	Hydrogen Bond	Carbon Hydrogen Bond
		GLU353	3.46877	Hydrogen Bond	Carbon Hydrogen Bond
		LEU387	3.40947	Hydrogen Bond	Carbon Hydrogen Bond
		ALA350	4.42152	Hydrophobic	Alkyl
		LEU346	4.27492	Hydrophobic	Alkyl
		LEU349	3.91389	Hydrophobic	Alkyl
		LEU387	4.22548	Hydrophobic	Alkyl
		LEU391	4.53243	Hydrophobic	Alkyl
		PHE404	4.65094	Hydrophobic	Pi-Alkyl
		ALA350	5.16295	Hydrophobic	Pi-Alkyl
		LEU387	4.86256	Hydrophobic	Pi-Alkyl
		MET388	5.40888	Hydrophobic	Pi-Alkyl
5.	N-(14-Methylhexadecanoyl)pyrrolidine (6430518)	LEU346	5.45928	Hydrophobic	Alkyl
		LEU346	5.34205	Hydrophobic	Alkyl
		LEU349	4.87643	Hydrophobic	Alkyl
		ALA350	3.98971	Hydrophobic	Alkyl
		ALA350	4.19337	Hydrophobic	Alkyl
		LEU387	5.0034	Hydrophobic	Alkyl
		LEU525	4.65677	Hydrophobic	Alkyl
		MET421	4.79451	Hydrophobic	Alkyl
		ILE424	3.8647	Hydrophobic	Alkyl
		LEU384	5.3031	Hydrophobic	Alkyl
		LEU525	4.20925	Hydrophobic	Alkyl
		PHE404	5.0053	Hydrophobic	Pi-Alkyl
		PHE404	4.99708	Hydrophobic	Pi-Alkyl
6.	N-Acetyl-leucyl-leucin (443129)	THR347	2.84978	Hydrogen Bond	Conventional Hydrogen Bond
		LEU346	2.70992	Hydrogen Bond	Conventional Hydrogen Bond
		LEU384	5.49654	Hydrophobic	Alkyl
		LEU525	4.74411	Hydrophobic	Alkyl
		LEU387	4.41653	Hydrophobic	Alkyl
		MET388	4.80303	Hydrophobic	Alkyl
		LEU391	4.87601	Hydrophobic	Alkyl
7.	5-Methoxydimethyltryptamine (1832)	LEU346	2.45892	Hydrogen Bond	Conventional Hydrogen Bond
		GLU353	3.55244	Hydrogen Bond	Carbon Hydrogen Bond
		LEU387	3.01941	Hydrogen Bond	Carbon Hydrogen Bond
		PHE404	4.88985	Hydrophobic	Pi-Pi T-shaped
		LEU387	4.32737	Hydrophobic	Alkyl
		LEU391	4.68879	Hydrophobic	Alkyl
		LEU346	4.81119	Hydrophobic	Pi-Alkyl
		ALA350	4.10721	Hydrophobic	Pi-Alkyl
		LEU346	5.42311	Hydrophobic	Pi-Alkyl
		LEU349	5.18701	Hydrophobic	Pi-Alkyl
		ALA350	4.39881	Hydrophobic	Pi-Alkyl
		LEU387	4.94341	Hydrophobic	Pi-Alkyl
8.	Leucyl-Histidine (6992828)	LEU346	2.27571	Hydrogen Bond	Conventional Hydrogen Bond
		GLY521	2.59811	Hydrogen Bond	Conventional Hydrogen Bond
		MET421	5.0588	Other	Pi-Sulfur
		ALA350	5.23067	Hydrophobic	Alkyl
		LEU387	4.42103	Hydrophobic	Alkyl

		LEU391<br>PHE404<br>ILE424<br>LEU525	4.76599<br>5.10519<br>4.8717<br>4.74516	Hydrophobic<br>Hydrophobic<br>Hydrophobic<br>Hydrophobic	Alkyl<br>Pi-Alkyl<br>Pi-Alkyl<br>Pi-Alkyl
9.	Asparaginyl-Cysteine (18218178)	ARG394<br>LYS449<br>GLU353<br>GLU353	3.20257<br>2.83971<br>2.07219<br>1.99155	Hydrogen Bond<br>Hydrogen Bond<br>Hydrogen Bond<br>Hydrogen Bond	Conventional Hydrogen Bond<br>Conventional Hydrogen Bond<br>Conventional Hydrogen Bond<br>Conventional Hydrogen Bond
10.	N(alpha)-gamma-Lglutamylhistamine (440238)	ARG394<br>LEU387<br>LEU525<br>LEU384	2.88198<br>2.27577<br>3.68357<br>5.27402	Hydrogen Bond<br>Hydrogen Bond<br>Hydrophobic<br>Hydrophobic	Conventional Hydrogen Bond<br>Conventional Hydrogen Bond<br>Pi-Sigma<br>Pi-Alkyl
11.	Europine (5462451)	SER512<br>TYR459<br>LEU508<br>THR483	3.09254<br>2.18413<br>2.60598<br>3.70478	Hydrogen Bond<br>Hydrogen Bond<br>Hydrogen Bond<br>Hydrogen Bond	Conventional Hydrogen Bond<br>Conventional Hydrogen Bond<br>Conventional Hydrogen Bond<br>Carbon Hydrogen Bond
12.	(+)-alpha-Pinene (82227)	ALA350<br>ALA350<br>ALA350<br>LEU387<br>LEU346<br>LEU384	4.43791<br>3.84547<br>3.91671<br>4.42591<br>4.54583<br>4.85387	Hydrophobic<br>Hydrophobic<br>Hydrophobic<br>Hydrophobic<br>Hydrophobic<br>Hydrophobic	Alkyl<br>Alkyl<br>Alkyl<br>Alkyl<br>Alkyl<br>Alkyl
13.	Benzyl isothiocyanate (2346)	PHE404<br>ALA350<br>LEU387<br>LEU391	5.36561<br>4.99736<br>4.22363<br>5.059	Hydrophobic<br>Hydrophobic<br>Hydrophobic<br>Hydrophobic	Pi-Pi T-shaped<br>Pi-Alkyl<br>Pi-Alkyl<br>Pi-Alkyl
14.	3beta,6betaDihydroxynortropane (22297531)	GLU353<br>GLU353	1.9754<br>2.20985	Hydrogen Bond<br>Hydrogen Bond	Conventional Hydrogen Bond<br>Conventional Hydrogen Bond
15.	Medicanine (101409750)	MET357<br>ARG394<br>GLU323<br>GLY390	3.12375<br>3.07126<br>2.02336<br>3.55502	Hydrogen Bond<br>Hydrogen Bond<br>Hydrogen Bond<br>Hydrogen Bond	Conventional Hydrogen Bond<br>Conventional Hydrogen Bond<br>Conventional Hydrogen Bond<br>Carbon Hydrogen Bond
**Binding interaction of phytochemicals with progesterone receptor**					
1.	Benzoxazinone glucoside (77195081)	VAL698<br>GLN815<br>ASP697<br>GLY762<br>TRP765<br>ARG766<br>ARG766<br>PRO696<br>LYS822<br>VAL729<br>MET759<br>PRO696	3.12416<br>3.37532<br>2.22154<br>3.7584<br>3.13265<br>3.51314<br>4.13116<br>4.9168<br>5.02375<br>5.12341<br>4.01476<br>4.1453	Hydrogen Bond<br>Hydrogen Bond<br>Hydrogen Bond<br>Hydrogen Bond<br>Hydrogen Bond<br>Hydrogen Bond<br>Electrostatic<br>Hydrophobic<br>Hydrophobic<br>Hydrophobic<br>Hydrophobic<br>Hydrophobic	Conventional Hydrogen Bond<br>Conventional Hydrogen Bond<br>Conventional Hydrogen Bond<br>Carbon Hydrogen Bond<br>Carbon Hydrogen Bond<br>Carbon Hydrogen Bond<br>Pi-Cation<br>Alkyl<br>Alkyl<br>Alkyl<br>Alkyl<br>Pi-Alkyl
2.	Europine (5462451)	ARG766<br>LYS822<br>GLY762<br>GLU695	3.05569<br>3.24719<br>3.20986<br>3.26955	Hydrogen Bond<br>Hydrogen Bond<br>Hydrogen Bond<br>Hydrogen Bond	Conventional Hydrogen Bond<br>Conventional Hydrogen Bond<br>Carbon Hydrogen Bond<br>Carbon Hydrogen Bond

		TRP732	3.93661	Hydrophobic	Pi-Sigma
3.	Leucyl-Histidine (6992828)	SER728<br>GLN725<br>LEU758<br>LYS822<br>VAL729<br>TRP732<br>TRP732<br>VAL698<br>PRO696<br>LEU758<br>LYS822	3.16291<br>2.24479<br>2.37444<br>3.44746<br>3.9352<br>5.29881<br>4.98634<br>4.38467<br>5.33049<br>4.618<br>5.30032	Hydrogen Bond<br>Hydrogen Bond<br>Hydrogen Bond<br>Hydrogen Bond;<br>Electrostatic<br>Hydrophobic<br>Hydrophobic<br>Hydrophobic<br>Hydrophobic<br>Hydrophobic<br>Hydrophobic<br>Hydrophobic	Conventional Hydrogen Bond<br>Conventional Hydrogen Bond<br>Conventional Hydrogen Bond<br>Pi-Cation; Pi-Donor Hydrogen<br>Bond<br>Pi-Sigma<br>Pi-Pi T-shaped<br>Pi-Pi T-shaped<br>Alkyl<br>Pi-Alkyl<br>Pi-Alkyl<br>Pi-Alkyl
4.	Macamide B (11198769)	SER728<br>GLN725<br>VAL729<br>TRP732<br>PRO696<br>PRO696<br>VAL698<br>VAL698<br>ARG766<br>TRP765<br>PRO696<br>LEU758	3.14657<br>3.75792<br>3.46104<br>5.34004<br>5.2811<br>4.95<br>5.12387<br>5.44614<br>5.22293<br>4.97091<br>5.03888<br>5.05881	Hydrogen Bond<br>Hydrogen Bond<br>Hydrophobic<br>Hydrophobic<br>Hydrophobic<br>Hydrophobic<br>Hydrophobic<br>Hydrophobic<br>Hydrophobic<br>Hydrophobic<br>Hydrophobic<br>Hydrophobic	Conventional Hydrogen Bond<br>Carbon Hydrogen Bond<br>Pi-Sigma<br>Pi-Pi T-shaped<br>Alkyl<br>Alkyl<br>Alkyl<br>Alkyl<br>Alkyl<br>Pi-Alkyl<br>Pi-Alkyl<br>Pi-Alkyl
5.	p-Coumaroylagmatine (440362)	ILE699<br>PRO696<br>GLU695<br>GLU695<br>ARG766<br>VAL698<br>ARG766<br>PRO696	3.08012<br>2.9837<br>2.25064<br>2.16487<br>4.27157<br>4.97084<br>5.0069<br>5.18581	Hydrogen Bond<br>Hydrogen Bond<br>Hydrogen Bond<br>Hydrogen Bond<br>Electrostatic<br>Hydrophobic<br>Hydrophobic<br>Hydrophobic	Conventional Hydrogen Bond<br>Conventional Hydrogen Bond<br>Conventional Hydrogen Bond<br>Conventional Hydrogen Bond<br>Pi-Cation<br>Alkyl<br>Alkyl<br>Pi-Alkyl
6.	N-Acetyl-leucyl-leucin (443129)	ARG766<br>LYS822<br>GLU695<br>GLU695<br>PRO696<br>VAL698	3.13547<br>3.18767<br>2.52532<br>2.85059<br>4.35771<br>4.96518	Hydrogen Bond<br>Hydrogen Bond<br>Hydrogen Bond<br>Hydrogen Bond<br>Hydrophobic<br>Hydrophobic	Conventional Hydrogen Bond<br>Conventional Hydrogen Bond<br>Conventional Hydrogen Bond<br>Conventional Hydrogen Bond<br>Alkyl<br>Alkyl
7.	N-(14-Methylhexadecanoyl)pyrrolidine (6430518)	SER728<br>PRO696<br>PRO696<br>VAL698<br>VAL698<br>ILE699<br>VAL729<br>ARG766<br>ARG766<br>PRO780<br>TRP732<br>TRP732	3.54146<br>5.01061<br>4.50072<br>4.02954<br>4.36678<br>4.40134<br>4.27552<br>4.8161<br>5.07053<br>4.98389<br>4.98199<br>5.07077	Hydrogen Bond<br>Hydrophobic<br>Hydrophobic<br>Hydrophobic<br>Hydrophobic<br>Hydrophobic<br>Hydrophobic<br>Hydrophobic<br>Hydrophobic<br>Hydrophobic<br>Hydrophobic<br>Hydrophobic	Carbon Hydrogen Bond<br>Alkyl<br>Alkyl<br>Alkyl<br>Alkyl<br>Alkyl<br>Alkyl<br>Alkyl<br>Alkyl<br>Alkyl<br>Pi-Alkyl<br>Pi-Alkyl

		TRP765<br>HIS770	4.78862<br>4.75113	Hydrophobic<br>Hydrophobic	Pi-Alkyl<br>Pi-Alkyl
8.	(+)-alpha-Pinene (82227)	TYR753<br>VAL884<br>LYS885<br>VAL925<br>VAL884<br>HIS881	3.99652<br>4.46791<br>5.20066<br>4.81849<br>4.63933<br>4.99702	Hydrophobic<br>Hydrophobic<br>Hydrophobic<br>Hydrophobic<br>Hydrophobic<br>Hydrophobic	Pi-Sigma<br>Alkyl<br>Alkyl<br>Alkyl<br>Alkyl<br>Pi-Alkyl
9.	5-Methoxydimethyltryptamine (1832)	ILE699<br>GLN725<br>HIS770<br>ARG766<br>ARG766<br>ILE699<br>ILE699<br>VAL698<br>ASP697,<br>VAL698<br>ASP697,<br>VAL698<br>ARG766<br>HIS770<br>ILE699<br>ARG766	2.41239<br>3.24765<br>3.76309<br>3.97269<br>4.56957<br>3.51612<br>3.8286<br>3.71265<br>5.044<br>4.67478<br>4.65242<br>4.89075<br>4.38305<br>5.16881	Hydrogen Bond<br>Hydrogen Bond<br>Hydrogen Bond<br>Electrostatic<br>Electrostatic<br>Hydrogen Bond<br>Hydrogen Bond<br>Hydrophobic<br>Hydrophobic<br>Hydrophobic<br>Hydrophobic<br>Hydrophobic<br>Hydrophobic<br>Hydrophobic	Conventional Hydrogen Bond<br>Carbon Hydrogen Bond<br>Carbon Hydrogen Bond<br>Pi-Cation<br>Pi-Cation<br>Pi-Donor Hydrogen Bond<br>Pi-Donor Hydrogen Bond<br>Pi-Sigma<br>Amide-Pi Stacked<br>Amide-Pi Stacked<br>Alkyl<br>Pi-Alkyl<br>Pi-Alkyl<br>Pi-Alkyl
10.	3beta,6betaDihydroxynortropane (22297531)	LYS885<br>HIS888<br>PRO918	3.12867<br>2.72424<br>2.08339	Hydrogen Bond<br>Hydrogen Bond<br>Hydrogen Bond	Conventional Hydrogen Bond<br>Conventional Hydrogen Bond<br>Conventional Hydrogen Bond
11.	N(alpha)-gamma-Lglutamylhistamine (440238)	ARG766<br>PRO696<br>GLN725<br>LYS822<br>PRO696<br>LEU758<br>LYS822	3.32261<br>2.32366<br>3.5922<br>3.81949<br>5.31885<br>5.35485<br>5.42856	Hydrogen Bond<br>Hydrogen Bond<br>Hydrogen Bond<br>Hydrogen Bond;<br>Electrostatic<br>Hydrophobic<br>Hydrophobic<br>Hydrophobic	Conventional Hydrogen Bond<br>Conventional Hydrogen Bond<br>Carbon Hydrogen Bond<br>Pi-Cation; Pi-Donor Hydrogen<br>Bond<br>Pi-Alkyl<br>Pi-Alkyl<br>Pi-Alkyl
12.	Pipercitine (12575258)	LEU715<br>CYS891<br>CYS891<br>CYS891<br>PHE895	4.62456<br>5.07984<br>4.89486<br>5.15812<br>4.61749	Hydrophobic<br>Hydrophobic<br>Hydrophobic<br>Hydrophobic<br>Hydrophobic	Alkyl<br>Alkyl<br>Alkyl<br>Alkyl<br>Pi-Alkyl
13.	Medicanine (101409750)	ARG766<br>ARG766<br>LYS822<br>LEU758	3.02361<br>3.08391<br>2.98055<br>2.09384	Hydrogen Bond<br>Hydrogen Bond<br>Hydrogen Bond<br>Hydrogen Bond	Conventional Hydrogen Bond<br>Conventional Hydrogen Bond<br>Conventional Hydrogen Bond<br>Conventional Hydrogen Bond
14.	Asparaginyl-Cysteine (18218178)	ARG766<br>ARG766<br>LYS822<br>LEU758<br>ILE699<br>ASP697<br>ARG766	3.22208<br>3.38205<br>3.11167<br>2.75611<br>2.77715<br>2.77958<br>3.74363	Hydrogen Bond<br>Hydrogen Bond<br>Hydrogen Bond<br>Hydrogen Bond<br>Hydrogen Bond<br>Hydrogen Bond<br>Hydrogen Bond	Conventional Hydrogen Bond<br>Conventional Hydrogen Bond<br>Conventional Hydrogen Bond<br>Conventional Hydrogen Bond<br>Conventional Hydrogen Bond<br>Conventional Hydrogen Bond<br>Carbon Hydrogen Bond

15.	Benzyl	GLN725	3.53749	Hydrogen Bond	Pi-Donor Hydrogen Bond
	isothiocyanate	PHE778	5.04056	Hydrophobic	Pi-Pi T-shaped
	(2346)	MET759	5.11396	Hydrophobic	Pi-Alkyl
		LEU763	4.97951	Hydrophobic	Pi-Alkyl
**Binding interaction of phytochemicals with Insulin-Like Growth Factor-1 Receptor (IGF-1R)**					
1.	Benzoxazinone	ARG104	3.28919	Hydrogen Bond	Conventional Hydrogen Bond
	glucoside	ARG104	2.80254	Hydrogen Bond	Conventional Hydrogen Bond
	(77195081)	ASN105	3.08433	Hydrogen Bond	Conventional Hydrogen Bond
		ARG222	3.19493	Hydrogen Bond	Conventional Hydrogen Bond
		LEU129	2.57999	Hydrogen Bond	Conventional Hydrogen Bond
		GLY190	3.49617	Hydrogen Bond	Carbon Hydrogen Bond
		LEU129	3.81977	Hydrophobic	Pi-Sigma
2.	p-	LEU471	2.79623	Hydrogen Bond	Conventional Hydrogen Bond
	Coumaroylag	GLU454	2.17564	Hydrogen Bond	Conventional Hydrogen Bond
	matine	LEU471	2.84079	Hydrogen Bond	Conventional Hydrogen Bond
	(440362)	ASP476	2.24092	Hydrogen Bond	Conventional Hydrogen Bond
		PHE420	4.29961	Hydrophobic	Pi-Pi Stacked
		PRO422	4.8281	Hydrophobic	Alkyl
		PHE420	5.41752	Hydrophobic	Pi-Alkyl
3.	Leucyl-	ILE255	2.88591	Hydrogen Bond	Conventional Hydrogen Bond
	Histidine	LEU256	3.00731	Hydrogen Bond	Conventional Hydrogen Bond
	(6992828)	SER261	2.35198	Hydrogen Bond	Conventional Hydrogen Bond
		ALA253	2.07271	Hydrogen Bond	Conventional Hydrogen Bond
		ALA253	2.89179	Hydrogen Bond	Conventional Hydrogen Bond
		PHE266	3.54196	Hydrogen Bond	Carbon Hydrogen Bond
		ALA253	4.52014	Hydrophobic	Pi-Alkyl
		MET289	4.631	Hydrophobic	Pi-Alkyl
4.	5-	ARG192	3.29017	Hydrogen Bond	Carbon Hydrogen Bond
	Methoxydimet	LYS191	3.73206	Hydrogen Bond	Carbon Hydrogen Bond
	hyltryptamine	ARG104	3.771	Hydrogen Bond	Carbon Hydrogen Bond
	(1832)	LYS183	3.56971	Hydrogen Bond	Carbon Hydrogen Bond
		ARG104	4.38651	Electrostatic	Pi-Cation
		ARG104	3.71146	Hydrogen Bond;	Pi-Cation; Pi-Donor Hydrogen
		CYS185	4.32421	Electrostatic	Bond
		LEU129,	4.8053	Other	Pi-Sulfur
		ILE130	4.54678	Hydrophobic	Amide-Pi Stacked
		ARG104	4.72143	Hydrophobic	Alkyl
		LEU129	4.41776	Hydrophobic	Alkyl
		ILE130	4.77449	Hydrophobic	Alkyl
		LEU129	5.32607	Hydrophobic	Pi-Alkyl
		CYS185		Hydrophobic	Pi-Alkyl
5.	Europine	ASP132	3.65281	Hydrogen Bond	Carbon Hydrogen Bond
	(5462451)	SER128	3.46302	Hydrogen Bond	Carbon Hydrogen Bond
6.	N(alpha)-	ARG104	3.04269	Hydrogen Bond	Conventional Hydrogen Bond
	gamma-	ASN105	3.21414	Hydrogen Bond	Conventional Hydrogen Bond
	Lglutamylhista	LYS191	2.13448	Hydrogen Bond	Conventional Hydrogen Bond
	mine (440238)	ARG104	2.09531	Hydrogen Bond	Conventional Hydrogen Bond
		LEU129	2.6714	Hydrogen Bond	Conventional Hydrogen Bond
		GLY190	2.48051	Hydrogen Bond	Conventional Hydrogen Bond
		ARG192	2.69659	Hydrogen Bond	Conventional Hydrogen Bond
		GLY207	3.58476	Hydrogen Bond	Carbon Hydrogen Bond

		CYS185	5.70989	Other	Pi-Sulfur
7.	N-Acetyl-leucyl-leucin (443129)	ILE255<br>SER257<br>ALA253<br>ALA253<br>ALA253<br>MET289	3.05384<br>3.37658<br>3.06597<br>2.49567<br>3.6824<br>4.75366	Hydrogen Bond<br>Hydrogen Bond<br>Hydrogen Bond<br>Hydrogen Bond<br>Hydrophobic<br>Hydrophobic	Conventional Hydrogen Bond<br>Conventional Hydrogen Bond<br>Conventional Hydrogen Bond<br>Conventional Hydrogen Bond<br>Alkyl<br>Alkyl
8.	(+)-alpha-Pinene (82227)	PHE58<br>LEU32<br>LEU56N<br>PHE58<br>PHE58<br>PHE82	3.85562<br>4.80665<br>4.73683<br>3.77678<br>4.29423<br>4.86256	Hydrophobic<br>Hydrophobic<br>Hydrophobic<br>Hydrophobic<br>Hydrophobic<br>Hydrophobic	Pi-Sigma<br>Alkyl<br>Alkyl<br>Pi-Alkyl<br>Pi-Alkyl<br>Pi-Alkyl
9.	Macamide B (11198769)	VAL134<br>ILE167<br>LEU122<br>ILE139<br>ILE139<br>ILE167	3.99125<br>3.73234<br>5.03114<br>3.85837<br>4.68603<br>4.41736	Hydrophobic<br>Hydrophobic<br>Hydrophobic<br>Hydrophobic<br>Hydrophobic<br>Hydrophobic	Pi-Sigma<br>Pi-Sigma<br>Alkyl<br>Alkyl<br>Alkyl<br>Alkyl
10.	N-(14-Methylhexadecanoyl)pyrrolidine (6430518)	PHE90<br>LEU56<br>LEU32<br>LEU56<br>HIS30<br>PHE58<br>PHE58<br>PHE58<br>PHE82<br>PHE82	3.64589<br>4.92347<br>4.21268<br>4.45722<br>4.53961<br>5.42088<br>3.77629<br>5.09391<br>5.09841<br>4.97184	Hydrophobic<br>Hydrophobic<br>Hydrophobic<br>Hydrophobic<br>Hydrophobic<br>Hydrophobic<br>Hydrophobic<br>Hydrophobic<br>Hydrophobic<br>Hydrophobic	Pi-Sigma<br>Alkyl<br>Alkyl<br>Alkyl<br>Pi-Alkyl<br>Pi-Alkyl<br>Pi-Alkyl<br>Pi-Alkyl<br>Pi-Alkyl<br>Pi-Alkyl
11.	Medicanine (101409750)	ILE255<br>LEU256<br>GLN287<br>ALA253<br>GLN287<br>PHE266	3.11274<br>3.03875<br>2.12128<br>3.00295<br>3.78833<br>3.34223	Hydrogen Bond<br>Hydrogen Bond<br>Hydrogen Bond<br>Hydrogen Bond<br>Hydrogen Bond<br>Hydrogen Bond	Conventional Hydrogen Bond<br>Conventional Hydrogen Bond<br>Conventional Hydrogen Bond<br>Conventional Hydrogen Bond<br>Carbon Hydrogen Bond<br>Carbon Hydrogen Bond
12.	3beta,6betaDihydroxynortropane (22297531)	ARG104<br>ARG104<br>GLY207<br>ARG104	3.10748<br>3.04923<br>3.11462<br>3.21386	Hydrogen Bond<br>Hydrogen Bond<br>Hydrogen Bond<br>Hydrogen Bond	Conventional Hydrogen Bond<br>Conventional Hydrogen Bond<br>Conventional Hydrogen Bond<br>Carbon Hydrogen Bond
13.	Asparaginyl-Cysteine (18218178)	ASN105<br>ASN105<br>LEU129<br>LEU129<br>SER128	2.93781<br>3.18764<br>2.20274<br>1.9966<br>2.56157	Hydrogen Bond<br>Hydrogen Bond<br>Hydrogen Bond<br>Hydrogen Bond<br>Hydrogen Bond	Conventional Hydrogen Bond<br>Conventional Hydrogen Bond<br>Conventional Hydrogen Bond<br>Conventional Hydrogen Bond<br>Conventional Hydrogen Bond
14.	Pipercitine (12575258)	TRP127<br>VAL134<br>ILE139<br>PRO145<br>PRO145<br>ILE167<br>ILE139	3.70931<br>4.14949<br>4.84185<br>5.30657<br>5.0167<br>4.22822<br>4.6439	Hydrogen Bond<br>Hydrophobic<br>Hydrophobic<br>Hydrophobic<br>Hydrophobic<br>Hydrophobic<br>Hydrophobic	Carbon Hydrogen Bond<br>Alkyl<br>Alkyl<br>Alkyl<br>Alkyl<br>Alkyl<br>Alkyl

		LEU122<br>ILE139	3.96425<br>5.36536	Hydrophobic<br>Hydrophobic	Alkyl<br>Alkyl
15.	Benzyl isothiocyanate (2356)	GLU345<br>ASN346<br>LEU350<br>ASN346, PHE347	3.84283<br>3.74758<br>3.9388<br>3.69804	Electrostatic<br>Hydrogen Bond<br>Hydrophobic<br>Hydrophobic	Attractive Charge<br>Conventional Hydrogen Bond<br>Pi-Sigma<br>Amide-Pi Stacked
**Binding interaction of phytochemicals with Epidermal Growth Factor Receptor (EGFR / ERBB1)**					
1.	Benzoxazinone glucoside (77195081)	SER196<br>ARG220<br>SER205<br>PRO204<br>PRO219<br>HIS209<br>HIS209<br>HIS209	2.89908<br>3.02772<br>2.90991<br>2.04852<br>3.60314<br>5.50627<br>5.26464<br>4.18245	Hydrogen Bond<br>Hydrogen Bond<br>Hydrogen Bond<br>Hydrogen Bond<br>Hydrogen Bond<br>Hydrophobic<br>Hydrophobic<br>Hydrophobic	Conventional Hydrogen Bond<br>Conventional Hydrogen Bond<br>Conventional Hydrogen Bond<br>Conventional Hydrogen Bond<br>Carbon Hydrogen Bond<br>Pi-Pi T-shaped<br>Pi-Pi T-shaped<br>Pi-Alkyl
2.	p-Coumaroylagmatine (440362)	ARG220<br>SER196<br>CYS207<br>PRO219<br>SER205<br>SER205<br>SER205<br>HIS209<br>HIS209<br>PRO219	3.14618<br>2.6096<br>2.58341<br>2.71723<br>2.05622<br>2.42155<br>3.27505<br>3.62699<br>3.77097<br>4.87489	Hydrogen Bond<br>Hydrogen Bond<br>Hydrogen Bond<br>Hydrogen Bond<br>Hydrogen Bond<br>Hydrogen Bond<br>Hydrogen Bond<br>Hydrogen Bond<br>Hydrophobic<br>Hydrophobic	Conventional Hydrogen Bond<br>Conventional Hydrogen Bond<br>Conventional Hydrogen Bond<br>Conventional Hydrogen Bond<br>Conventional Hydrogen Bond<br>Conventional Hydrogen Bond<br>Carbon Hydrogen Bond<br>Carbon Hydrogen Bond<br>Pi-Sigma<br>Pi-Alkyl
3.	Leucyl-Histidine (6992828)	ARG310<br>SER340<br>SER340<br>SER342<br>TYR292<br>VAL312<br>GLU376<br>GLU376<br>LYS311<br>SER340	5.02446<br>3.73014<br>2.96108<br>2.9656<br>5.29149<br>3.46111<br>2.99793<br>2.99695<br>2.76403<br>2.0116	Hydrophobic<br>Hydrogen Bond<br>Hydrogen Bond<br>Hydrogen Bond<br>Hydrophobic<br>Hydrophobic<br>Hydrogen Bond<br>Hydrogen Bond<br>Hydrogen Bond<br>Hydrogen Bond	Alkyl<br>Carbon Hydrogen Bond<br>Conventional Hydrogen Bond<br>Conventional Hydrogen Bond<br>Pi-Alkyl<br>Pi-Sigma<br>Conventional Hydrogen Bond<br>Conventional Hydrogen Bond<br>Conventional Hydrogen Bond<br>Conventional Hydrogen Bond
4.	N-Acetyl-leucyl-leucin (443129)	SER291<br>THR339<br>SER340<br>VAL312<br>TYR292	3.08418<br>3.21389<br>2.99977<br>4.07798<br>5.47829	Hydrogen Bond<br>Hydrogen Bond<br>Hydrogen Bond<br>Hydrophobic<br>Hydrophobic	Conventional Hydrogen Bond<br>Conventional Hydrogen Bond<br>Conventional Hydrogen Bond<br>Alkyl<br>Pi-Alkyl
5.	Europine (5462451)	SER340<br>SER340<br>SER340<br>LYS311<br>SER291<br>LYS311<br>GLU293<br>SER342	2.90504<br>2.95503<br>2.79002<br>2.48089<br>2.04143<br>3.51369<br>3.47356<br>3.51737	Hydrogen Bond<br>Hydrogen Bond<br>Hydrogen Bond<br>Hydrogen Bond<br>Hydrogen Bond<br>Hydrogen Bond<br>Hydrogen Bond<br>Hydrogen Bond	Conventional Hydrogen Bond<br>Conventional Hydrogen Bond<br>Conventional Hydrogen Bond<br>Conventional Hydrogen Bond<br>Conventional Hydrogen Bond<br>Carbon Hydrogen Bond<br>Carbon Hydrogen Bond<br>Carbon Hydrogen Bond
6.	5-Methoxydimet	SER342<br>GLU376	2.9453<br>3.51871	Hydrogen Bond<br>Hydrogen Bond	Conventional Hydrogen Bond<br>Carbon Hydrogen Bond

	hyltryptamine	THR339	3.46972	Hydrogen Bond	Carbon Hydrogen Bond
	(1832)	GLU293	3.36325	Hydrogen Bond	Carbon Hydrogen Bond
		CYS309	5.64392	Other	Pi-Sulfur
		ALA286	3.79817	Hydrophobic	Alkyl
		VAL312	4.94612	Hydrophobic	Pi-Alkyl
7.	N(alpha)-	CYS287	2.97044	Hydrogen Bond	Conventional Hydrogen Bond
	gamma-	SER342	3.05551	Hydrogen Bond	Conventional Hydrogen Bond
	Lglutamylhista	LYS311	2.78715	Hydrogen Bond	Conventional Hydrogen Bond
	mine (440238)	SER340	2.74771	Hydrogen Bond	Conventional Hydrogen Bond
		CYS287	2.79327	Hydrogen Bond	Conventional Hydrogen Bond
		CYS287	2.61371	Hydrogen Bond	Conventional Hydrogen Bond
		CYS287	2.59651	Hydrogen Bond	Conventional Hydrogen Bond
		SER291	2.27626	Hydrogen Bond	Conventional Hydrogen Bond
8.	Pipercitine	ALA286	5.28595	Hydrophobic	Alkyl
	(12575258)	LYS375	4.58674	Hydrophobic	Alkyl
		LYS375	5.08566	Hydrophobic	Alkyl
		ILE401	4.782	Hydrophobic	Alkyl
		ILE401	4.31482	Hydrophobic	Alkyl
		TYR292	4.4866	Hydrophobic	Pi-Alkyl
9.	Asparaginyl-	ASN210	2.89415	Hydrogen Bond	Conventional Hydrogen Bond
	Cysteine	SER196	3.17185	Hydrogen Bond	Conventional Hydrogen Bond
	(18218178)	SER196	2.78694	Hydrogen Bond	Conventional Hydrogen Bond
		SER196	3.13638	Hydrogen Bond	Conventional Hydrogen Bond
		SER196	2.81636	Hydrogen Bond	Conventional Hydrogen Bond
		CYS207	2.55349	Hydrogen Bond	Conventional Hydrogen Bond
		CYS207	2.31294	Hydrogen Bond	Conventional Hydrogen Bond
		CYS208	2.42932	Hydrogen Bond	Conventional Hydrogen Bond
		PRO219	3.09493	Hydrogen Bond	Conventional Hydrogen Bond
		PRO219	2.85783	Hydrogen Bond	Conventional Hydrogen Bond
		CYS208	2.14183	Hydrogen Bond	Conventional Hydrogen Bond
10.	3beta,6betaDih	SER340	2.97827	Hydrogen Bond	Conventional Hydrogen Bond
	ydroxynortrop	SER340	2.09729	Hydrogen Bond	Conventional Hydrogen Bond
	ane (22297531)	TYR292	2.72688	Hydrogen Bond	Conventional Hydrogen Bond
11.	Macamide B	HIS209	5.06952	Hydrophobic	Pi-Pi T-shaped
	(11198769)	CYS195	5.4758	Hydrophobic	Alkyl
		CYS207	4.84686	Hydrophobic	Alkyl
		PRO219	5.4089	Hydrophobic	Alkyl
12.	N-(14-	ARG220	2.85439	Hydrogen Bond	Conventional Hydrogen Bond
	Methylhexadec	SER196	3.67684	Hydrogen Bond	Carbon Hydrogen Bond
	anoyl)pyrrolidi	ASN210	3.34235	Hydrogen Bond	Carbon Hydrogen Bond
	ne (6430518)	PRO219	4.23093	Hydrophobic	Alkyl
		HIS209	4.48723	Hydrophobic	Pi-Alkyl
13.	(+)-alpha-	TYR292	4.77339	Hydrophobic	Pi-Alkyl
	Pinene (82227)				
14.	Medicanine	CYS287	3.05891	Hydrogen Bond	Conventional Hydrogen Bond
	(101409750)	SER342	2.91264	Hydrogen Bond	Conventional Hydrogen Bond
		GLU293	2.64221	Hydrogen Bond	Conventional Hydrogen Bond
		GLU293	2.49052	Hydrogen Bond	Conventional Hydrogen Bond
15.	Benzyl	GLU293	3.82693	Electrostatic	Attractive Charge
	isothiocyanate	GLU376	3.84338	Electrostatic	Attractive Charge
	(2346)	SER342	3.73386	Hydrogen Bond	Conventional Hydrogen Bond

		TYR292	5.3346	Hydrophobic	Pi-Pi T-shaped
**Binding interaction of phytochemicals with HER3 (ERBB3)**					
1.	p-Coumaroylagmatine (440362)	THR768	3.05025	Hydrogen Bond	Conventional Hydrogen Bond
		ASN820	2.29696	Hydrogen Bond	Conventional Hydrogen Bond
		ASN820	2.36587	Hydrogen Bond	Conventional Hydrogen Bond
		PHE834	5.22797	Hydrophobic	Pi-Pi T-shaped
		VAL704	4.12776	Hydrophobic	Alkyl
		CYS721	4.77485	Hydrophobic	Alkyl
		LEU822	5.05738	Hydrophobic	Alkyl
		ALA832	4.98876	Hydrophobic	Pi-Alkyl
2.	Benzoxazinone glucoside (77195081)	ASP833	2.38489	Hydrogen Bond	Conventional Hydrogen Bond
		GLY697	3.34293	Hydrogen Bond	Carbon Hydrogen Bond
		LEU771	3.57332	Hydrogen Bond	Carbon Hydrogen Bond
		LEU696	3.71065	Hydrophobic	Pi-Sigma
		CYS721	4.38823	Hydrophobic	Alkyl
		LEU771	4.63085	Hydrophobic	Alkyl
		LEU822	4.46392	Hydrophobic	Alkyl
		TYR770	5.31927	Hydrophobic	Pi-Alkyl
		VAL704	5.27454	Hydrophobic	Pi-Alkyl
3.	Macamide B (11198769)	ASP833	2.54012	Hydrogen Bond	Conventional Hydrogen Bond
		THR768	3.58564	Hydrophobic	Pi-Sigma
		PHE834	5.19985	Hydrophobic	Pi-Pi T-shaped
		LEU696	4.78244	Hydrophobic	Alkyl
		VAL704	5.02317	Hydrophobic	Alkyl
		VAL704	4.96138	Hydrophobic	Alkyl
		VAL704	4.36364	Hydrophobic	Alkyl
		CYS721	5.28382	Hydrophobic	Alkyl
		LEU822	4.94335	Hydrophobic	Alkyl
		ALA832	4.75359	Hydrophobic	Alkyl
		LEU755	4.96316	Hydrophobic	Pi-Alkyl
		LEU766	5.41208	Hydrophobic	Pi-Alkyl
4.	Leucyl-Histidine (6992828)	CYS721	3.47246	Hydrogen Bond	Conventional Hydrogen Bond
		THR768	2.70024	Hydrogen Bond	Conventional Hydrogen Bond
		ASP833	3.13478	Hydrogen Bond	Conventional Hydrogen Bond
		THR768	2.8824	Hydrogen Bond	Conventional Hydrogen Bond
		THR768	2.39166	Hydrogen Bond	Conventional Hydrogen Bond
		ASP833	3.29422	Electrostatic	Pi-Anion
		VAL836	5.17945	Hydrophobic	Alkyl
		LEU766	5.03762	Hydrophobic	Alkyl
		PHE834	5.28245	Hydrophobic	Pi-Alkyl
		VAL704	5.35615	Hydrophobic	Pi-Alkyl
		LEU822	5.20119	Hydrophobic	Pi-Alkyl
		ALA832	4.99186	Hydrophobic	Pi-Alkyl
5.	Pipercitine (12575258)	ASP833	3.20782	Hydrogen Bond	Conventional Hydrogen Bond
		VAL753	3.53372	Hydrogen Bond	Carbon Hydrogen Bond
		VAL704	4.7217	Hydrophobic	Alkyl
		VAL704	5.41236	Hydrophobic	Alkyl
		VAL704	3.69745	Hydrophobic	Alkyl
		LEU755	4.62408	Hydrophobic	Alkyl
		LEU766	5.00369	Hydrophobic	Alkyl
		LEU822	4.9626	Hydrophobic	Alkyl

		ALA832<br>LEU696<br>LEU696<br>LEU822<br>PHE834	4.6725<br>4.97501<br>5.23461<br>4.85043<br>5.01004	Hydrophobic<br>Hydrophobic<br>Hydrophobic<br>Hydrophobic<br>Hydrophobic	Alkyl<br>Alkyl<br>Alkyl<br>Alkyl<br>Pi-Alkyl
6.	N(alpha)-gamma-Lglutamylhistamine (440238)	THR768<br>ASP833<br>ASP833<br>THR768<br>CYS721<br>PHE834<br>LEU822<br>LEU771	3.14519<br>3.01629<br>2.6881<br>3.61922<br>3.63653<br>2.40252<br>3.45832<br>4.93538	Hydrogen Bond<br>Hydrogen Bond<br>Hydrogen Bond<br>Hydrogen Bond<br>Hydrogen Bond; Other<br>Hydrogen Bond<br>Hydrophobic<br>Hydrophobic	Conventional Hydrogen Bond<br>Conventional Hydrogen Bond<br>Conventional Hydrogen Bond<br>Carbon Hydrogen Bond<br>Pi-Donor Hydrogen Bond; Pi-Sulfur<br>Pi-Donor Hydrogen Bond<br>Pi-Sigma<br>Pi-Alkyl
7.	N-Acetyl-leucyl-leucin (443129)	LYS723<br>THR768<br>ASN820<br>ASP833<br>CYS721<br>LEU696<br>LEU822<br>ARG819	2.93327<br>3.13107<br>2.26168<br>2.23587<br>4.55856<br>5.43668<br>4.64055<br>4.23726	Hydrogen Bond<br>Hydrogen Bond<br>Hydrogen Bond<br>Hydrogen Bond<br>Hydrophobic<br>Hydrophobic<br>Hydrophobic<br>Hydrophobic	Conventional Hydrogen Bond<br>Conventional Hydrogen Bond<br>Conventional Hydrogen Bond<br>Conventional Hydrogen Bond<br>Alkyl<br>Alkyl<br>Alkyl<br>Alkyl
8.	N-(14-Methylhexadecanoyl)pyrrolidine (6430518)	VAL753<br>LEU696<br>VAL704<br>VAL704<br>VAL704<br>CYS721<br>CYS721<br>VAL753<br>LEU755<br>LEU766<br>LEU822<br>ALA832<br>LEU696<br>LEU822<br>PHE834	3.41459<br>5.46798<br>4.60182<br>4.01671<br>5.29809<br>5.14845<br>4.82221<br>5.1298<br>4.80579<br>5.32262<br>4.89285<br>4.65229<br>5.05027<br>5.09883<br>4.61565	Hydrogen Bond<br>Hydrophobic<br>Hydrophobic<br>Hydrophobic<br>Hydrophobic<br>Hydrophobic<br>Hydrophobic<br>Hydrophobic<br>Hydrophobic<br>Hydrophobic<br>Hydrophobic<br>Hydrophobic<br>Hydrophobic<br>Hydrophobic<br>Hydrophobic	Carbon Hydrogen Bond<br>Alkyl<br>Alkyl<br>Alkyl<br>Alkyl<br>Alkyl<br>Alkyl<br>Alkyl<br>Alkyl<br>Alkyl<br>Alkyl<br>Alkyl<br>Alkyl<br>Alkyl<br>Pi-Alkyl
9.	5-Methoxydimethyltryptamine (1832)	THR768<br>LEU771<br>ARG819<br>CYS721<br>CYS721<br>LEU822<br>ALA832<br>VAL753<br>LEU822<br>LEU822<br>VAL704<br>LEU771	3.11601<br>2.12875<br>3.62724<br>4.1503<br>3.5873<br>3.46939<br>4.01883<br>4.35998<br>5.49536<br>4.53037<br>5.4833<br>5.43809	Hydrogen Bond<br>Hydrogen Bond<br>Hydrogen Bond<br>Hydrogen Bond<br>Hydrogen Bond; Other<br>Hydrophobic<br>Hydrophobic<br>Hydrophobic<br>Hydrophobic<br>Hydrophobic<br>Hydrophobic<br>Hydrophobic	Conventional Hydrogen Bond<br>Conventional Hydrogen Bond<br>Carbon Hydrogen Bond<br>Pi-Donor Hydrogen Bond<br>Pi-Donor Hydrogen Bond; Pi-Sulfur<br>Pi-Sigma<br>Alkyl<br>Alkyl<br>Alkyl<br>Pi-Alkyl<br>Pi-Alkyl<br>Pi-Alkyl
10.	Europine	LYS723	3.13219		Conventional Hydrogen Bond

	(5462451)	SER698	2.86885	Hydrogen Bond Unfavorable	Unfavorable Acceptor-Acceptor
11.	(+)-alpha-Pinene (82227)	ALA894<br>LEU896<br>LEU896<br>ILE967<br>LEU896<br>ARG961	4.6859<br>4.90162<br>4.76128<br>5.16774<br>4.80296<br>4.01651	Hydrophobic<br>Hydrophobic<br>Hydrophobic<br>Hydrophobic<br>Hydrophobic<br>Hydrophobic	Alkyl<br>Alkyl<br>Alkyl<br>Alkyl<br>Alkyl<br>Alkyl
12.	Benzyl isothiocyanate (2346)	ASP833<br>PHE834<br>LEU755<br>LEU766	4.40822<br>5.02096<br>4.80553<br>4.85321	Electrostatic<br>Hydrophobic<br>Hydrophobic<br>Hydrophobic	Attractive Charge<br>Pi-Pi T-shaped<br>Pi-Alkyl<br>Pi-Alkyl
13.	Asparaginyl-Cysteine (18218178)	ARG814<br>ASP843<br>ALA837<br>LEU840<br>TYR869	2.971<br>2.25511<br>2.82864<br>2.34943<br>2.11942	Hydrogen Bond<br>Hydrogen Bond<br>Hydrogen Bond<br>Hydrogen Bond<br>Hydrogen Bond	Conventional Hydrogen Bond<br>Conventional Hydrogen Bond<br>Conventional Hydrogen Bond<br>Conventional Hydrogen Bond<br>Conventional Hydrogen Bond
14.	3beta,6betaDihydroxynortropane (22297531)	ARG814<br>ALA837<br>LEU840<br>ASP843<br>ALA837	3.11853<br>1.90057<br>2.72177<br>2.65221<br>3.18134	Hydrogen Bond<br>Hydrogen Bond<br>Hydrogen Bond<br>Hydrogen Bond<br>Hydrogen Bond	Conventional Hydrogen Bond<br>Conventional Hydrogen Bond<br>Conventional Hydrogen Bond<br>Conventional Hydrogen Bond<br>Carbon Hydrogen Bond
15.	Medicanine (101409750)	THR768	3.11121	Hydrogen Bond	Conventional Hydrogen Bond
**Binding interaction of phytochemicals with Membrane progesterone receptor alpha (mPRα)**					
1.	p-Coumaroylagmatine (440362)	SER121<br>HIS279<br>SER210<br>PHE248<br>VAL146<br>PHE255<br>PHE278	2.21562<br>2.9498<br>3.62262<br>5.26536<br>4.53617<br>4.74039<br>5.10523	Hydrogen Bond<br>Hydrogen Bond<br>Hydrogen Bond<br>Hydrophobic<br>Hydrophobic<br>Hydrophobic<br>Hydrophobic	Conventional Hydrogen Bond<br>Conventional Hydrogen Bond<br>Carbon Hydrogen Bond<br>Pi-Pi T-shaped<br>Alkyl<br>Pi-Alkyl<br>Pi-Alkyl
2.	Macamide B (11198769)	SER153<br>CYS285<br>TYR214<br>PHE248<br>VAL146<br>LEU282<br>TYR143<br>PHE248<br>PHE255<br>PHE255<br>PHE255<br>PHE278<br>PHE278	3.43205<br>5.53016<br>4.83514<br>5.03484<br>4.49791<br>5.08767<br>4.86433<br>4.74496<br>4.22606<br>4.85447<br>5.4992<br>5.47833<br>4.77672	Hydrogen Bond<br>Other<br>Hydrophobic<br>Hydrophobic<br>Hydrophobic<br>Hydrophobic<br>Hydrophobic<br>Hydrophobic<br>Hydrophobic<br>Hydrophobic<br>Hydrophobic<br>Hydrophobic<br>Hydrophobic	Pi-Donor Hydrogen Bond<br>Pi-Sulfur<br>Pi-Pi T-shaped<br>Pi-Pi T-shaped<br>Alkyl<br>Alkyl<br>Pi-Alkyl<br>Pi-Alkyl<br>Pi-Alkyl<br>Pi-Alkyl<br>Pi-Alkyl<br>Pi-Alkyl<br>Pi-Alkyl
3.	Benzoxazinone glucoside (77195081)	TYR143<br>TYR143<br>GLN206<br>GLN206	2.9027<br>2.89351<br>3.3031<br>3.19444	Hydrogen Bond<br>Hydrogen Bond<br>Hydrogen Bond<br>Hydrogen Bond	Conventional Hydrogen Bond<br>Conventional Hydrogen Bond<br>Conventional Hydrogen Bond<br>Conventional Hydrogen Bond

		ASP142<br>HIS275<br>SER189<br>PHE255<br>VAL146<br>PHE278<br>VAL146	2.42193<br>3.592<br>3.36381<br>4.8921<br>4.50476<br>5.02757<br>5.08111	Hydrogen Bond<br>Hydrogen Bond<br>Hydrogen Bond<br>Hydrophobic<br>Hydrophobic<br>Hydrophobic<br>Hydrophobic	Conventional Hydrogen Bond<br>Carbon Hydrogen Bond<br>Carbon Hydrogen Bond<br>Pi-Pi T-shaped<br>Alkyl<br>Pi-Alkyl<br>Pi-Alkyl
4.	N-Acetyl-leucyl-leucin (443129)	SER189<br>SER210<br>ALA252<br>TYR143<br>PHE255<br>PHE255<br>PHE278	2.9489<br>2.88657<br>4.8256<br>5.03055<br>4.29684<br>4.68232<br>5.24352	Hydrogen Bond<br>Hydrogen Bond<br>Hydrophobic<br>Hydrophobic<br>Hydrophobic<br>Hydrophobic<br>Hydrophobic	Conventional Hydrogen Bond<br>Conventional Hydrogen Bond<br>Alkyl<br>Pi-Alkyl<br>Pi-Alkyl<br>Pi-Alkyl<br>Pi-Alkyl
5.	N-(14- Methyl hexadecanoyl) pyrrolidine (6430518)	VAL146<br>VAL146<br>VAL146<br>TYR143<br>TYR214<br>PHE248<br>PHE248<br>PHE255<br>PHE255<br>PHE255<br>PHE278<br>PHE278	4.62609<br>5.35829<br>4.78395<br>5.23764<br>5.00285<br>5.00904<br>5.04124<br>4.14243<br>5.15456<br>5.21795<br>5.09248<br>4.90441	Hydrophobic<br>Hydrophobic<br>Hydrophobic<br>Hydrophobic<br>Hydrophobic<br>Hydrophobic<br>Hydrophobic<br>Hydrophobic<br>Hydrophobic<br>Hydrophobic<br>Hydrophobic<br>Hydrophobic	Alkyl<br>Alkyl<br>Alkyl<br>Pi-Alkyl<br>Pi-Alkyl<br>Pi-Alkyl<br>Pi-Alkyl<br>Pi-Alkyl<br>Pi-Alkyl<br>Pi-Alkyl<br>Pi-Alkyl<br>Pi-Alkyl
6.	5-Methoxydimethyltryptamine (1832)	PHE248<br>PHE248<br>TYR149	4.94413<br>5.22147<br>4.75763	Hydrophobic<br>Hydrophobic<br>Hydrophobic	Pi-Pi T-shaped<br>Pi-Pi T-shaped<br>Pi-Alkyl
7.	N(alpha)-gamma-Lglutamylhistamine (440238)	GLN289<br>HIS157<br>HIS157<br>VAL146<br>LEU282	2.39806<br>3.6454<br>3.30818<br>3.75193<br>5.22099	Hydrogen Bond<br>Hydrogen Bond<br>Hydrogen Bond<br>Hydrophobic<br>Hydrophobic	Conventional Hydrogen Bond<br>Carbon Hydrogen Bond<br>Carbon Hydrogen Bond<br>Pi-Sigma<br>Pi-Alkyl
8.	Leucyl-Histidine (6992828)	ASN192<br>ARG203<br>GLN206<br>GLN206<br>GLY199<br>ASN192<br>PHE255<br>PHE255<br>PHE258<br>PHE258	3.23827<br>2.94052<br>2.73365<br>2.49374<br>2.02343<br>3.8422<br>3.89379<br>4.64591<br>5.11343<br>4.64637	Hydrogen Bond<br>Hydrogen Bond<br>Hydrogen Bond<br>Hydrogen Bond<br>Hydrogen Bond<br>Hydrogen Bond<br>Hydrophobic<br>Hydrophobic<br>Hydrophobic<br>Hydrophobic	Conventional Hydrogen Bond<br>Conventional Hydrogen Bond<br>Conventional Hydrogen Bond<br>Conventional Hydrogen Bond<br>Conventional Hydrogen Bond<br>Pi-Donor Hydrogen Bond<br>Pi-Sigma<br>Pi-Alkyl<br>Pi-Alkyl<br>Pi-Alkyl
9.	(+)-alpha-Pinene (82227)	PHE248<br>PHE255<br>ALA252<br>PHE248<br>PHE255<br>PHE255	3.94134<br>3.61584<br>4.37019<br>5.27436<br>5.02733<br>5.09001	Hydrophobic<br>Hydrophobic<br>Hydrophobic<br>Hydrophobic<br>Hydrophobic<br>Hydrophobic	Pi-Sigma<br>Pi-Sigma<br>Alkyl<br>Pi-Alkyl<br>Pi-Alkyl<br>Pi-Alkyl

		PHE278<br>PHE278	5.29087<br>4.75919	Hydrophobic<br>Hydrophobic	Pi-Alkyl<br>Pi-Alkyl
10.	Medicanine (101409750)	TYR149<br>CYS285<br>SER210<br>GLN244<br>ASP217	3.12721<br>3.5001<br>2.33249<br>3.03255<br>3.63311	Hydrogen Bond<br>Hydrogen Bond<br>Hydrogen Bond<br>Hydrogen Bond<br>Hydrogen Bond	Conventional Hydrogen Bond<br>Conventional Hydrogen Bond<br>Conventional Hydrogen Bond<br>Conventional Hydrogen Bond<br>Carbon Hydrogen Bond
11.	Europine (5462451)	HIS275<br>PHE255<br>GLU261<br>HIS275	3.01183<br>3.7378<br>3.7882<br>3.8851	Hydrogen Bond<br>Hydrogen Bond<br>Hydrogen Bond<br>Hydrophobic	Conventional Hydrogen Bond<br>Carbon Hydrogen Bond<br>Carbon Hydrogen Bond<br>Pi-Sigma
12.	Pipercitine (12575258)	LYS44<br>LYS193<br>LYS44<br>PRO45<br>ILE47<br>PHE139<br>PHE139<br>PHE255<br>HIS275<br>HIS275	4.3037<br>5.19353<br>3.78145<br>4.3971<br>5.14781<br>5.33732<br>4.87085<br>5.35812<br>4.54026<br>4.99034	Hydrophobic<br>Hydrophobic<br>Hydrophobic<br>Hydrophobic<br>Hydrophobic<br>Hydrophobic<br>Hydrophobic<br>Hydrophobic<br>Hydrophobic<br>Hydrophobic	Alkyl<br>Alkyl<br>Alkyl<br>Alkyl<br>Alkyl<br>Pi-Alkyl<br>Pi-Alkyl<br>Pi-Alkyl<br>Pi-Alkyl<br>Pi-Alkyl
13.	3beta,6betaDihydroxynortropane (22297531)	HIS103<br>GLU305<br>HIS308<br>GLU305	3.08318<br>2.90576<br>2.1799<br>2.54785	Hydrogen Bond<br>Hydrogen Bond<br>Hydrogen Bond<br>Hydrogen Bond	Conventional Hydrogen Bond<br>Conventional Hydrogen Bond<br>Conventional Hydrogen Bond<br>Conventional Hydrogen Bond
14.	Asparaginyl-Cysteine (18218178)	SER189<br>SER210<br>SER210<br>GLU207<br>GLU207<br>TYR143	3.06819<br>2.87164<br>2.81227<br>2.38284<br>2.3865<br>4.97875	Hydrogen Bond<br>Hydrogen Bond<br>Hydrogen Bond<br>Hydrogen Bond<br>Hydrogen Bond<br>Other	Conventional Hydrogen Bond<br>Conventional Hydrogen Bond<br>Conventional Hydrogen Bond<br>Conventional Hydrogen Bond<br>Conventional Hydrogen Bond<br>Pi-Sulfur
15.	Benzyl isothiocyanate (2346)	PRO176<br>TRP183<br>TRP183<br>ALA179,<br>PHE180<br>ALA179<br>LEU321	3.43241<br>5.32935<br>5.44118<br>4.16116<br>4.8202<br>4.64882	Hydrogen Bond<br>Hydrophobic<br>Hydrophobic<br>Hydrophobic<br>Hydrophobic<br>Hydrophobic	Carbon Hydrogen Bond<br>Pi-Pi Stacked<br>Pi-Pi Stacked<br>Amide-Pi Stacked<br>Pi-Alkyl<br>Pi-Alkyl
**Binding interaction of phytochemicals with G-Protein Coupled Estrogen Receptor (GPER / GPR30)**					
1.	Benzoxazinone glucoside (77195081)	TYR175<br>ARG191<br>ARG191<br>ASN171<br>TYR173<br>TYR173<br>HIS232	2.94358<br>3.03411<br>3.11128<br>2.65002<br>2.06413<br>4.237<br>5.04661	Hydrogen Bond<br>Hydrogen Bond<br>Hydrogen Bond<br>Hydrogen Bond<br>Hydrogen Bond<br>Hydrophobic<br>Hydrophobic	Conventional Hydrogen Bond<br>Conventional Hydrogen Bond<br>Conventional Hydrogen Bond<br>Conventional Hydrogen Bond<br>Conventional Hydrogen Bond<br>Pi-Pi Stacked<br>Pi-Pi T-shaped

2.	p-Coumaroylagmatine (440362)	LEU237<br>LEU233<br>LEU233<br>THR63<br>ALA61<br>TRP47<br>TYR59	3.08722<br>2.63136<br>2.5487<br>2.11562<br>3.66419<br>4.76563<br>4.6864	Hydrogen Bond<br>Hydrogen Bond<br>Hydrogen Bond<br>Hydrogen Bond<br>Hydrophobic<br>Hydrophobic<br>Hydrophobic	Conventional Hydrogen Bond<br>Conventional Hydrogen Bond<br>Conventional Hydrogen Bond<br>Conventional Hydrogen Bond<br>Pi-Sigma<br>Pi-Alkyl<br>Pi-Alkyl
3.	Pipercitine (12575258)	ALA188<br>SER187<br>PHE153<br>ALA184<br>ALA188<br>ALA188<br>VAL191<br>LEU217<br>ILE229<br>ILE229<br>LEU221<br>PHE146<br>PHE146<br>TRP150<br>TRP150<br>PHE153	3.17025<br>3.5567<br>3.7871<br>3.99907<br>4.99441<br>4.79651<br>5.06516<br>5.04867<br>5.4496<br>4.69182<br>4.76145<br>4.27664<br>4.66954<br>5.27329<br>5.33071<br>4.36505	Hydrogen Bond<br>Hydrogen Bond<br>Hydrophobic<br>Hydrophobic<br>Hydrophobic<br>Hydrophobic<br>Hydrophobic<br>Hydrophobic<br>Hydrophobic<br>Hydrophobic<br>Hydrophobic<br>Hydrophobic<br>Hydrophobic<br>Hydrophobic<br>Hydrophobic<br>Hydrophobic	Conventional Hydrogen Bond<br>Carbon Hydrogen Bond<br>Pi-Sigma<br>Alkyl<br>Alkyl<br>Alkyl<br>Alkyl<br>Alkyl<br>Alkyl<br>Alkyl<br>Alkyl<br>Pi-Alkyl<br>Pi-Alkyl<br>Pi-Alkyl<br>Pi-Alkyl<br>Pi-Alkyl
4.	Macamide B (11198769)	SER177<br>PHE168<br>HIS173<br>ILE181<br>ALA184<br>ALA184<br>ILE229<br>PHE146<br>PHE146<br>TRP150<br>TRP150	2.38459<br>5.22709<br>5.29132<br>4.64075<br>5.27355<br>3.99945<br>4.72044<br>4.35029<br>4.51377<br>5.29743<br>4.96599	Hydrogen Bond<br>Hydrophobic<br>Hydrophobic<br>Hydrophobic<br>Hydrophobic<br>Hydrophobic<br>Hydrophobic<br>Hydrophobic<br>Hydrophobic<br>Hydrophobic<br>Hydrophobic	Conventional Hydrogen Bond<br>Pi-Pi T-shaped<br>Pi-Pi T-shaped<br>Alkyl<br>Alkyl<br>Alkyl<br>Alkyl<br>Pi-Alkyl<br>Pi-Alkyl<br>Pi-Alkyl<br>Pi-Alkyl
5.	Europine (5462451)	ASN118<br>HIS307<br>ASN118<br>HIS120<br>PRO303	3.03936<br>3.14722<br>2.58371<br>3.62749<br>3.47026	Hydrogen Bond<br>Hydrogen Bond<br>Hydrogen Bond<br>Hydrogen Bond<br>Hydrogen Bond	Conventional Hydrogen Bond<br>Conventional Hydrogen Bond<br>Conventional Hydrogen Bond<br>Carbon Hydrogen Bond<br>Carbon Hydrogen Bond
6.	N-(14-Methylhexadecanoyl)pyrrolidine (6430518)	TRP150<br>LEU180<br>ALA184<br>ILE229<br>ILE229<br>ILE181<br>PHE146<br>TRP150<br>TRP150<br>PHE153	3.74675<br>5.40498<br>4.10854<br>5.33549<br>5.27464<br>5.17705<br>4.15386<br>4.88099<br>4.47218<br>4.84281	Hydrophobic<br>Hydrophobic<br>Hydrophobic<br>Hydrophobic<br>Hydrophobic<br>Hydrophobic<br>Hydrophobic<br>Hydrophobic<br>Hydrophobic<br>Hydrophobic	Pi-Sigma<br>Alkyl<br>Alkyl<br>Alkyl<br>Alkyl<br>Alkyl<br>Pi-Alkyl<br>Pi-Alkyl<br>Pi-Alkyl<br>Pi-Alkyl
7.	Leucyl-Histidine	THR330<br>THR330	3.07028<br>3.20943	Hydrogen Bond<br>Hydrogen Bond	Conventional Hydrogen Bond<br>Conventional Hydrogen Bond

	(6992828)	PHE331<br>SER325<br>ASP95<br>PHE331<br>PRO94<br>LYS334	2.91582<br>2.27032<br>3.34553<br>5.12297<br>4.46596<br>5.38041	Hydrogen Bond<br>Hydrogen Bond<br>Electrostatic<br>Hydrophobic<br>Hydrophobic<br>Hydrophobic	Conventional Hydrogen Bond<br>Conventional Hydrogen Bond<br>Pi-Anion<br>Pi-Pi T-shaped<br>Alkyl<br>Pi-Alkyl
8.	N-Acetyl-leucyl-leucin (443129)	TRP150<br>TRP150<br>PHE153<br>ALA184<br>ILE229<br>LEU180<br>TRP150<br>PHE153	2.72473<br>2.58866<br>3.99183<br>4.92721<br>5.27616<br>4.21281<br>5.25716<br>5.11746	Hydrogen Bond<br>Hydrogen Bond<br>Hydrophobic<br>Hydrophobic<br>Hydrophobic<br>Hydrophobic<br>Hydrophobic<br>Hydrophobic	Pi-Donor Hydrogen Bond<br>Pi-Donor Hydrogen Bond<br>Pi-Sigma<br>Alkyl<br>Alkyl<br>Alkyl<br>Pi-Alkyl<br>Pi-Alkyl
9.	5-Methoxydimethyltryptamine (1832)	THR63<br>THR63<br>LEU237<br>TYR60<br>ASP62<br>ALA61<br>ALA61	3.27426<br>2.75589<br>2.11506<br>3.55712<br>3.50011<br>3.91462<br>3.70711	Hydrogen Bond<br>Hydrogen Bond<br>Hydrogen Bond<br>Hydrogen Bond<br>Hydrogen Bond<br>Hydrophobic<br>Hydrophobic	Conventional Hydrogen Bond<br>Conventional Hydrogen Bond<br>Conventional Hydrogen Bond<br>Carbon Hydrogen Bond<br>Carbon Hydrogen Bond<br>Pi-Sigma<br>Pi-Sigma
10.	N(alpha)-gamma-Lglutamylhistamine (440238)	ARG155<br>THR330<br>GLY328<br>MET151<br>MET151<br>TYR97<br>PRO94<br>ARG155	3.04295<br>2.40165<br>3.46328<br>3.62185<br>5.75542<br>5.70992<br>4.31816<br>3.93223	Hydrogen Bond<br>Hydrogen Bond<br>Hydrogen Bond<br>Hydrogen Bond<br>Other<br>Hydrophobic<br>Hydrophobic<br>Hydrophobic	Conventional Hydrogen Bond<br>Conventional Hydrogen Bond<br>Carbon Hydrogen Bond<br>Carbon Hydrogen Bond<br>Pi-Sulfur<br>Pi-Pi Stacked<br>Pi-Alkyl<br>Pi-Alkyl
11.	(+)-alpha-Pinene (82227)	PRO192<br>VAL196<br>VAL214	5.38481<br>3.90151<br>4.1367	Hydrophobic<br>Hydrophobic<br>Hydrophobic	Alkyl<br>Alkyl<br>Alkyl
12.	Asparaginyl-Cysteine (18218178)	TRP111<br>GLN186<br>ASP109<br>TRP111<br>PRO185<br>LEU187<br>PHE110	3.16599<br>2.9637<br>2.44268<br>2.6926<br>2.70489<br>2.01031<br>4.99392	Hydrogen Bond<br>Hydrogen Bond<br>Hydrogen Bond<br>Hydrogen Bond<br>Hydrogen Bond<br>Hydrogen Bond<br>Other	Conventional Hydrogen Bond<br>Conventional Hydrogen Bond<br>Conventional Hydrogen Bond<br>Conventional Hydrogen Bond<br>Conventional Hydrogen Bond<br>Conventional Hydrogen Bond<br>Pi-Sulfur
13.	Benzyl Isothiocyanate (2346)	PHE146<br>PHE146<br>ALA184	3.46249<br>3.71312<br>5.1243	Hydrogen Bond<br>Hydrophobic<br>Hydrophobic	Carbon Hydrogen Bond<br>Pi-Pi Stacked<br>Pi-Alkyl
14.	3beta,6betaDihydroxynortropane (22297531)	ASP210	2.98251	Hydrogen Bond	Conventional Hydrogen Bond
15.	Medicanine (101409750)	ASP210<br>GLN215<br>ALA188	3.04716<br>3.28473<br>3.68951	Hydrogen Bond<br>Hydrogen Bond<br>Hydrogen Bond	Conventional Hydrogen Bond<br>Conventional Hydrogen Bond<br>Carbon Hydrogen Bond

Table S5. Binding interaction studies of identified phytomolecules from *S. persica* root extract at negative-ESI mode against therapeutic membrane receptors of breast cancer, *viz.* Estrogen Receptor α (ERα / ESR1; PDB ID: 1R5K), Progesterone receptor (PR; PDB ID: 4OAR), Insulin-Like Growth Factor-1 Receptor (IGF-1R; PDB ID: 1IGR), Epidermal Growth Factor Receptor (EGFR / ERBB1; PDB ID: 1IVO), Human Epidermal growth factor Receptor 3 (HER3; PDB ID: 3KEX), Membrane progesterone receptor alpha (mPRα; PDB identifier/UniProt entry Q86WK9) and G-Protein Coupled Estrogen Receptor (GPER/ GPR30; PDB ID: 8XOG) through PyRx docking tool

S. No.	Binding interaction of phytochemicals with Estrogen Receptor α (ERα / ESR1)				
	**Phytochemicals**	**Interacting Amino acid residues**	**Bond Distance (Å)**	**Bond Category**	**Bond Types**
1.	Quercetin 3-(2-caffeoylglucuronoside) (131753131)	TRP393	3.04756	Hydrogen Bond	Conventional Hydrogen Bond
		GLN441	2.93847	Hydrogen Bond	Conventional Hydrogen Bond
		HIS398	2.22982	Hydrogen Bond	Conventional Hydrogen Bond
		ASN439	2.84289	Hydrogen Bond	Conventional Hydrogen Bond
		ARG394	2.4589	Hydrogen Bond	Conventional Hydrogen Bond
		ARG394	3.84847	Electrostatic	Pi-Alkyl
		LYS449	4.09664	Electrostatic	Pi-Anion
		GLU323	4.38813	Electrostatic	Pi-Cation
		TRP393	4.85114	Hydrophobic	Pi-Cation
		TRP393	4.92925	Hydrophobic	Pi-Pi T-shaped
		PRO324	5.16262	Hydrophobic	Pi-Pi T-shaped
2.	(-)-Epicatechin 3'-O-glucuronide (76969982)	THR347	3.15927	Hydrogen Bond	Conventional Hydrogen Bond
		LEU525	2.1758	Hydrogen Bond	Conventional Hydrogen Bond
		THR347	3.88038	Hydrophobic	Pi-Sigma
		ALA350	4.7004	Hydrophobic	Pi-Alkyl
		LEU525	4.91751	Hydrophobic	Pi-Alkyl
		LEU346	4.94503	Hydrophobic	Pi-Alkyl
		ALA350	4.84676	Hydrophobic	Pi-Alkyl
3.	Glucotropaeolin (9548605)	THR347	3.04266	Hydrogen Bond	Conventional Hydrogen Bond
		GLU353	2.56582	Hydrogen Bond	Conventional Hydrogen Bond
		THR347	2.9406	Hydrogen Bond	Conventional Hydrogen Bond
		TRP383	5.94972	Other	Pi-Sulfur
		ILE424	5.41019	Hydrophobic	Pi-Alkyl
		LEU525	5.30277	Hydrophobic	Pi-Alkyl
4.	8S-HODE (16061037)	LEU346	4.25244	Hydrophobic	Alkyl
		ALA350	3.96277	Hydrophobic	Alkyl
		LEU387	5.30284	Hydrophobic	Alkyl
		MET388	4.15642	Hydrophobic	Alkyl
		LEU391	5.11667	Hydrophobic	Alkyl
		LEU346	4.95653	Hydrophobic	Alkyl
		LEU349	4.53816	Hydrophobic	Alkyl
		LEU384	4.92098	Hydrophobic	Alkyl
		LEU525	4.39285	Hydrophobic	Alkyl
		PHE404	5.04463	Hydrophobic	Pi-Alkyl
5.	3-Keto stearic acid (5283005)	ARG394	2.80699	Hydrogen Bond	Conventional Hydrogen Bond
		ALA350	4.49823	Hydrophobic	Alkyl
		ALA350	4.29812	Hydrophobic	Alkyl
		LEU384	5.11997	Hydrophobic	Alkyl

		LEU525<br>MET421<br>ILE424<br>LEU525<br>LEU525<br>TRP383	4.73204<br>4.77738<br>4.85971<br>4.78558<br>4.5344<br>5.4698	Hydrophobic<br>Hydrophobic<br>Hydrophobic<br>Hydrophobic<br>Hydrophobic<br>Hydrophobic	Alkyl<br>Alkyl<br>Alkyl<br>Alkyl<br>Alkyl<br>Pi-Alkyl
6.	16-Hydroxy hexadecanoic acid (10466)	THR347<br>LEU346<br>ALA350<br>MET388<br>MET421<br>ILE424<br>LEU387<br>PHE404	3.13641<br>5.43671<br>4.52208<br>5.39219<br>4.92911<br>4.06794<br>4.15865<br>5.298	Hydrogen Bond<br>Hydrophobic<br>Hydrophobic<br>Hydrophobic<br>Hydrophobic<br>Hydrophobic<br>Hydrophobic<br>Hydrophobic	Conventional Hydrogen Bond<br>Alkyl<br>Alkyl<br>Alkyl<br>Alkyl<br>Alkyl<br>Alkyl<br>Pi-Alkyl
7.	Benzyl isothiocyanate (2346)	PHE404<br>PHE425<br>PHE404<br>ALA350<br>LEU387<br>LEU391	5.00106<br>5.5368<br>5.39988<br>5.02303<br>4.17316<br>5.03313	Other<br>Other<br>Hydrophobic<br>Hydrophobic<br>Hydrophobic<br>Hydrophobic	Pi-Sulfur<br>Pi-Sulfur<br>Pi-Pi T-shaped<br>Pi-Alkyl<br>Pi-Alkyl<br>Pi-Alkyl
**Binding interaction of phytochemicals with Progesterone receptor**					
1.	Quercetin 3-(2-caffeoylglucuronoside) (131753131)	ARG766<br>CYS891<br>SER898<br>MET759<br>THR894<br>ASN719<br>ASN719<br>CYS891<br>PHE778<br>CYS891<br>MET759<br>LEU763	3.16289<br>3.76444<br>3.12107<br>2.12132<br>2.59363<br>3.26735<br>3.67672<br>5.35881<br>4.8263<br>5.40758<br>5.40231<br>4.78734	Hydrogen Bond<br>Hydrogen Bond<br>Hydrogen Bond<br>Hydrogen Bond<br>Hydrogen Bond<br>Hydrogen Bond<br>Hydrogen Bond<br>Other<br>Hydrophobic<br>Hydrophobic<br>Hydrophobic<br>Hydrophobic	Conventional Hydrogen Bond<br>Conventional Hydrogen Bond<br>Conventional Hydrogen Bond<br>Conventional Hydrogen Bond<br>Conventional Hydrogen Bond<br>Carbon Hydrogen Bond<br>Pi-Donor Hydrogen Bond<br>Pi-Sulfur<br>Pi-Pi T-shaped<br>Pi-Alkyl<br>Pi-Alkyl<br>Pi-Alkyl
2.	(-)-Epicatechin 3'-O-glucuronide (76969982)	ARG766<br>GLN815<br>GLU695<br>GLN725<br>SER728<br>ARG766<br>VAL698<br>ARG766<br>PRO696	3.19818<br>2.80237<br>2.61284<br>2.1725<br>3.53876<br>4.44345<br>5.03143<br>4.75543<br>4.71127	Hydrogen Bond<br>Hydrogen Bond<br>Hydrogen Bond<br>Hydrogen Bond<br>Hydrogen Bond<br>Electrostatic<br>Hydrophobic<br>Hydrophobic<br>Hydrophobic	Conventional Hydrogen Bond<br>Conventional Hydrogen Bond<br>Conventional Hydrogen Bond<br>Conventional Hydrogen Bond<br>Carbon Hydrogen Bond<br>Pi-Cation<br>Pi-Alkyl<br>Pi-Alkyl<br>Pi-Alkyl
3.	Glucotropaeolin (9548605)	TRP765<br>ARG766<br>ARG766<br>GLN815<br>LYS822<br>TRP765<br>ARG766<br>TRP765<br>VAL698	3.09354<br>2.79948<br>2.9413<br>3.07036<br>2.98499<br>3.50795<br>4.93865<br>5.61066<br>4.65678	Hydrogen Bond<br>Hydrogen Bond<br>Hydrogen Bond<br>Hydrogen Bond<br>Hydrogen Bond<br>Hydrogen Bond<br>Electrostatic<br>Other<br>Hydrophobic	Conventional Hydrogen Bond<br>Conventional Hydrogen Bond<br>Conventional Hydrogen Bond<br>Conventional Hydrogen Bond<br>Conventional Hydrogen Bond<br>Carbon Hydrogen Bond<br>Pi-Cation<br>Pi-Sulfur<br>Pi-Alkyl

		ARG766	4.89363	Hydrophobic	Pi-Alkyl
4.	8S-HODE (16061037)	VAL760<br>MET756<br>LEU718<br>MET756<br>VAL760<br>LEU797<br>MET801<br>LEU887<br>MET759<br>LEU763<br>LEU718<br>LEU721<br>PHE778<br>PHE778<br>PHE778	3.11869<br>2.74539<br>3.79969<br>5.12764<br>5.35465<br>4.62499<br>5.26325<br>3.96025<br>5.19077<br>4.29732<br>5.39423<br>5.0938<br>5.43838<br>5.09508<br>5.22212	Hydrogen Bond<br>Hydrogen Bond<br>Hydrophobic<br>Hydrophobic<br>Hydrophobic<br>Hydrophobic<br>Hydrophobic<br>Hydrophobic<br>Hydrophobic<br>Hydrophobic<br>Hydrophobic<br>Hydrophobic<br>Hydrophobic<br>Hydrophobic<br>Hydrophobic	Conventional Hydrogen Bond<br>Conventional Hydrogen Bond<br>Alkyl<br>Alkyl<br>Alkyl<br>Alkyl<br>Alkyl<br>Alkyl<br>Alkyl<br>Alkyl<br>Alkyl<br>Alkyl<br>Pi-Alkyl<br>Pi-Alkyl<br>Pi-Alkyl
5.	16-Hydroxy hexadecanoic acid (10466)	GLN725<br>MET801<br>LEU887<br>CYS891<br>LEU797<br>MET759	2.78534<br>5.46389<br>5.2515<br>4.61909<br>4.43178<br>5.21219	Hydrogen Bond<br>Hydrophobic<br>Hydrophobic<br>Hydrophobic<br>Hydrophobic<br>Hydrophobic	Conventional Hydrogen Bond<br>Alkyl<br>Alkyl<br>Alkyl<br>Alkyl<br>Alkyl
6.	3-Keto stearic acid (5283005)	THR800<br>GLN803<br>SER796<br>LEU799<br>LEU799<br>TRP802<br>TRP802<br>TRP802	3.28338<br>3.0065<br>2.63745<br>5.03735<br>5.44221<br>4.71508<br>4.67925<br>5.10103	Hydrogen Bond<br>Hydrogen Bond<br>Hydrogen Bond<br>Hydrophobic<br>Hydrophobic<br>Hydrophobic<br>Hydrophobic<br>Hydrophobic	Conventional Hydrogen Bond<br>Conventional Hydrogen Bond<br>Conventional Hydrogen Bond<br>Alkyl<br>Alkyl<br>Pi-Alkyl<br>Pi-Alkyl<br>Pi-Alkyl
7.	Benzyl isothiocyanate (2346)	LEU715<br>TRP755<br>PHE794<br>LEU797	3.88433<br>5.97134<br>4.89463<br>5.072	Hydrophobic<br>Other<br>Hydrophobic<br>Hydrophobic	Pi-Sigma<br>Pi-Sulfur<br>Pi-Pi T-shaped<br>Pi-Alkyl
**Binding interaction of phytochemicals with Insulin-Like Growth Factor-1 Receptor (IGF-1R)**					
1.	(-)-Epicatechin 3'-O-glucuronide (76969982)	ARG104<br>ASN105<br>ARG222<br>ARG104<br>GLU163<br>MET184<br>LEU129	3.27744<br>3.04352<br>3.13124<br>2.3582<br>2.86238<br>5.88595<br>4.46192	Hydrogen Bond<br>Hydrogen Bond<br>Hydrogen Bond<br>Hydrogen Bond<br>Hydrogen Bond<br>Other<br>Hydrophobic	Conventional Hydrogen Bond<br>Conventional Hydrogen Bond<br>Conventional Hydrogen Bond<br>Conventional Hydrogen Bond<br>Conventional Hydrogen Bond<br>Pi-Sulfur<br>Pi-Alkyl
2.	Quercetin 3-(2-caffeoylglucuronoside) (131753131)	ASN105<br>ASN105<br>ARG222<br>ARG222<br>ARG222<br>GLY190<br>LYS191<br>ASP132<br>LEU129	3.03778<br>2.85777<br>3.20918<br>3.19985<br>3.21325<br>2.35063<br>2.57839<br>3.28595<br>5.0096	Hydrogen Bond<br>Hydrogen Bond<br>Hydrogen Bond<br>Hydrogen Bond<br>Hydrogen Bond<br>Hydrogen Bond<br>Hydrogen Bond<br>Hydrogen Bond<br>Hydrophobic	Conventional Hydrogen Bond<br>Conventional Hydrogen Bond<br>Conventional Hydrogen Bond<br>Conventional Hydrogen Bond<br>Conventional Hydrogen Bond<br>Conventional Hydrogen Bond<br>Conventional Hydrogen Bond<br>Carbon Hydrogen Bond<br>Pi-Alkyl

		LEU129	5.00507	Hydrophobic	Pi-Alkyl
		ALA193	4.87928	Hydrophobic	Pi-Alkyl
3.	Glucotropa	GLY207	3.05213	Hydrogen Bond	Conventional Hydrogen Bond
	eolin	ARG222	2.96609	Hydrogen Bond	Conventional Hydrogen Bond
	(9548605)	ARG222	3.12593	Hydrogen Bond	Conventional Hydrogen Bond
		ASP132	2.69948	Hydrogen Bond	Conventional Hydrogen Bond
		ARG104	3.06521	Hydrogen Bond	Conventional Hydrogen Bond
		ARG104	3.58999	Hydrogen Bond;	Pi-Cation; Pi-Donor Hydrogen
		ARG104	4.04744	Electrostatic	Bond
		ARG104	5.33156	Hydrogen Bond	Pi-Donor Hydrogen Bond
		LEU129	4.87023	Hydrophobic	Pi-Alkyl
		CYS185	5.35437	Hydrophobic	Pi-Alkyl
				Hydrophobic	Pi-Alkyl
4.	8S-HODE	ARG77	3.21312	Hydrogen Bond	Conventional Hydrogen Bond
	(16061037)	ARG77	2.88038	Hydrogen Bond	Conventional Hydrogen Bond
		CYS218	2.91435	Hydrogen Bond	Conventional Hydrogen Bond
		CYS221	2.81012	Hydrogen Bond	Conventional Hydrogen Bond
		THR216	2.26737	Hydrogen Bond	Conventional Hydrogen Bond
		CYS221	4.74572	Hydrophobic	Alkyl
		ILE2	4.90731	Hydrophobic	Alkyl
		TYR225	4.57239	Hydrophobic	Pi-Alkyl
		TYR225	5.03809	Hydrophobic	Pi-Alkyl
		TYR225	5.10931	Hydrophobic	Pi-Alkyl
		TYR225	4.02508	Hydrophobic	Pi-Alkyl
		TRP244	4.80278	Hydrophobic	Pi-Alkyl
5.	16-	ARG10	3.16717	Hydrogen Bond	Conventional Hydrogen Bond
	Hydroxy	ARG10	2.99397	Hydrogen Bond	Conventional Hydrogen Bond
	hexadecano	ARG10	3.07377	Hydrogen Bond	Conventional Hydrogen Bond
	ic acid	VAL88	5.47697	Hydrophobic	Alkyl
	(10466)	LEU56	4.976	Hydrophobic	Alkyl
		LEU32	4.25504	Hydrophobic	Alkyl
		LEU56	4.28386	Hydrophobic	Alkyl
		HIS30	5.36898	Hydrophobic	Pi-Alkyl
		PHE58	4.0402	Hydrophobic	Pi-Alkyl
		PHE58	5.31183	Hydrophobic	Pi-Alkyl
		PHE58	4.47232	Hydrophobic	Pi-Alkyl
		PHE82	4.76016	Hydrophobic	Pi-Alkyl
		PHE90	3.84596	Hydrophobic	Pi-Alkyl
		PHE90	4.65484	Hydrophobic	Pi-Alkyl
6.	Benzyl	ASP126	4.19838	Electrostatic	Attractive Charge
	isothiocyan	TRP127	4.75411	Hydrophobic	Pi-Pi T-shaped
	ate (2346)	VAL134	5.36488	Hydrophobic	Pi-Alkyl
		ILE167	5.27335	Hydrophobic	Pi-Alkyl
7.	3-Keto	ASN105	2.94123	Hydrogen Bond	Conventional Hydrogen Bond
	stearic acid	ASP132	2.59276	Hydrogen Bond	Conventional Hydrogen Bond
	(5283005)	ARG104	4.68591	Hydrophobic	Alkyl
		CYS185	4.60195	Hydrophobic	Alkyl
		ALA193	4.67842	Hydrophobic	Alkyl
		ALA193	5.46004	Hydrophobic	Alkyl
		LEU129	5.37487	Hydrophobic	Alkyl
		LEU129	4.44379	Hydrophobic	Alkyl

Binding interaction with Epidermal Growth Factor Receptor (EGFR / ERBB1)					
1.	Quercetin 3-(2-caffeoylglucuronoside) (131753131)	GLN8	3.39236	Hydrogen Bond	Conventional Hydrogen Bond
		ARG285	2.7161	Hydrogen Bond	Conventional Hydrogen Bond
		ARG285	3.1622	Hydrogen Bond	Conventional Hydrogen Bond
		LYS407	3.20194	Hydrogen Bond	Conventional Hydrogen Bond
		TYR275	3.05294	Hydrogen Bond	Conventional Hydrogen Bond
		THR406	3.19902	Hydrogen Bond	Carbon Hydrogen Bond
		LYS407	3.70494	Hydrogen Bond	Carbon Hydrogen Bond
		LEU38	3.87357	Hydrophobic	Pi-Sigma
		LEU38	3.91852	Hydrophobic	Pi-Sigma
		ALA62	5.01832	Hydrophobic	Pi-Alkyl
		ILE318	4.22767	Hydrophobic	Pi-Alkyl
2.	(-)-Epicatechin 3'-O-glucuronide (76969982)	SER340	2.81151	Hydrogen Bond	Conventional Hydrogen Bond
		SER340	3.04758	Hydrogen Bond	Conventional Hydrogen Bond
		SER342	2.99001	Hydrogen Bond	Conventional Hydrogen Bond
		CYS287	2.52981	Hydrogen Bond	Conventional Hydrogen Bond
		CYS287	2.01829	Hydrogen Bond	Conventional Hydrogen Bond
		GLU376	2.44622	Hydrogen Bond	Conventional Hydrogen Bond
		LYS311	2.62241	Hydrogen Bond	Conventional Hydrogen Bond
		SER342	3.70988	Hydrogen Bond	Carbon Hydrogen Bond
		THR339	3.76221	Hydrogen Bond	Carbon Hydrogen Bond
		LYS311	3.99219	Hydrogen Bond;	Pi-Cation; Pi-Donor Hydrogen
		GLU376	3.82732	Electrostatic	Bond
		ARG310	5.12017	Electrostatic	Pi-Anion
				Hydrophobic	Pi-Alkyl
3.	Glucotropaeolin (9548605)	ARG220	3.05653	Hydrogen Bond	Conventional Hydrogen Bond
		SER196	2.98597	Hydrogen Bond	Conventional Hydrogen Bond
		SER205	2.40965	Hydrogen Bond	Conventional Hydrogen Bond
		SER205	2.12455	Hydrogen Bond	Conventional Hydrogen Bond
		CYS207	1.92817	Hydrogen Bond	Conventional Hydrogen Bond
		HIS209	3.71798	Hydrogen Bond	Carbon Hydrogen Bond
		ARG220	3.30573	Hydrogen Bond	Carbon Hydrogen Bond
		HIS209	4.76639	Hydrophobic	Pi-Pi T-shaped
4.	8S-HODE (16061037)	LYS311	3.1441	Hydrogen Bond	Conventional Hydrogen Bond
		THR339	2.90868	Hydrogen Bond	Conventional Hydrogen Bond
		SER291	2.14137	Hydrogen Bond	Conventional Hydrogen Bond
		ARG310	4.73147	Hydrophobic	Alkyl
		TYR292	5.18253	Hydrophobic	Pi-Alkyl
5.	3-Keto stearic acid (5283005)	ASN210	3.21746	Hydrogen Bond	Conventional Hydrogen Bond
		SER196	3.06542	Hydrogen Bond	Conventional Hydrogen Bond
		ASN210	2.24438	Hydrogen Bond	Conventional Hydrogen Bond
		CYS207	4.82762	Hydrophobic	Alkyl
		PRO219	4.74636	Hydrophobic	Alkyl
		CYS207	5.33324	Hydrophobic	Alkyl
		HIS209	4.78216	Hydrophobic	Pi-Alkyl
6.	16-Hydroxy hexadecanoic acid (10466)	CYS287	3.23648	Hydrogen Bond	Conventional Hydrogen Bond
		SER342	3.24817	Hydrogen Bond	Conventional Hydrogen Bond
		CYS287	2.30236	Hydrogen Bond	Conventional Hydrogen Bond
		ARG310	4.51743	Hydrophobic	Alkyl
		TYR292	4.47428	Hydrophobic	Pi-Alkyl
		TYR292	5.34053	Hydrophobic	Pi-Alkyl

7.	Benzyl isothiocyanate (2346)	PHE412<br>LEU382<br>ALA415<br>VAL417<br>ILE438	5.27686<br>5.40683<br>4.21322<br>5.13117<br>4.99917	Hydrophobic<br>Hydrophobic<br>Hydrophobic<br>Hydrophobic<br>Hydrophobic	Pi-Pi T-shaped<br>Pi-Alkyl<br>Pi-Alkyl<br>Pi-Alkyl<br>Pi-Alkyl
**Binding interaction of phytochemicals with HER3 (ERBB3)**					
1.	Quercetin 3-(2-caffeoylglucuronoside) (131753131)	CYS721<br>SER698<br>LEU771<br>GLY697<br>ARG819<br>ASN820<br>ASP833<br>LEU696<br>THR768<br>LEU696<br>ALA832	3.35693<br>2.92248<br>2.31282<br>2.91835<br>2.67679<br>2.33875<br>4.19788<br>3.97495<br>3.50371<br>4.62303<br>5.4699	Hydrogen Bond<br>Hydrogen Bond<br>Hydrogen Bond<br>Hydrogen Bond<br>Hydrogen Bond<br>Hydrogen Bond<br>Electrostatic<br>Hydrophobic<br>Hydrophobic<br>Hydrophobic<br>Hydrophobic	Conventional Hydrogen Bond<br>Conventional Hydrogen Bond<br>Conventional Hydrogen Bond<br>Conventional Hydrogen Bond<br>Conventional Hydrogen Bond<br>Conventional Hydrogen Bond<br>Pi-Anion<br>Pi-Sigma<br>Pi-Sigma<br>Pi-Alkyl<br>Pi-Alkyl
2.	(-)-Epicatechin 3'-O-glucuronide (76969982)	LYS723<br>ASN815<br>ARG819<br>ASP833<br>ASP833<br>SER698<br>THR768<br>GLN769<br>GLY699<br>ASN820<br>CYS721<br>LEU822<br>GLY697, SER698<br>VAL704<br>VAL704	2.82076<br>3.33639<br>2.74181<br>2.4855<br>2.49461<br>1.95695<br>2.39468<br>2.66636<br>3.44416<br>3.74799<br>3.83756<br>3.41431<br>4.84775<br>4.47578<br>5.23683	Hydrogen Bond<br>Hydrogen Bond<br>Hydrogen Bond<br>Hydrogen Bond<br>Hydrogen Bond<br>Hydrogen Bond<br>Hydrogen Bond<br>Hydrogen Bond<br>Hydrogen Bond<br>Hydrogen Bond<br>Hydrogen Bond; Other<br>Hydrophobic<br>Hydrophobic<br>Hydrophobic<br>Hydrophobic	Conventional Hydrogen Bond<br>Conventional Hydrogen Bond<br>Conventional Hydrogen Bond<br>Conventional Hydrogen Bond<br>Conventional Hydrogen Bond<br>Conventional Hydrogen Bond<br>Conventional Hydrogen Bond<br>Conventional Hydrogen Bond<br>Carbon Hydrogen Bond<br>Carbon Hydrogen Bond<br>Pi-Donor Hydrogen Bond; Pi-Sulfur<br>Pi-Sigma<br>Amide-Pi Stacked<br>Pi-Alkyl<br>Pi-Alkyl
3.	Glucotropaeolin (9548605)	THR768<br>SER698<br>ARG819<br>CYS721<br>LEU822	2.94449<br>2.17893<br>2.21344<br>3.86704<br>3.70247	Hydrogen Bond<br>Hydrogen Bond<br>Hydrogen Bond<br>Hydrogen Bond;Other<br>Hydrophobic	Conventional Hydrogen Bond<br>Conventional Hydrogen Bond<br>Conventional Hydrogen Bond<br>Pi-Donor Hydrogen Bond;Pi-Sulfur<br>Pi-Sigma
4.	8S-HODE (16061037)	LYS723<br>GLY699<br>VAL704<br>LYS723<br>VAL753<br>ALA832<br>LEU766<br>ILE744<br>LEU755<br>LEU766<br>PHE834	2.90542<br>3.6806<br>3.83045<br>4.66072<br>4.39374<br>4.06245<br>4.89253<br>5.23869<br>4.58429<br>4.68996<br>5.00289	Hydrogen Bond<br>Hydrogen Bond<br>Hydrophobic<br>Hydrophobic<br>Hydrophobic<br>Hydrophobic<br>Hydrophobic<br>Hydrophobic<br>Hydrophobic<br>Hydrophobic<br>Hydrophobic	Conventional Hydrogen Bond<br>Carbon Hydrogen Bond<br>Alkyl<br>Alkyl<br>Alkyl<br>Alkyl<br>Alkyl<br>Alkyl<br>Alkyl<br>Alkyl<br>Pi-Alkyl
5.	3-Keto	LYS723	3.20794	Hydrogen Bond	Conventional Hydrogen Bond

	stearic acid (5283005)	ASN815	3.05786	Hydrogen Bond	Conventional Hydrogen Bond
		ASN815	3.2164	Hydrogen Bond	Conventional Hydrogen Bond
		ARG819	2.99838	Hydrogen Bond	Conventional Hydrogen Bond
		ASN820	3.09104	Hydrogen Bond	Conventional Hydrogen Bond
		ASP833	1.83921	Hydrogen Bond	Conventional Hydrogen Bond
		VAL704	4.01014	Hydrophobic	Alkyl
		VAL704	5.18213	Hydrophobic	Alkyl
		CYS721	4.6947	Hydrophobic	Alkyl
		VAL753	5.256	Hydrophobic	Alkyl
		VAL753	5.27004	Hydrophobic	Alkyl
		ALA832	4.75718	Hydrophobic	Alkyl
		ALA832	4.685	Hydrophobic	Alkyl
		LEU822	5.31764	Hydrophobic	Alkyl
		LEU822	4.86165	Hydrophobic	Alkyl
		ILE744	5.24686	Hydrophobic	Alkyl
		LEU755	4.18122	Hydrophobic	Alkyl
		LEU766	4.77701	Hydrophobic	Alkyl
		PHE834	5.00963	Hydrophobic	Pi-Alkyl
6.	16-Hydroxy hexadecanoic acid (10466)	PHE834	3.22499	Hydrogen Bond	Conventional Hydrogen Bond
		ARG819	2.01636	Hydrogen Bond	Conventional Hydrogen Bond
		VAL704	4.53215	Hydrophobic	Alkyl
		VAL704	4.36141	Hydrophobic	Alkyl
		VAL704	4.3683	Hydrophobic	Alkyl
		CYS721	4.58303	Hydrophobic	Alkyl
		CYS721	4.9033	Hydrophobic	Alkyl
		LYS723	4.81444	Hydrophobic	Alkyl
		ALA832	5.17383	Hydrophobic	Alkyl
		LEU822	5.35367	Hydrophobic	Alkyl
		LEU696	5.282	Hydrophobic	Alkyl
		LEU822	5.11454	Hydrophobic	Alkyl
7.	Benzyl isothiocyanate (2346)	ASP833	3.13447	Electrostatic	Attractive Charge
		SER698	3.71204	Hydrogen Bond	Conventional Hydrogen Bond
		VAL704	3.98697	Hydrophobic	Pi-Sigma
		CYS721	5.48514	Hydrophobic	Pi-Alkyl
		LEU822	5.3812	Hydrophobic	Pi-Alkyl
**Binding interaction of phytochemicals with Membrane progesterone receptor alpha (mPRα)**					
1.	Quercetin 3-(2-caffeoylglucuronoside) (131753131)	ASN192	3.2416	Hydrogen Bond	Conventional Hydrogen Bond
		ASN192	3.22374	Hydrogen Bond	Conventional Hydrogen Bond
		LYS193	2.98351	Hydrogen Bond	Conventional Hydrogen Bond
		GLN206	3.06502	Hydrogen Bond	Conventional Hydrogen Bond
		LEU41	2.73076	Hydrogen Bond	Conventional Hydrogen Bond
		HIS135	2.89373	Hydrogen Bond	Conventional Hydrogen Bond
		HIS279	2.5284	Hydrogen Bond	Conventional Hydrogen Bond
		PRO45	3.41873	Hydrogen Bond	Carbon Hydrogen Bond
		ASP142	3.63861	Electrostatic	Pi-Anion
		PHE258	5.2197	Hydrophobic	Pi-Pi T-shaped
		PHE258	5.11287	Hydrophobic	Pi-Pi T-shaped
		PHE278	5.25596	Hydrophobic	Pi-Pi T-shaped
		LYS44	4.8853	Hydrophobic	Pi-Alkyl
		PRO45	5.03409	Hydrophobic	Pi-Alkyl
		LYS44	4.58673	Hydrophobic	Pi-Alkyl

		PRO45	5.18659	Hydrophobic	Pi-Alkyl
2.	(-)-Epicatechin 3'-O-glucuronide (76969982)	ASN192	3.04263	Hydrogen Bond	Conventional Hydrogen Bond
		ASN192	2.81138	Hydrogen Bond	Conventional Hydrogen Bond
		HIS275	3.31362	Hydrogen Bond	Conventional Hydrogen Bond
		LYS197	2.07049	Hydrogen Bond	Conventional Hydrogen Bond
		LYS197	2.81243	Hydrogen Bond	Conventional Hydrogen Bond
		GLY199	2.40003	Hydrogen Bond	Conventional Hydrogen Bond
		TYR143	2.52009	Hydrogen Bond	Conventional Hydrogen Bond
		PHE254	2.66468	Hydrogen Bond	Conventional Hydrogen Bond
		PHE255	2.29921	Hydrogen Bond	Conventional Hydrogen Bond
		PHE258	4.59335	Hydrophobic	Pi-Pi T-shaped
		HIS275	4.63063	Hydrophobic	Pi-Pi T-shaped
3.	Glucotropaeolin (9548605)	TYR143	3.21984	Hydrogen Bond	Conventional Hydrogen Bond
		TYR143	3.00901	Hydrogen Bond	Conventional Hydrogen Bond
		LYS193	2.96144	Hydrogen Bond	Conventional Hydrogen Bond
		LYS193	3.30285	Hydrogen Bond	Conventional Hydrogen Bond
		LYS193	2.93562	Hydrogen Bond	Conventional Hydrogen Bond
		LEU41	2.87076	Hydrogen Bond	Conventional Hydrogen Bond
		LYS44	4.66378	Hydrophobic	Pi-Alkyl
		PRO45	5.30397	Hydrophobic	Pi-Alkyl
4.	3-Keto stearic acid (5283005)	SER121	1.79922	Hydrogen Bond	Conventional Hydrogen Bond
		ASP142	2.63585	Hydrogen Bond	Conventional Hydrogen Bond
		HIS78	3.55948	Hydrogen Bond	Carbon Hydrogen Bond
		HIS275	3.67057	Hydrogen Bond	Carbon Hydrogen Bond
		VAL146	4.24779	Hydrophobic	Alkyl
		LEU282	4.7612	Hydrophobic	Alkyl
		VAL146	3.67691	Hydrophobic	Alkyl
		LEU282	4.59506	Hydrophobic	Alkyl
		TYR117	4.17559	Hydrophobic	Pi-Alkyl
		TYR149	4.59324	Hydrophobic	Pi-Alkyl
		PHE248	5.1127	Hydrophobic	Pi-Alkyl
		PHE255	4.49241	Hydrophobic	Pi-Alkyl
		PHE255	5.48787	Hydrophobic	Pi-Alkyl
		PHE278	5.49539	Hydrophobic	Pi-Alkyl
5.	8S-HODE (16061037)	SER121	3.03153	Hydrogen Bond	Conventional Hydrogen Bond
		HIS279	2.59201	Hydrogen Bond	Conventional Hydrogen Bond
		PHE139	5.39067	Hydrophobic	Pi-Alkyl
		TYR143	4.88592	Hydrophobic	Pi-Alkyl
		PHE255	5.33535	Hydrophobic	Pi-Alkyl
		PHE255	4.29285	Hydrophobic	Pi-Alkyl
		HIS275	5.33446	Hydrophobic	Pi-Alkyl
6.	Benzyl isothiocyanate (2346)	PHE278	5.19766	Other	Pi-Sulfur
		PHE248	4.93346	Hydrophobic	Pi-Pi T-shaped
7.	16-Hydroxy hexadecanoic acid (10466)	ALA81	3.11234	Hydrogen Bond	Conventional Hydrogen Bond
		TRP76	3.61334	Hydrogen Bond	Carbon Hydrogen Bond
		VAL73	3.86865	Hydrophobic	Alkyl
		ILE280	4.67413	Hydrophobic	Alkyl
		VAL283	4.42999	Hydrophobic	Alkyl
		LEU80	5.25066	Hydrophobic	Alkyl
		LEU284	5.00949	Hydrophobic	Alkyl

Binding interaction with G-Protein Coupled Estrogen Receptor (GPER / GPR30)					
1.	Quercetin 3-(2-caffeoylglucuronoside) (131753131)	GLN39	3.18726	Hydrogen Bond	Conventional Hydrogen Bond
		GLN39	2.874	Hydrogen Bond	Conventional Hydrogen Bond
		GLY182	2.84348	Hydrogen Bond	Conventional Hydrogen Bond
		TYR228	2.7944	Hydrogen Bond	Conventional Hydrogen Bond
		TYR228	2.34737	Hydrogen Bond	Conventional Hydrogen Bond
		GLU42	2.29881	Hydrogen Bond	Conventional Hydrogen Bond
		LYS244	4.95124	Electrostatic	Pi-Cation
		VAL226	3.75703	Hydrophobic	Pi-Sigma
		VAL226	3.93376	Hydrophobic	Pi-Sigma
		VAL226	3.74484	Hydrophobic	Pi-Sigma
		LYS244	4.96058	Hydrophobic	Pi-Alkyl
		LYS244	5.48928	Hydrophobic	Pi-Alkyl
		PRO181	5.15602	Hydrophobic	Pi-Alkyl
		LYS244	4.47262	Hydrophobic	Pi-Alkyl
2.	(-)-Epicatechin 3'-O-glucuronide (76969982)	ARG180	3.00691	Hydrogen Bond	Conventional Hydrogen Bond
		ARG180	3.11379	Hydrogen Bond	Conventional Hydrogen Bond
		GLY198	2.26465	Hydrogen Bond	Conventional Hydrogen Bond
		ASP109	2.61247	Hydrogen Bond	Conventional Hydrogen Bond
		PRO185	2.33314	Hydrogen Bond	Conventional Hydrogen Bond
		GLN183	2.12018	Hydrogen Bond	Conventional Hydrogen Bond
		GLN183	3.71006	Hydrogen Bond	Pi-Donor Hydrogen Bond
3.	Glucotropaeolin (9548605)	THR330	3.09845	Hydrogen Bond	Conventional Hydrogen Bond
		TYR324	2.48533	Hydrogen Bond	Conventional Hydrogen Bond
		ARG155	3.85243	Hydrogen Bond	Pi-Donor Hydrogen Bond
		PHE98	5.89199	Other	Pi-Sulfur
		PRO94	3.88858	Hydrophobic	Pi-Alkyl
		ARG155	4.05629	Hydrophobic	Pi-Alkyl
4.	8S-HODE (16061037)	TRP150	3.32493	Hydrogen Bond	Pi-Donor Hydrogen Bond
		ALA184	4.15578	Hydrophobic	Alkyl
		ILE181	5.20292	Hydrophobic	Alkyl
		ILE229	4.10134	Hydrophobic	Alkyl
		ILE229	3.89167	Hydrophobic	Alkyl
		LEU232	5.00906	Hydrophobic	Alkyl
		CYS233	4.73542	Hydrophobic	Alkyl
		PHE153	4.67058	Hydrophobic	Pi-Alkyl
5.	16-Hydroxy hexadecanoic acid (10466)	LEU64	4.87661	Hydrophobic	Alkyl
		LEU69	4.686	Hydrophobic	Alkyl
		ALA312	5.29934	Hydrophobic	Alkyl
		LEU61	4.88342	Hydrophobic	Alkyl
		LEU311	4.23318	Hydrophobic	Alkyl
		LEU64	4.83059	Hydrophobic	Alkyl
		PHE68	3.75223	Hydrophobic	Pi-Alkyl
6.	Benzyl isothiocyanate (2346)	ASP62	5.29119	Electrostatic	Attractive Charge
		TYR59	4.04117	Hydrophobic	Pi-Pi Stacked
7.	3-Keto stearic acid (5283005)	GLY231	3.47066	Hydrogen Bond	Carbon Hydrogen Bond
		VAL265	4.83022	Hydrophobic	Alkyl
		LEU266	5.21663	Hydrophobic	Alkyl
		LEU266	4.80145	Hydrophobic	Alkyl
		PHE269	4.36011	Hydrophobic	Pi-Alkyl
		PHE269	4.6159	Hydrophobic	Pi-Alkyl

Table S6. Molecular docking and interaction studies of chemical compounds with reference drug Tamoxifen (or 4-hydroxytamoxifen)

**PyRx Binding Affinity**	**3D interaction**	**2D Interaction**	Interacting Amino acids	Distance (Å)	Category	Types
**Estrogen Receptor α (ERα / ESR1) 1ERR**						
-7.7	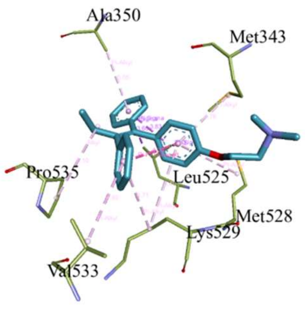	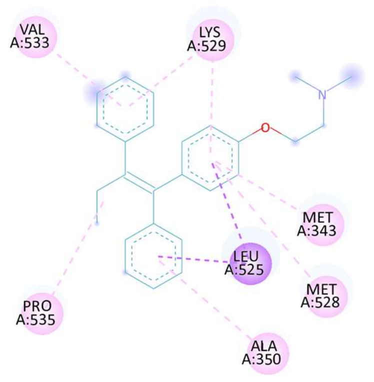	LEU525	3.93093	Hydrophobic	Pi-Sigma
			LEU525	3.82571	Hydrophobic	Pi-Sigma
			LEU525	3.66188	Hydrophobic	Pi-Sigma
			PRO535	5.10041	Hydrophobic	Alkyl
			MET343	4.76142	Hydrophobic	Pi-Alkyl
			MET528	5.15274	Hydrophobic	Pi-Alkyl
			LYS529	5.39549	Hydrophobic	Pi-Alkyl
			ALA350	5.04501	Hydrophobic	Pi-Alkyl
			LYS529	4.71495	Hydrophobic	Pi-Alkyl
			VAL533	4.79546	Hydrophobic	Pi-Alkyl
**Progesterone receptor**						

-7.9	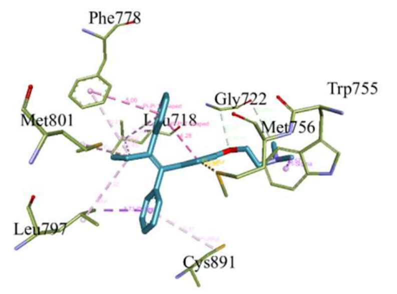	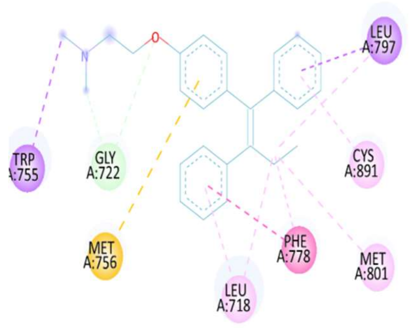	GLY722	3.29822	Hydrogen Bond	Carbon Hydrogen Bond
			GLY722	3.7586	Hydrogen Bond	Carbon Hydrogen Bond
			LEU797	3.7308	Hydrophobic	Pi-Sigma
			TRP755	3.56865	Hydrophobic	Pi-Sigma
			MET756	5.94517	Other	Pi-Sulfur
			PHE778	4.99607	Hydrophobic	Pi-Pi T-shaped
			LEU718	4.47555	Hydrophobic	Alkyl
			LEU797	5.32114	Hydrophobic	Alkyl
			MET801	5.13504	Hydrophobic	Alkyl
			PHE778	5.20547	Hydrophobic	Pi-Alkyl
			CYS891	5.11409	Hydrophobic	Pi-Alkyl
			LEU718	4.99209	Hydrophobic	Pi-Alkyl
**Insulin-Like Growth Factor-1 Receptor (IGF-1R)**						

-7.2	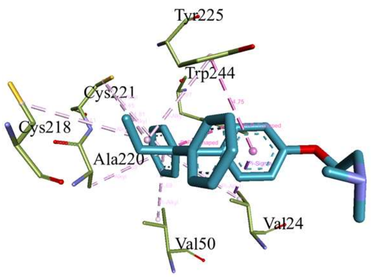	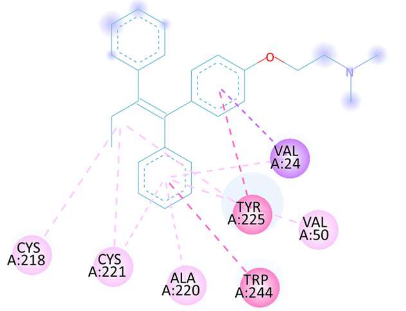	VAL24	3.96712	Hydrophobic	Pi-Sigma
			TYR225	4.74869	Hydrophobic	Pi-Pi T-shaped
			TRP244	4.67092	Hydrophobic	Pi-Pi T-shaped
			CYS218	5.41435	Hydrophobic	Alkyl
			CYS221	5.44949	Hydrophobic	Alkyl
			TYR225	4.97014	Hydrophobic	Pi-Alkyl
			VAL24	5.16486	Hydrophobic	Pi-Alkyl
			VAL50	4.69119	Hydrophobic	Pi-Alkyl
			ALA220	4.39347	Hydrophobic	Pi-Alkyl
			CYS221	4.80633	Hydrophobic	Pi-Alkyl
**Epidermal Growth Factor Receptor (EGFR / ERBB1)**						
-6.8	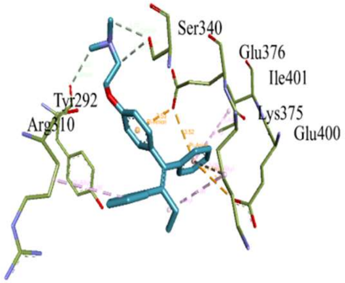	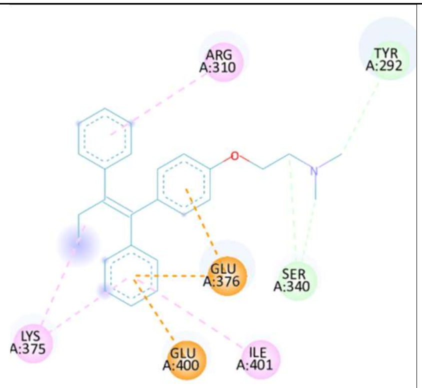	SER340	3.73635	Hydrogen Bond	Carbon Hydrogen Bond
			TYR292	3.64989	Hydrogen Bond	Carbon Hydrogen Bond
			SER340	3.76994	Hydrogen Bond	Carbon Hydrogen Bond
			GLU376	3.53421	Electrostatic	Pi-Anion
			GLU376	3.52028	Electrostatic	Pi-Anion
			GLU400	3.71001	Electrostatic	Pi-Anion

			LYS375	4.86275	Hydrophobic	Alkyl
			LYS375	5.05806	Hydrophobic	Pi-Alkyl
			ILE401	5.14671	Hydrophobic	Pi-Alkyl
			ARG310	5.06141	Hydrophobic	Pi-Alkyl
**HER3 (ERBB3)**						
-7.5	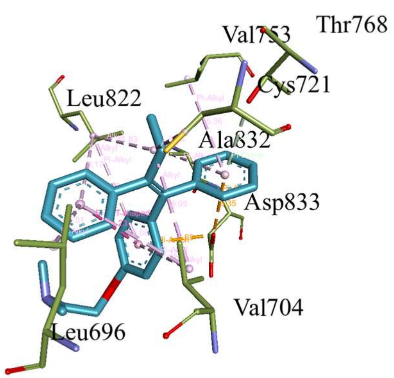	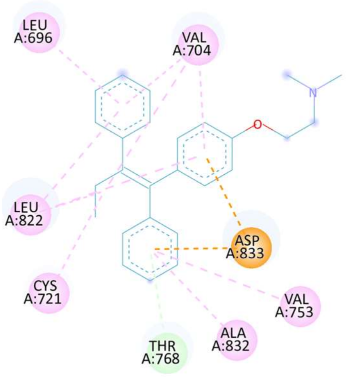	ASP833	3.69399	Electrostatic	Pi-Anion
			ASP833	3.34544	Electrostatic	Pi-Anion
			THR768	3.76989	Hydrogen Bond	Pi-Donor Hydrogen Bond
			VAL704	5.09296	Hydrophobic	Alkyl
			CYS721	3.54799	Hydrophobic	Alkyl
			LEU822	4.82613	Hydrophobic	Alkyl
			VAL704	5.31927	Hydrophobic	Pi-Alkyl
			LEU822	5.49635	Hydrophobic	Pi-Alkyl
			VAL753	5.36381	Hydrophobic	Pi-Alkyl
			ALA832	4.61878	Hydrophobic	Pi-Alkyl
			LEU696	5.00945	Hydrophobic	Pi-Alkyl
			VAL704	5.07576	Hydrophobic	Pi-Alkyl

			LEU822	5.16899	Hydrophobic	Pi-Alkyl
**Membrane progesterone receptor alpha (mPRα)**						
-7.9	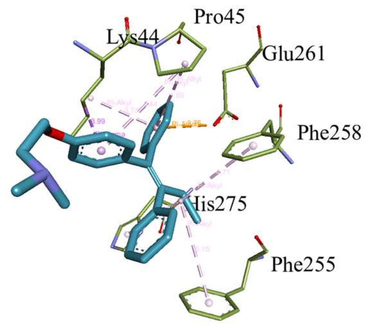	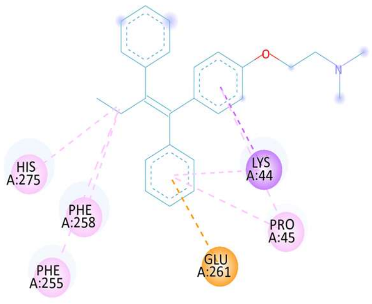	GLU261	3.74866	Electrostatic	Pi-Anion
			LYS44	3.98623	Hydrophobic	Pi-Sigma
			PHE255	5.14842	Hydrophobic	Pi-Alkyl
			PHE258	4.71294	Hydrophobic	Pi-Alkyl
			HIS275	4.51825	Hydrophobic	Pi-Alkyl
			PRO45	5.43558	Hydrophobic	Pi-Alkyl
			LYS44	3.73407	Hydrophobic	Pi-Alkyl
			PRO45	4.85164	Hydrophobic	Pi-Alkyl
**G-Protein Coupled Estrogen Receptor (GPER / GPR30)**						

-7.0	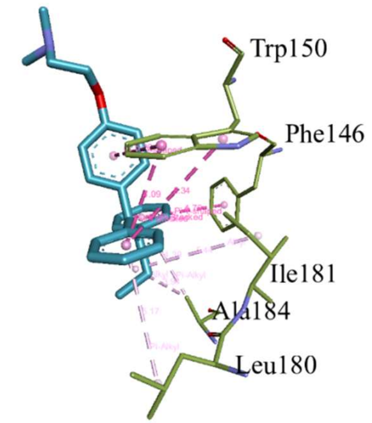	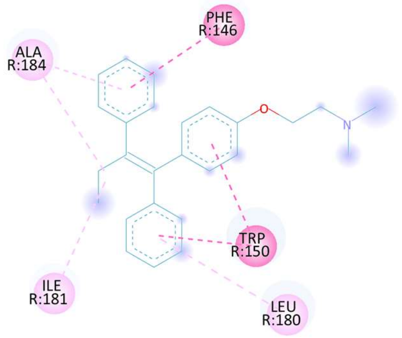	TRP150<br>TRP150<br>PHE146<br>TRP150<br>ALA184<br>ILE181<br>LEU180<br>ALA184	5.33665<br>4.09195<br>4.75834<br>5.22431<br>3.93617<br>5.44143<br>5.16533<br>4.38951	Hydrophobic<br>Hydrophobic<br>Hydrophobic<br>Hydrophobic<br>Hydrophobic<br>Hydrophobic<br>Hydrophobic<br>Hydrophobic	Pi-Pi Stacked<br>Pi-Pi Stacked<br>Pi-Pi T-shaped<br>Pi-Pi T-shaped<br>Alkyl<br>Alkyl<br>Pi-Alkyl<br>Pi-Alkyl

Table S7. Physicochemical Properties of reference drug Tamoxifen

S.No.	Reference drug	Formula	MW (g/mol)	2-D structure	Heavy atoms	Aromatic heavy atoms	Fraction Csp3	Rotatable bonds	H-bond acceptors	H-bond donors	MR	TPSA
1	Tamoxifen (or 4-hydroxytamoxifen)	$C_{26}H_{29}NO$	371.51	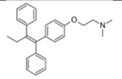	28	18	0.23	8	2	0	119.72	12.47

Table S8. Lipophilicity Properties of *Salvadora persica L.* (Salvadoraceae) root phytoconstituents obtained through HRLC-MS/MS at positive ion mode

S. No.	Compounds Name (PubChem CID)	iLOGP	XLOGP3	WLOGP	MLOGP	Silicos-IT Log P	Consensus Log P
1	Medicanine (101409750)	1.2	-2.58	-0.85	-0.5	-0.19	-0.58
2	3beta,6beta Dihydroxynortropane (22297531)	1.12	-0.69	-1.15	-0.31	-0.17	-0.24
3	N-Acetyl-leucyl-leucin (443129)	2.07	0.53	1.15	0.92	1.44	1.22
4	Leucyl-Histidine (6992828)	0.77	-2.17	-0.1	-0.94	0.59	-0.37
5	p-Coumaroylagmatine (440362)	1.46	0.44	0.47	0.93	1.18	0.9
6	5-Methoxydimethyltryptamine (1832)	2.54	2.79	2.28	1.5	2.79	2.38
7	N(alpha)-gamma-L Glutamylhistamine (440238)	0.18	-3.63	-0.74	-3.73	0.16	-1.55
8	Europine (5462451)	2.65	-0.49	-0.94	-0.48	0.12	0.17
9	(+)-alpha-Pinene (82227)	2.63	4.48	3	4.29	2.79	3.44
10	Asparaginyl-Cysteine (18218178)	0.32	-4.85	-2.31	-1.94	-1.66	-2.09
11	Macamide B (11198769)	4.99	8.36	6.63	5.09	7.31	6.48
12	Benzoxazinone glucoside/ 2,4-Dihydroxy-7,8-dimethoxy-2H-1,4-benzoxazin-3(4H)-one 2-glucoside (77195081)	1.62	-1.64	-2.42	-2.23	-2.57	-1.45
13	N-(14-Methylhexadecanoyl)pyrrolidine (6430518)	5.09	7.79	5.96	4.56	6.59	6
14	Pipercitine (12575258)	5.5	8.86	6.66	4.89	7.29	6.64
15	Benzyl isothiocyanate (2346)	2.19	3.16	2.14	3.28	3.79	2.91

Table S9. Lipophilicity properties of *Salvadora persica* root phytoconstituents obtained through HRLC-MS/MS at negative ion mode

S. No.	Molecule	iLOGP	XLOGP3	WLOGP	MLOGP	Silicos-IT Log P	Consensus Log P
**1**	Glucotropaeolin	0.45	-0.22	-0.02	-1.65	-1.73	-0.63
**2**	Quercetin 3-(2-caffeoylglucuronoside)	1.86	2.56	1.12	-1.97	0.18	0.75
**3**	(-)-Epicatechin 3'-O-glucuronide	0.74	-1.2	-1.21	-1.86	-1.62	-1.03
**4**	Benzyl isothiocyanate	2.19	3.16	2.14	3.28	3.79	2.91
**5**	8S-HODE	2.19	3.16	4.86	3.28	3.79	2.91
**6**	16-Hydroxy hexadecanoic acid	3.39	5.6	4.52	3.31	4.69	4.3
**7**	3-Keto stearic acid	3.6	6.97	5.51	3.69	5.72	5.1

Table S10. Lipophilicity properties of reference drug Tamoxifen

Reference drug	iLOGP	XLOGP3	WLOGP	MLOGP	Silicos-IT Log P	Consensus Log P
Tamoxifen (or 4-hydroxytamoxifen)	n/d	7.14	6	5.1	5.99	NA

Table S11. Water solubility properties of *Salvadora persica L.* (Salvadoraceae) root phytoconstituents obtained through HRLC-MS/MS at positive ion mode

S. No.	Compounds Name (PubChem CID)	ESOL Log S	ESOL Solubility (mg/ml)	ESOL Solubility (mol/l)	ESOL Class	Ali Log S	Ali Solubility (mg/ml)	Ali Solubility (mol/l)	Ali Class	Silicos-IT LogSw	Silicos-IT Solubility (mg/ml)	Silicos-IT Solubility (mol/l)	Silicos-IT class
1	Medicanine (101409750)	1.06	1.84E+03	1.15E+01	Highly soluble	1.85	1.13E+04	7.08E+01	Highly soluble	0.18	2.43E+02	1.52E+00	Soluble
2	3beta,6beta Dihydroxynortropane (22297531)	-0.29	7.29E+01	5.09E-01	Very soluble	0.06	1.65E+02	1.15E+00	Highly soluble	0.23	2.41E+02	1.68E+00	Soluble
3	N-Acetyl-leucyl-leucin (443129)	-1.29	1.47E+01	5.14E-02	Very soluble	-2.11	2.24E+00	7.82E-03	Soluble	-2.19	1.85E+00	6.46E-03	Soluble
4	Leucyl-Histidine (6992828)	0.2	4.22E+02	1.57E+00	Highly soluble	0.16	3.86E+02	1.44E+00	Highly soluble	-1.98	2.80E+00	1.04E-02	Soluble
5	p-Coumaroylagmatine (440362)	-1.52	8.26E+00	2.99E-02	Very soluble	-2.4	1.11E+00	4.02E-03	Soluble	-2.91	3.43E-01	1.24E-03	Soluble
6	5-Methoxydimethyltryptamine (1832)	-3.1	1.72E-01	7.88E-04	Soluble	-3.04	1.99E-01	9.12E-04	Soluble	-4.34	1.00E-02	4.58E-05	Moderately soluble
7	N(alpha)-gamma-L Glutamylhistamine (440238)	1.27	4.45E+03	1.85E+01	Highly soluble	1.67	1.13E+04	4.70E+01	Highly soluble	-1.93	2.86E+00	1.19E-02	Soluble
8	Europine (5462451)	-1.11	2.55E+01	7.74E-02	Very soluble	-1.13	2.43E+01	7.39E-02	Very soluble	-0.22	1.97E+02	5.97E-01	Soluble
9	(+)-alpha-Pinene (82227)	-3.51	4.24E-02	3.11E-04	Soluble	-4.2	8.59E-03	6.31E-05	Moderately soluble	-2.23	8.06E-01	5.92E-03	Soluble
10	Asparaginyl-Cysteine (18218178)	2.22	3.89E+04	1.66E+02	Highly soluble	1.82	1.56E+04	6.62E+01	Highly soluble	0.49	7.27E+02	3.09E+00	Soluble

11	Macamide B (11198769)	-6.3	1.71E-04	4.96E-07	Poorly soluble	-8.84	5.02E-07	1.45E-09	Poorly soluble	-8.82	5.23E-07	1.51E-09	Poorly soluble
12	Benzoxazinone glucoside/ 2,4-Dihydroxy-7,8-dimethoxy-2H-1,4-benzoxazin-3(4H)-one 2-glucoside (77195081)	-1.14	2.95E+01	7.31E-02	Very soluble	-1.37	1.72E+01	4.27E-02	Very soluble	0.9	3.17E+03	7.85E+00	Soluble
13	N-(14-Methylhexadecanoyl)pyrrolidine (6430518)	-5.76	5.57E-04	1.72E-06	Moderately soluble	-8.06	2.81E-06	8.68E-09	Poorly soluble	-6.34	1.49E-04	4.61E-07	Poorly soluble
14	Pipercitine (12575258)	-6.53	1.02E-04	2.93E-07	Poorly soluble	-9.17	2.35E-07	6.73E-10	Poorly soluble	-6.66	7.68E-05	2.20E-07	Poorly soluble
15	Benzyl isothiocyanate (2346)	-3.07	1.28E-01	8.55E-04	Soluble	-3.76	2.57E-02	1.72E-04	Soluble	-2.98	1.57E-01	1.05E-03	Soluble

Table S12. Water solubility properties of *Salvadora persica* root phytoconstituents obtained through HRLC-MS/MS at negative ion mode

S. No.	Molecule	ESOL Log S	ESOL Solubility (mg/ml)	ESOL Solubility (mol/l)	ESOL Class	Ali Log S	Ali Solubility (mg/ml)	Ali Solubility (mol/l)	Ali Class	Silicos-IT LogSw	Silicos-IT Solubility (mg/ml)	Silicos-IT Solubility (mol/l)	Silicos-IT class
**1**	Glucotropaeolin	-1.95	4.61E+00	1.13E-02	Very soluble	-3.52	1.24E-01	3.03E-04	Soluble	-0.09	3.31E+02	8.09E-01	Soluble

**2**	Quercetin 3-(2-caffeoylglucuronoside)	-5.25	3.60E-03	5.63E-06	Moderately soluble	-7.96	6.96E-06	1.09E-08	Poorly soluble	-2.56	1.77E+00	2.77E-03	Soluble
**3**	(-)-Epicatechin 3'-O-glucuronide	-1.98	4.88E+00	1.05E-02	Very soluble	-2.64	1.06E+00	2.27E-03	Soluble	0.05	5.26E+02	1.13E+00	Soluble
**4**	Benzyl isothiocyanate	-3.07	1.28E-01	8.55E-04	Soluble	-3.76	2.57E-02	1.72E-04	Soluble	-2.98	1.57E-01	1.05E-03	Soluble
**5**	8S-HODE	-3.07	1.28E-01	8.55E-04	Soluble	-3.76	2.57E-02	1.72E-04	Soluble	-2.98	1.57E-01	1.05E-03	Soluble
**6**	16-Hydroxy hexadecanoic acid	-4.07	2.33E-02	8.57E-05	Moderately soluble	-6.57	7.32E-05	2.69E-07	Poorly soluble	-4.75	4.84E-03	1.78E-05	Moderately soluble
**7**	3-Keto stearic acid	-5.03	2.81E-03	9.43E-06	Moderately soluble	-7.93	3.54E-06	1.19E-08	Poorly soluble	-5.65	6.67E-04	2.23E-06	Moderately soluble

Table S13. Water solubility properties of reference drug Tamoxifen

S. No.	Reference drug	ESOL Log S	ESOL Solubility (mg/ml)	ESOL Solubility (mol/l)	ESOL Class	Ali Log S	Ali Solubility (mg/ml)	Ali Solubility (mol/l)	Ali Class	Silicos-IT LogSw	Silicos-IT Solubility (mg/ml)	Silicos-IT Solubility (mol/l)	Silicos-IT class
1	Tamoxifen (or 4-hydroxytamoxifen)	-6.59	9.56E-05	2.57E-07	Poorly soluble	-7.22	2.23E-05	5.99E-08	Poorly soluble	-8.92	4.48E-07	1.21E-09	Poorly soluble

Table S14. Pharmacokinetic properties of the reference drug Tamoxifen.

S.No.	Reference drug	GI absorption	BBB permeant	Pgp substrate	CYP1A2 inhibitor	CYP2C19 inhibitor	CYP2C9 inhibitor	CYP2D6 inhibitor	CYP3A4 inhibitor	log Kp (cm/s)
1	Tamoxifen (or 4-hydroxytamoxifen)	Low	No	n/d	n/d	n/d	n/d	n/d	n/d	-3.5

Table S15. Drug likeness Properties of *Salvadora persica L.* (Salvadoraceae) root phytoconstituents obtained through HRLC-MS/MS at positive ion mode

S. No.	Compounds Name (PubChem CID)	Lipinski violations	Ghose violations	Veber violations	Egan violations	Muegge violations	Bioavailability Score
1	Medicanine (101409750)	0	2	0	0	2	0.55

2	3beta,6beta Dihydroxynortropane (22297531)	0	2	0	0	1	0.55
3	N-Acetyl-leucyl-leucin (443129)	0	0	0	0	0	0.56
4	Leucyl-Histidine (6992828)	0	0	0	0	1	0.55
5	p-Coumaroylagmatine (440362)	0	0	0	0	0	0.55
6	5-Methoxydimethyltryptamine (1832)	0	0	0	0	0	0.55
7	N(alpha)-gamma-L Glutamylhistamine (440238)	0	1	0	0	1	0.55
8	Europine (5462451)	0	1	0	0	0	0.55
9	(+)-alpha-Pinene (82227)	1	1	0	0	2	0.55
10	Asparaginyl-Cysteine (18218178)	0	1	1	1	2	0.55
11	Macamide B (11198769)	1	1	1	1	2	0.55
12	Benzoxazinone glucoside/ 2,4-Dihydroxy-7,8-dimethoxy-2H-1,4-benzoxazin-3(4H)-one 2-glucoside (77195081)	1	1	1	1	2	0.55
13	N-(14-Methylhexadecanoyl)pyrrolidine (6430518)	1	1	1	1	1	0.55
14	Pipercitine (12575258)	1	1	1	1	2	0.55
15	Benzyl isothiocyanate (2346)	0	2	0	0	1	0.55

Table S16. Druglikeness Properties of *Salvadora persica* Root phytoconstituents obtained through HRLC-MS/MS at Negative ion mode

S. No.	Molecule	Lipinski #violations	Ghose #violations	Veber #violations	Egan #violations	Muegge #violations	Bioavailability Score
**1**	Glucotropaeolin	0	0	1	1	1	0.11

**2**	Quercetin 3-(2-caffeoylglucuronoside)	3	2	1	1	4	0.11
**3**	(-)-Epicatechin 3'-O-glucuronide	2	1	1	1	3	0.11
**4**	Benzyl isothiocyanate	0	2	0	0	1	0.55
**5**	8S-HODE	0	2	0	0	1	0.55
**6**	16-Hydroxy hexadecanoic acid	0	0	1	0	1	0.85
**7**	3-Keto stearic acid	0	0	1	0	2	0.85

Table S17. Drug likeness properties of reference drug Tamoxifen

S.No.	Reference drug	Lipinski violations	Ghose violations	Veber violations	Egan violations	Muegge violations	Bioavailability Score	Lipinski violations	Ghose violations	Veber violations
1	Tamoxifen (or 4-hydroxytamoxifen)	1	1	0	1	1	0.55	1	1	0

Table S18. Medicinal chemistry properties of *Salvadora persica L.* (Salvadoraceae) root phytoconstituents obtained through HRLC-MS/MS at positive ion mode.

S. No.	Compounds Name (PubChem CID)	PAINS alerts	Brenk alerts	Leadlikeness violations	Synthetic Accessibility
1	Medicanine (101409750)	0	0	1	1.99
2	3beta,6beta Dihydroxynortropane (22297531)	0	0	1	3.46
3	N-Acetyl-leucyl-leucin (443129)	0	0	1	2.94
4	Leucyl-Histidine (6992828)	0	0	1	3.14
5	p-Coumaroylagmatine (440362)	0	3	1	2.55
6	5-Methoxydimethyltryptamine (1832)	0	0	1	1.78
7	N(alpha)-gamma-L Glutamylhistamine (440238)	0	0	2	2.54
8	Europine (5462451)	0	1	0	4.79
9	(+)-alpha-Pinene (82227)	0	1	2	4.44
10	Asparaginyl-Cysteine (18218178)	0	1	1	2.8
11	Macamide B (11198769)	0	0	2	2.38
12	Benzoxazinone glucoside/ 2,4-Dihydroxy-7,8-dimethoxy-2H-1,4-benzoxazin-3(4H)-one 2-glucoside (77195081)	0	1	1	5.03
13	N-(14-Methylhexadecanoyl)pyrrolidine (6430518)	0	0	2	3.05
14	Pipercitine (12575258)	0	1	2	3.59
15	Benzyl isothiocyanate (2346)	0	2	1	1.59

Table S19. Medicinal Chemistry Properties of *Salvadora persica* Root phytoconstituents obtained through HRLC-MS/MS at Negative ion mode.

S. No.	Molecule	PAINS alerts	Brenk alerts	Leadlikeness violations	Synthetic Accessibility
**1**	Glucotropaeolin	0	4	1	5.27
**2**	Quercetin 3-(2-caffeoylglucuronoside)	1	2	2	6.02
**3**	(-)-Epicatechin 3'-O-glucuronide	0	0	1	5.23
**4**	Benzyl isothiocyanate	0	2	1	1.59
**5**	8S-HODE	0	2	1	1.59
**6**	16-Hydroxy hexadecanoic acid	0	0	2	2.35
**7**	3-Keto stearic acid	0	1	2	2.61

Table S20. Medicinal Chemistry properties of the reference drug Tamoxifen.

Reference drug	PAINS alerts	Brenk alerts	Leadlikeness violations	Synthetic Accessibility
Tamoxifen (or 4-hydroxytamoxifen)	0	1	3	3.01

Table S21. Toxicity potential analysis of reference drug Tamoxifen

Reference drug	Mutagenic	Tumorigenic	Irritant	Reproductive effect	Drug likeness
Tamoxifen (or 4-hydroxytamoxifen)	Green	Green	Green	Red	6.3

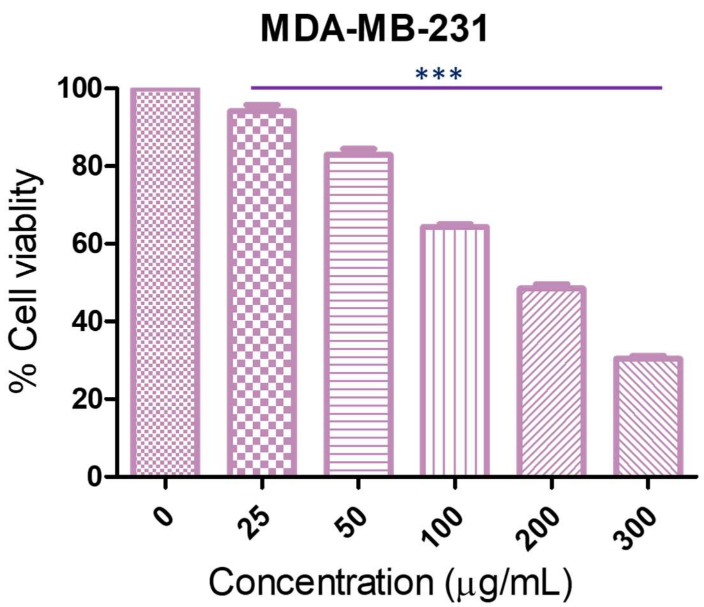

Figure S1. Cytotoxic test of *S. persica* root extract on human TNBC breast cancer MDA-MB-231 cells. Cytotoxicity of root extract was quantified as the percentage cell viability at 24 h. Values from a minimum of three independent experiments are shown as Mean ± SD, with ***$p<0.001$ in comparison to the control.

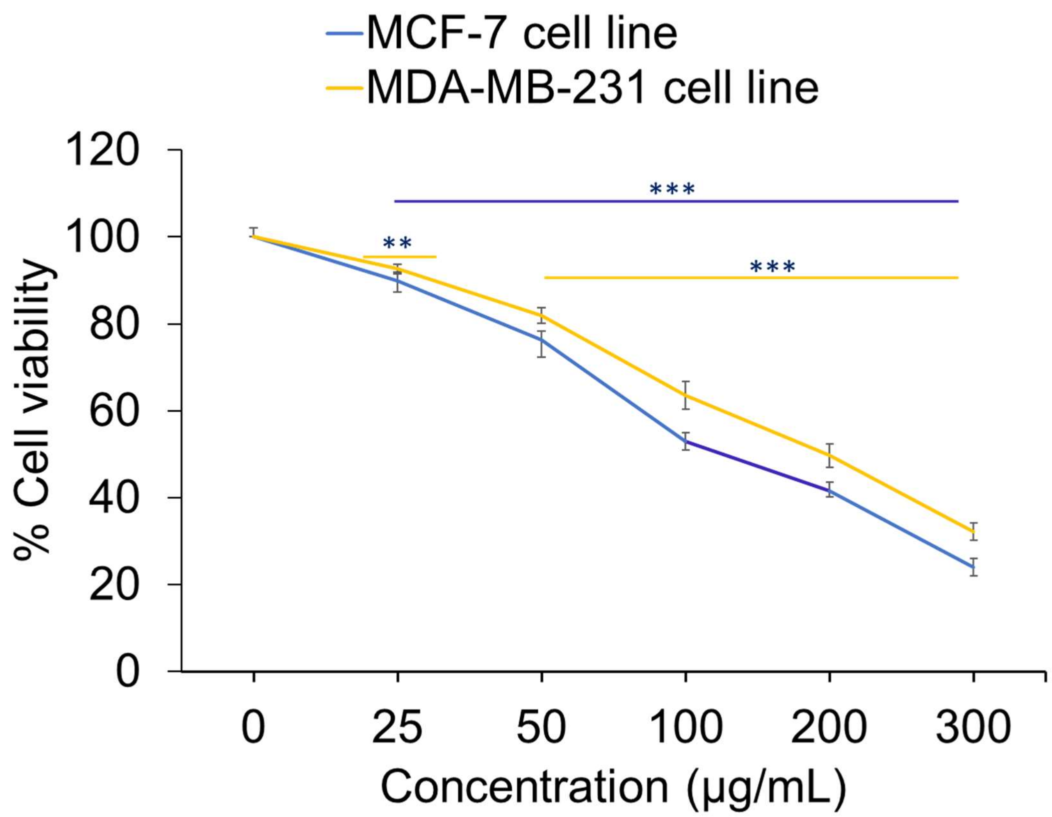

Figure S2. Effect of *S. persica* root extract on the viability of MCF-7 and MDA-MB-231 cells determined by the Trypan Blue exclusion assay. Cells were treated with different concentrations of the extract, and viable cells were quantified after treatment. Data are presented as mean ± SD from three independent experiments. Statistical significance was determined relative to the untreated control group, where **$p < 0.01$ and ***$p < 0.001$ for MDA-MB-231 cells, and ***$p < 0.001$ for MCF-7 cells.
